# Microneedle delivery of CAR-M-like engineered macrophages alleviates intervertebral disc degeneration through enhanced efferocytosis capacity

**DOI:** 10.1016/j.xcrm.2026.102620

**Published:** 2026-01-22

**Authors:** Xingyu Zhou, Dingchao Zhu, Di Wu, Gaocai Li, Huaizhen Liang, Weifeng Zhang, Yali Wu, Hanpeng Xu, Zhengdong Zhang, Bide Tong, Yu Song, Kun Wang, Xiaobo Feng, Jie Lei, Hongchuan Wang, Xiaoguang Zhang, Liang Ma, Yuhang Chen, Junyu Wei, Zixuan Ou, Shuchang Peng, Xinghuo Wu, Lei Tan, Bingjin Wang, Cao Yang

## Main text

(Cell Reports Medicine *6*, 102079; April 15, 2025)

In the originally published version of this article, the authors inadvertently used the incorrect MRI image in the rat “Degenerated” group of Figure S10A. The figure has now been corrected online. The authors regret this error, which did not alter any results, interpretations, or conclusions of the study.

**Figure S10A:**
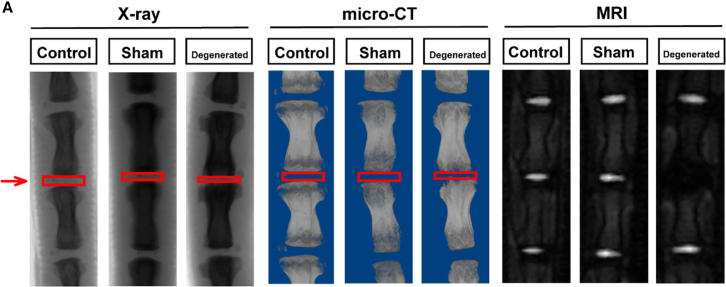
(corrected)

**Figure S10A:**
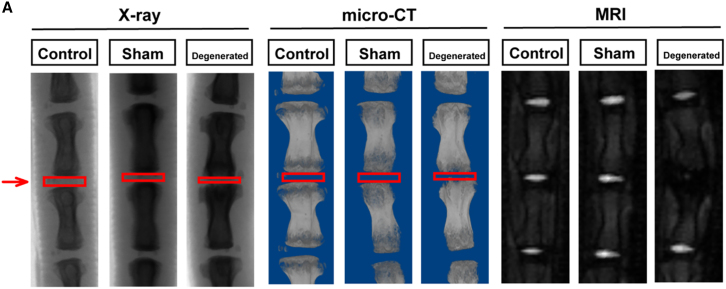
(original)

