# Supplementary material for: Microneedle delivery of CAR-M-like engineered macrophages alleviates intervertebral disc degeneration through enhanced efferocytosis capacity
Source: Cell Rep Med. 2025 Apr 7;6(4):102079. doi: 10.1016/j.xcrm.2025.102079 (PMC12047514; doi:10.1016/j.xcrm.2025.102079)
Supplement: Document S1. Figures S1–S12 and Tables S1–S3 [file mmc1.pdf]

Cell Reports Medicine, Volume *6*

Supplemental information

Microneedle delivery of CAR-M-like engineered macrophages alleviates intervertebral disc degeneration through enhanced efferocytosis capacity

Xingyu Zhou, Dingchao Zhu, Di Wu, Gaocai Li, Huaizhen Liang, Weifeng Zhang, Yali Wu, Hanpeng Xu, Zhengdong Zhang, Bide Tong, Yu Song, Kun Wang, Xiaobo Feng, Jie Lei, Hongchuan Wang, Xiaoguang Zhang, Liang Ma, Yuhang Chen, Junyu Wei, Zixuan Ou, Shuchang Peng, Xinghuo Wu, Lei Tan, Bingjin Wang, and Cao Yang

Supplemental Information

This file includes:

Figure S1 to S12

Table S1 to S3

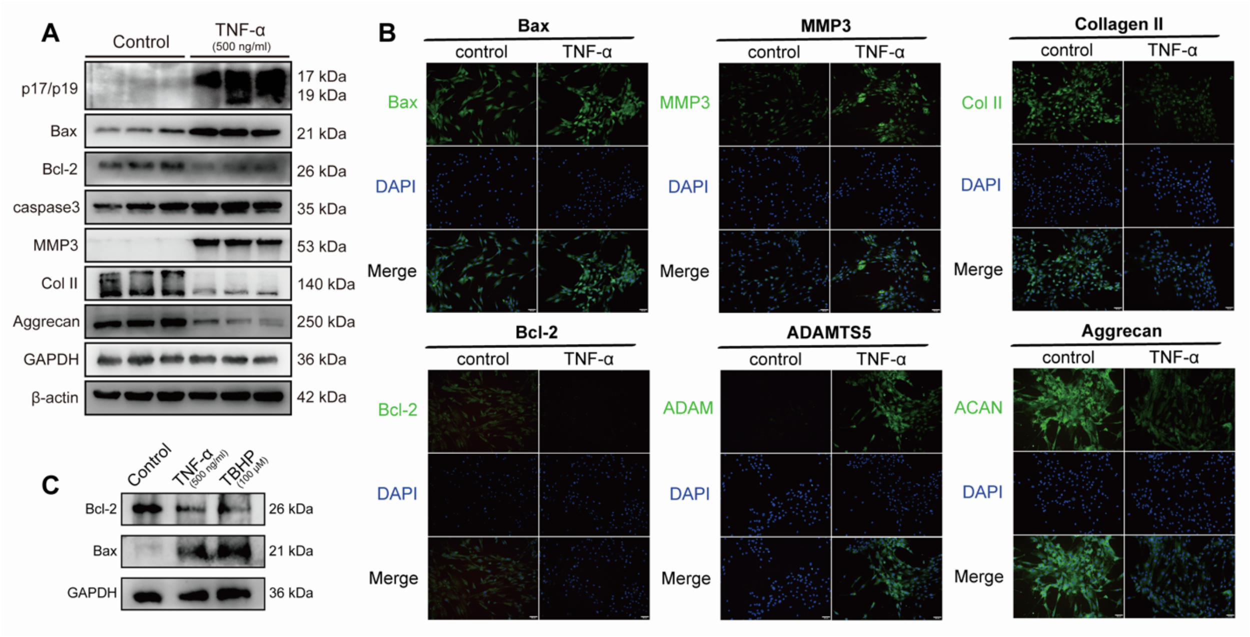

Figure S1. Establishment of apo-NPCs model, related to Figure 1 and the method detail, 'Cell culture and treatments' in STAR Methods.

(A) Representative western blots showing expression of Bax, Bcl-2, caspase3/p17/p19, MMP3, collagen type II and aggrecan in human NPCs treated with 500 ng/mL TNF-α for 24 h. (B) IF staining of Bax, Bcl-2, MMP3, ADAMTS5, Collagen type II, Aggrecan reflecting the success of apo-NPCs model establishment with the treatment of 500ng/mL TNF-α for 24 hours. (C) Representative western blots showing expression of Bax and Bcl-2 in human NPCs treated with 500 ng/mL TNF-α or 100 μM TBHP for 24 h. Scale bars = 100 μm.

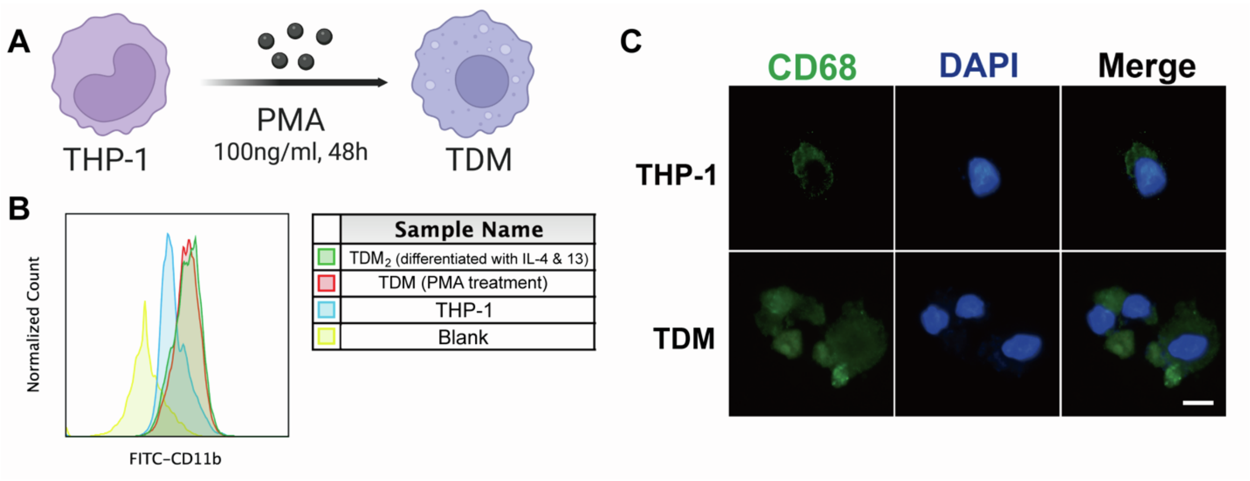

Figure S2. Acquisition of THP-1 derived macrophages, related to Figure 1 and the method detail, 'Cell culture and treatments' in STAR Methods.

(A) Schematic workflow of the acquisition of THP-1 derived macrophages. Created with BioRender.com. Flow cytometry analysis (B) and IF staining images (C) validating the success of the acquisition of TDMs. Scale bars = 10 μm.

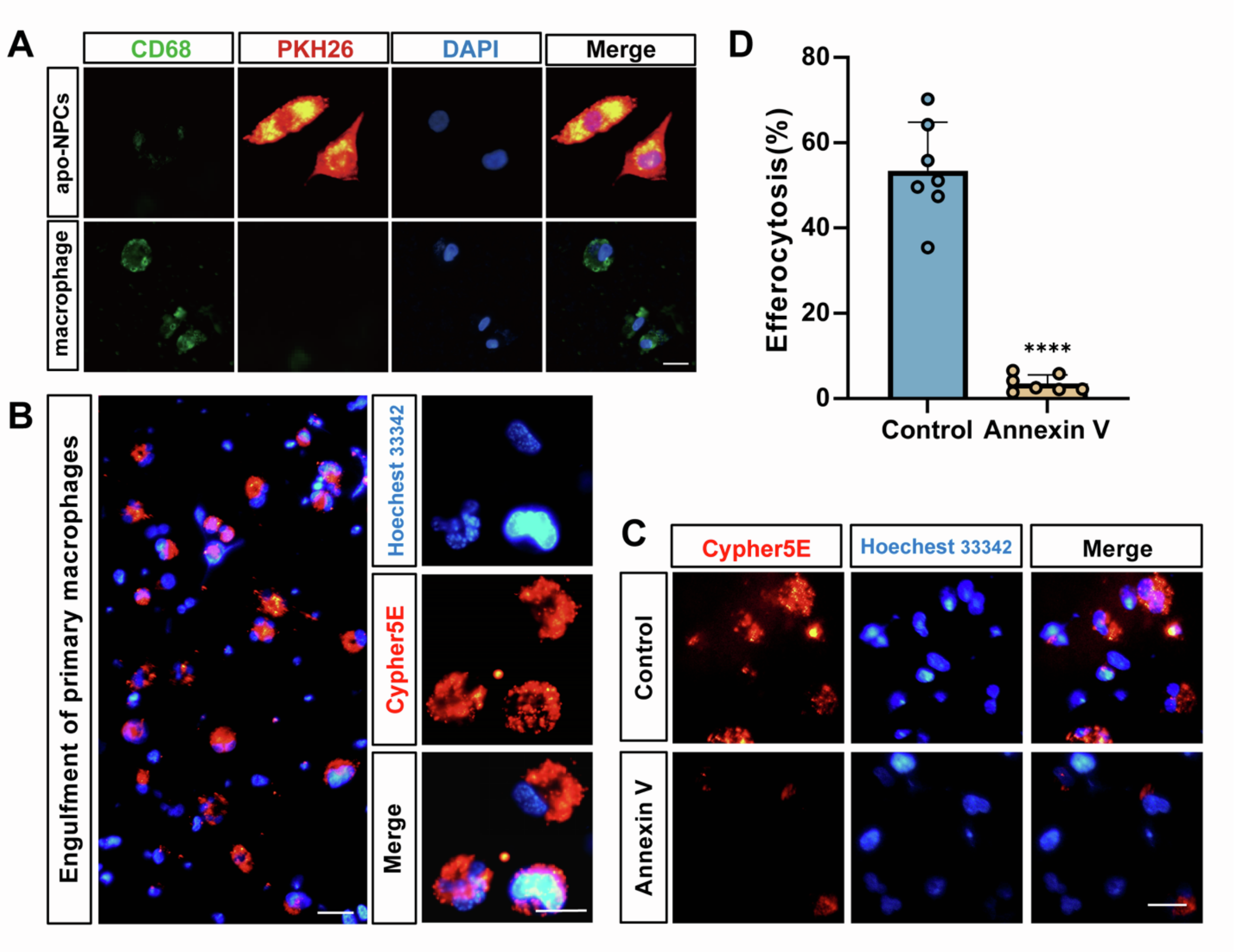

Figure S3. Efferocytosis of primary macrophages, related to Figure 1.

(A) Fluorescence image of PKH26-labeled apo-NPCs and CD68-stained macrophages beforeco-cultured. (B) Hoechest33342-labeled BMDMs co-cultured with Cypher5E-labeled apo-NPCs to show efferocytosis within the NP tissue. (C) Hoechest 33342-labeled BMDMs co-cultured with Cypher5E-labeled apo-NPCs pretreated with or without Annexin V. (D) Efferocytosis rates calculated from analyses of results shown in (C). Scale bars = 20 μm. At least 3 independent experiments were performed. Data represent means ± SD; ****$p < 0.0001$, by Student's *t* test.

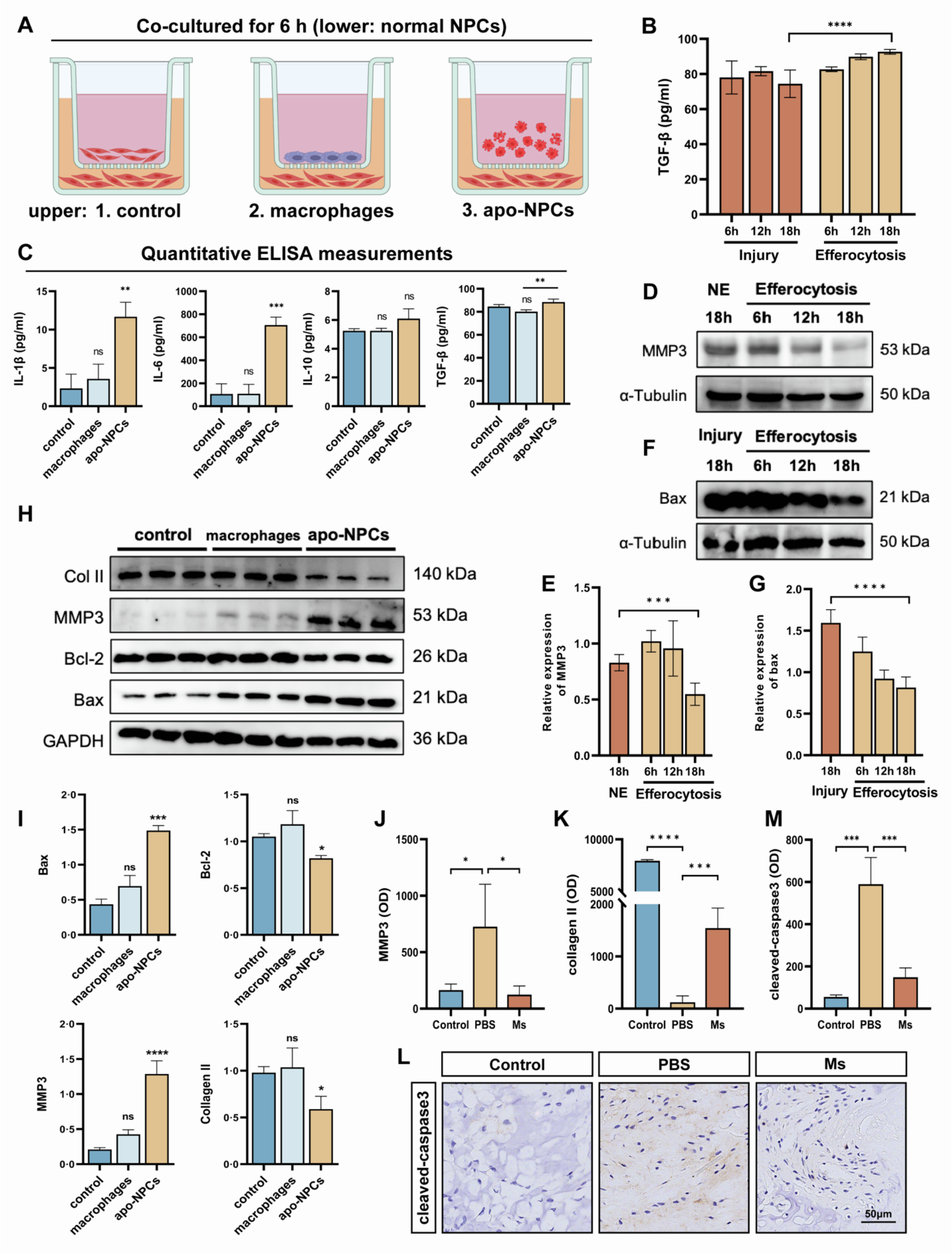

A
Co-cultured for 6 h (lower: normal NPCs)
upper: 1. control
2. macrophages
3. apo-NPCs
B
TGF-β (pg/ml)
6h 12h 18h 6h 12h 18h
Injury
Efferocytosis
C
Quantitative ELISA measurements
IL-1β (pg/ml)
IL-6 (pg/ml)
IL-10 (pg/ml)
TGF-β (pg/ml)
control
macrophages
apo-NPCs
D
NE
Efferocytosis
18h 6h 12h 18h
MMP3
53 kDa
α-Tubulin
50 kDa
F
Injury
Efferocytosis
18h 6h 12h 18h
Bax
21 kDa
α-Tubulin
50 kDa
H
control
macrophages
apo-NPCs
Col II
140 kDa
MMP3
53 kDa
Bcl-2
26 kDa
Bax
21 kDa
GAPDH
36 kDa
E
Relative expression of MMP3
18h 6h 12h 18h
NE
Efferocytosis
G
Relative expression of bax
18h 6h 12h 18h
Injury
Efferocytosis
I
Bax
Bcl-2
MMP3
Collagen II
control
macrophages
apo-NPCs
J
MMP3 (OD)
Control PBS Ms
K
collagen II (OD)
Control PBS Ms
M
cleaved-caspase3 (OD)
Control PBS Ms
L
Control
PBS
Ms
cleaved-caspase3
50μm

Figure S4. Macrophages regulate extracellular matrix synthesis and catabolism through efferocytosis, related to Figure 2.

(A) Schematic graph of the supplementary experimental workflow with the co-culture model without cell-to-cell contact. Created with BioRender.com. (B) Quantitative ELISA measurements of TGF-β in supernatants from injury and efferocytosis groups after 6, 12, 18 hours of co-culture. (C) Quantitative ELISA measurements of IL-1β, IL-6, IL-10 and TGF-β in co-culture supernatants from the supplementary experiment. (D to G) Representative western blots showing expression of MMP3 (D and E) and Bax (F and G) in human NPCs after cultured in the co-cultured model for 6, 12, 18 hours. (H and I) Representative western blots (H) and their statistical analysis results (I) showing expression of Bax, Bcl-2, MMP3, and collagen type II in human NPCs after cultured in the co-cultured model from the supplementary experiment. (J and K) Mean optical density of IHC staining of MMP3 (J) and collagen type II (K) calculated from analyses of results shown in Figure 2N and 2O. (L and M) Representative images (L) and the calculated mean optical density (M) of cleaved-caspase3 IHC staining in rat coccygeal IVDs. Scale bars are indicated separately in each group of images. At least 3 independent experiments were performed. Data represent means ± SD, *$p < 0.05$, **$p < 0.01$, ***$p < 0.001$, ****$p < 0.0001$, ns. no significance, by two-way ANOVA (C, I to K and M) and Student's *t* test (B, E and G).

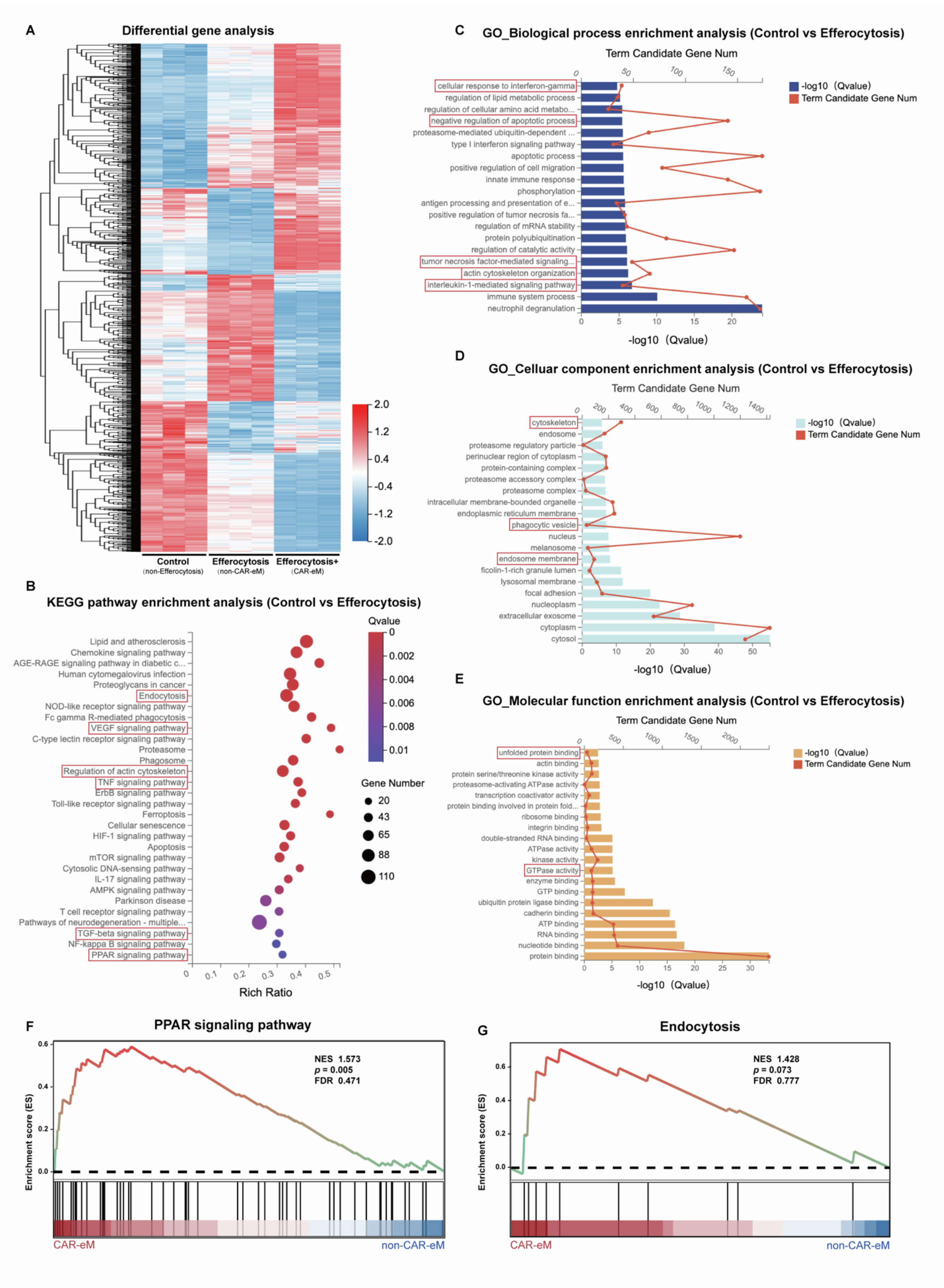
A
Differential gene analysis
2.0
1.2
0.4
-0.4
-1.2
-2.0
Control
(non-Efferocytosis)
Efferocytosis
(non-CAR-eM)
Efferocytosis+
(CAR-eM)
B
KEGG pathway enrichment analysis (Control vs Efferocytosis)
Lipid and atherosclerosis
Chemokine signaling pathway
AGE-RAGE signaling pathway in diabetic c...
Human cytomegalovirus infection
Proteoglycans in cancer
Endocytosis
NOD-like receptor signaling pathway
Fc gamma R-mediated phagocytosis
VEGF signaling pathway
C-type lectin receptor signaling pathway
Proteasome
Phagosome
Regulation of actin cytoskeleton
TNF signaling pathway
ErbB signaling pathway
Toll-like receptor signaling pathway
Ferroptosis
Cellular senescence
HIF-1 signaling pathway
Apoptosis
mTOR signaling pathway
Cytosolic DNA-sensing pathway
IL-17 signaling pathway
AMPK signaling pathway
Parkinson disease
T cell receptor signaling pathway
Pathways of neurodegeneration - multiple...
TGF-beta signaling pathway
NF-kappa B signaling pathway
PPAR signaling pathway
Rich Ratio
Qvalue
Gene Number
C
GO_Biological process enrichment analysis (Control vs Efferocytosis)
Term Candidate Gene Num
-log10 (Qvalue)
Term Candidate Gene Num
cellular response to interferon-gamma
regulation of lipid metabolic process
regulation of cellular amino acid metabo...
negative regulation of apoptotic process
proteasome-mediated ubiquitin-dependent ...
type I interferon signaling pathway
apoptotic process
positive regulation of cell migration
innate immune response
phosphorylation
antigen processing and presentation of e...
positive regulation of tumor necrosis fa...
regulation of mRNA stability
protein polyubiquitination
regulation of catalytic activity
tumor necrosis factor-mediated signaling...
actin cytoskeleton organization
interleukin-1-mediated signaling pathway
immune system process
neutrophil degranulation
-log10 (Qvalue)
D
GO_Celluar component enrichment analysis (Control vs Efferocytosis)
Term Candidate Gene Num
cytoskeleton
endosome
proteasome regulatory particle
perinuclear region of cytoplasm
protein-containing complex
proteasome accessory complex
proteasome complex
intracellular membrane-bounded organelle
endoplasmic reticulum membrane
phagocytic vesicle
nucleus
melanosome
endosome membrane
ficolin-1-rich granule lumen
lysosomal membrane
focal adhesion
nucleoplasm
extracellular exosome
cytoplasm
cytosol
-log10 (Qvalue)
E
GO_Molecular function enrichment analysis (Control vs Efferocytosis)
Term Candidate Gene Num
unfolded protein binding
actin binding
protein serine/threonine kinase activity
proteasome-activating ATPase activity
transcription coactivator activity
protein binding involved in protein fold...
ribosome binding
integrin binding
double-stranded RNA binding
ATPase activity
kinase activity
GTPase activity
enzyme binding
GTP binding
ubiquitin protein ligase binding
cadherin binding
ATP binding
RNA binding
nucleotide binding
protein binding
-log10 (Qvalue)
F
PPAR signaling pathway
NES 1.573
p = 0.005
FDR 0.471
Enrichment score (ES)
CAR-eM
non-CAR-eM
G
Endocytosis
NES 1.428
p = 0.073
FDR 0.777
Enrichment score (ES)
CAR-eM
non-CAR-eM

Figure S5. Supplementary molecular mechanisms of efferocytosis therapy, related to Figure 3.

(A) Heatmap of differential gene expression between the control (non-efferocytosis), efferocytosis (non-CAR-eM) and efferocytosis+ (CAR-eM) groups. Top 20 upregulated of KEGG pathway (B), GO_Biological process (C), GO_Cellular component (D) and GO_Molecular function(E) enrichment analysis between the control and efferocytosis groups. (F and G) GSEA showing enrichment of “PPAR signaling pathway” and “Endocytosis” in TDMs between the control and efferocytosis groups.

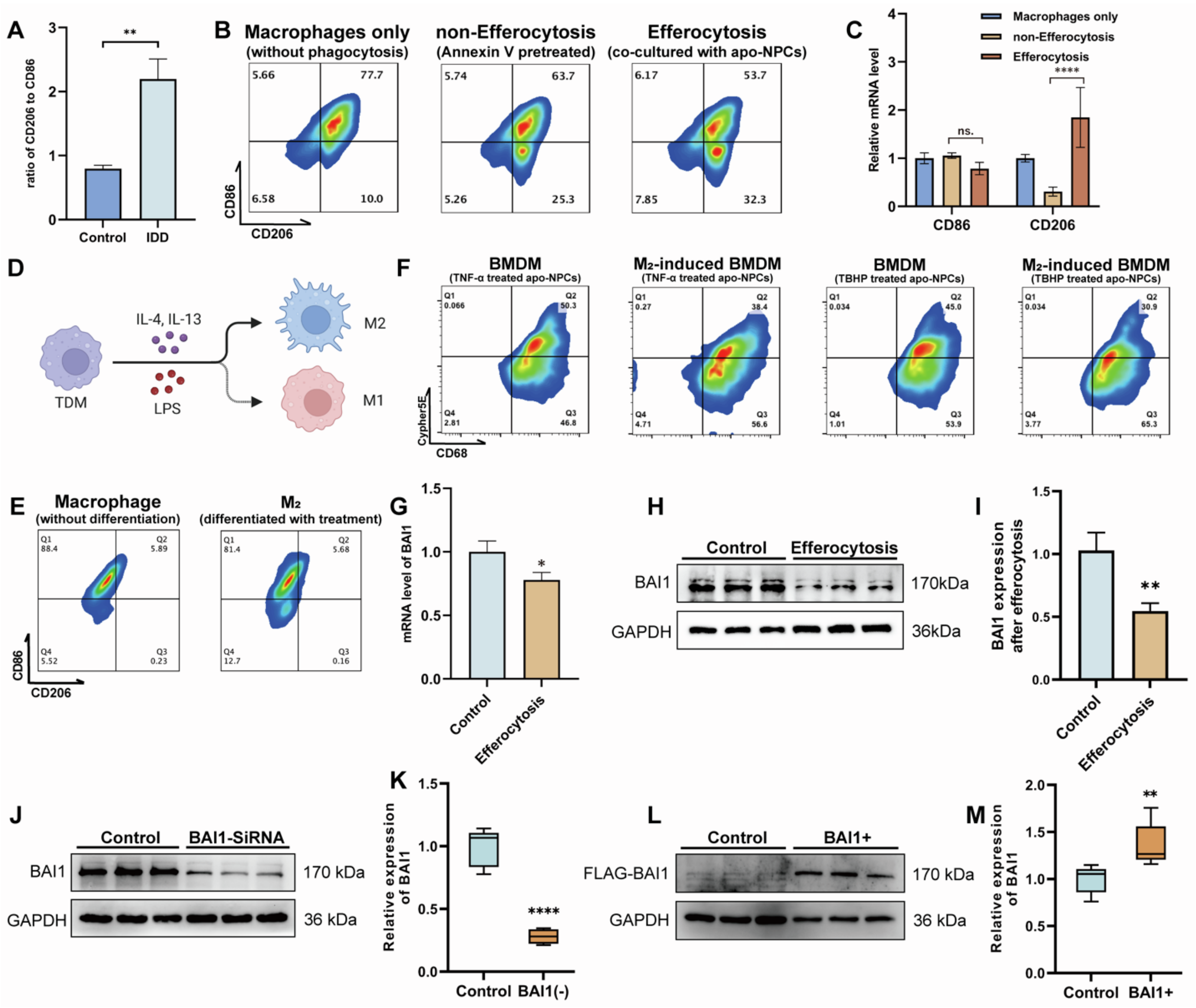

Figure S6. Phenotype transformation, decreased expression of phagocytic receptors, and reduction in phagocytic ability of macrophages after efferocytosis, related to Figure 3 and 4.

(A) Ratio of the amount of CD68+CD206+ cells to CD68+CD86+ cells calculated from analyses of results shown in Figure 4A. (B) Flow cytometry analysis of CD86 and CD206 in BMDMs of each group. (C) RT-qPCR statistical analysis showing the transcription level of CD86, CD206 in TDMs in each group. (D) Schematic workflow of acquisition of macrophage polarization. Created with BioRender.com. (E) Flow cytometry analysis validating the success of the acquisition of M_2-induced BMDMs. (F) Flow cytometry analysis of BMDMs treated with Cypher5E-labeled apo-NPCs treated with TNF-α and TBHP respectively to show the difference in efferocytosis capacity with or without M2 induction. (G) RT-qPCR statistical analysis showing the transcription level of BAI1 in control and efferocytosis groups. (H and I) Representative western blots showing the expression level of BAI1 in BMDMs in control and efferocytosis groups. (J and K) Representative western blot of BAI1 (J) and its statistical analysis (K)

showing expression changes after taking up the BAI1-siRNA. (L and M) Representative western blot of BAI1 (L) and its statistical analysis (M) showing expression changes after the upregulation treatment. At least 3 independent experiments were performed. Data represent means ± SD, *$p < 0.05$, **$p < 0.01$, ****$p < 0.0001$, ns. no significance, by Student's *t* test (A, G, I, K and M) and one-way ANOVA (C).

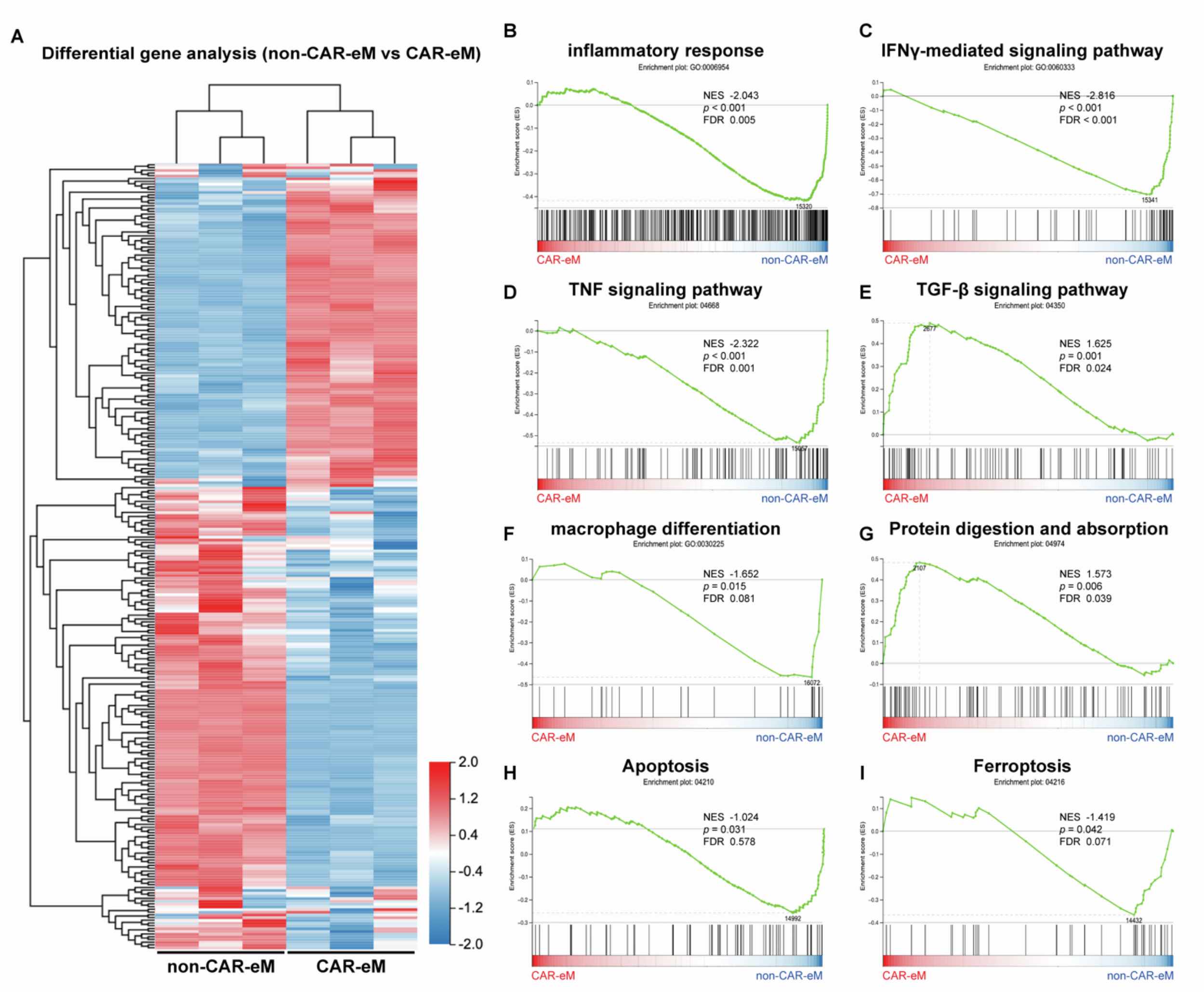

Figure S7. Supplementary molecular mechanisms of CAR-eM therapy, related to Figure 4.

(A) Heatmap of differential gene expression between the non-CAR-eM and CAR-eM groups. (B to I) GSEA showing enrichment of “inflammatory response”, “IFNγ-mediated signaling pathway”, “TNF signaling pathway”, “TGF-β signaling pathway”, “macrophage differentiation”, “Protein digestion and absorption”, “Apoptosis” and “Ferroptosis” in TDMs between the non-CAR-eM and CAR-eM groups.

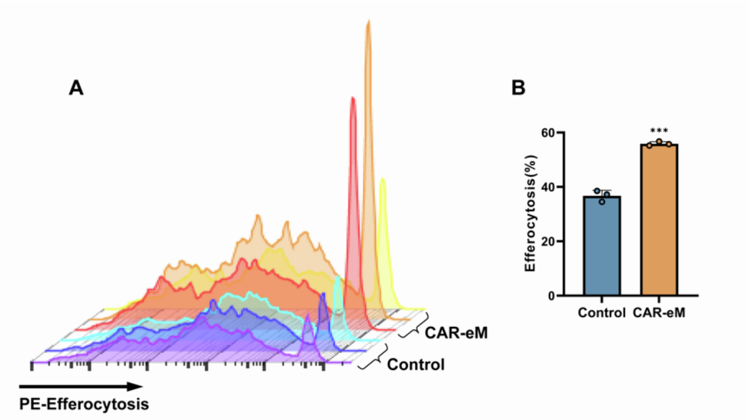

Figure S8. Efferocytosis capacity verification of CAR-eM, related to Figure 5.

(A and B) Flow cytometry analysis (A) of CAR-eMs treated with Cypher5E-labeled apo-NPCs and its calculated efferocytosis rate (B) to show the change in efferocytosis capacity after engineering. Data represent means ± SD, ***p < 0.001, by Student's *t* test. Related to Figure 6.

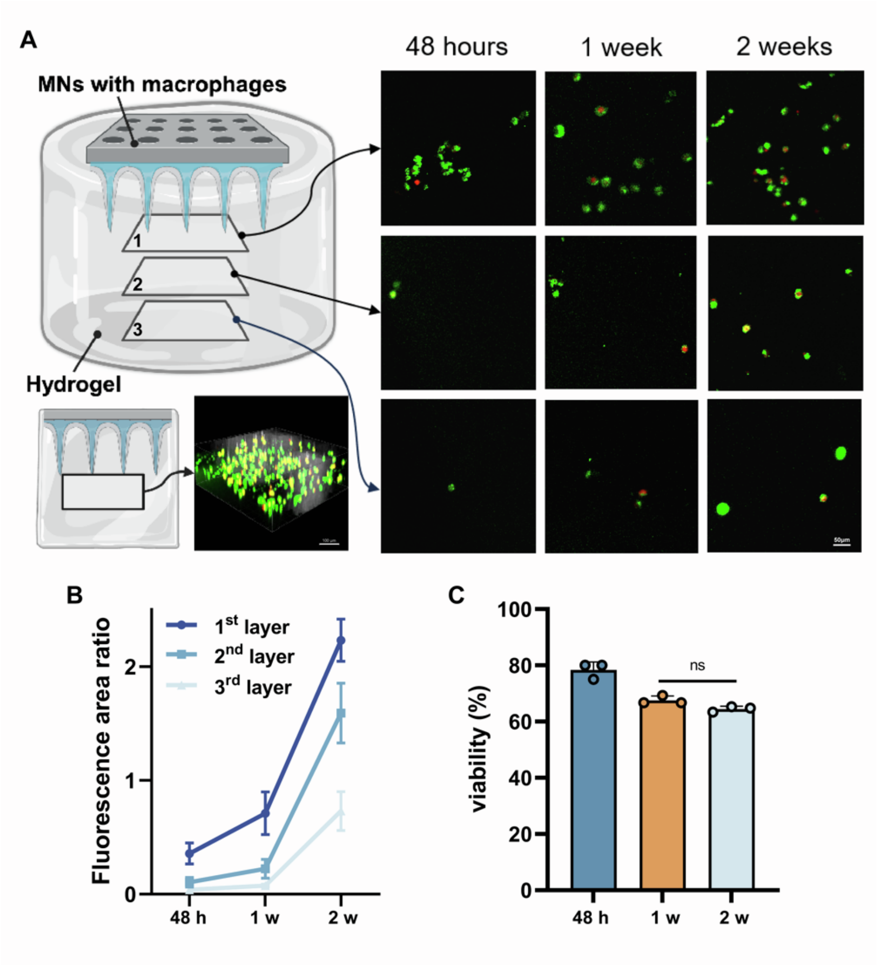

Figure S9. Release and viability of CAR-eMs from CAR-eM-MNs, related to Figure 5.

(A) Schematic graph and representative confocal microscopic images of three layers in 3D hydrogel loaded with CAR-eM-MNs, reflecting the release of CAR-eMs over different application time. (B and C) Fluorescence area ratio (B) and viability of CAR-eMs in hydrogel (C) calculated from analyses of results shown in (A). Scale bars are indicated separately in each group of images. At least 3 independent experiments were performed. Data represent means ± SD, ns. no significance, by Student's *t* test.

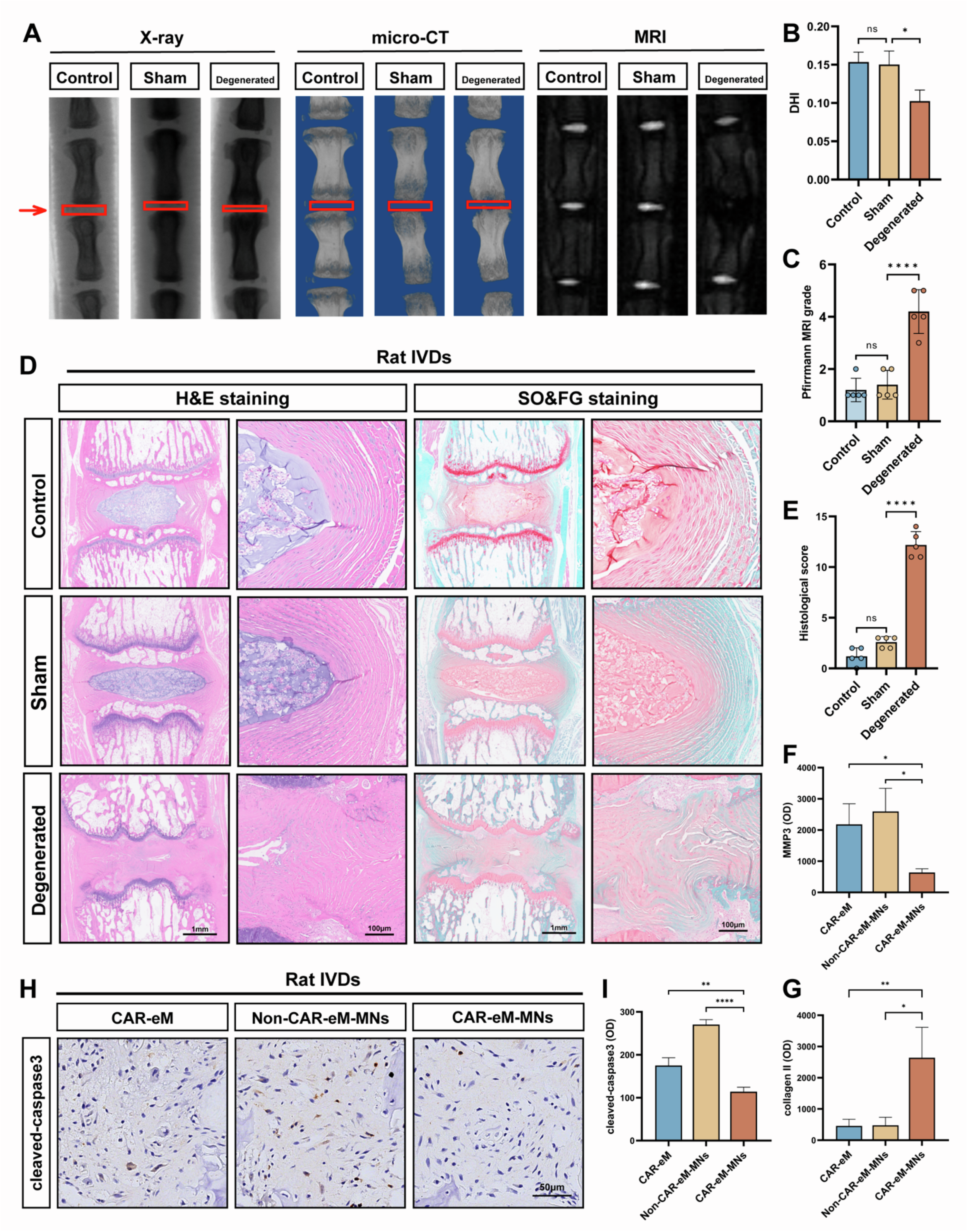

Figure S10. CAR-eM MNs applied in the in vivo treatment of IDD, related to Figure 6.

(A) Representative X-ray images, micro-CT images, and MRI of rat coccygeal IVDs from the control, sham and degenerated groups. (B and C) Disc height index (B) and Pfirrmann degenerative grades (C) of rat coccygeal IVDs in each group. (D and E) Representative H&E staining, SO&FG staining images (D) and histological scores (E) of rat coccygeal IVDs in each group. (F and G) Mean optical density of IHC staining of MMP3 (F) and collagen type II (G) calculated from analyses of results shown in Figure 7J and 7K. (H and I) Representative images (H) and the calculated mean optical density (I) of cleaved-caspase3 IHC staining in rat coccygeal IVDs in each group. Scale bars are indicated separately in each group of images. For animal experiments in this section, n = 5 biological replicates. Data represent means ± SD, *p < 0.05, **p < 0.01, ****p < 0.0001, ns. no significance, by two-way ANOVA.

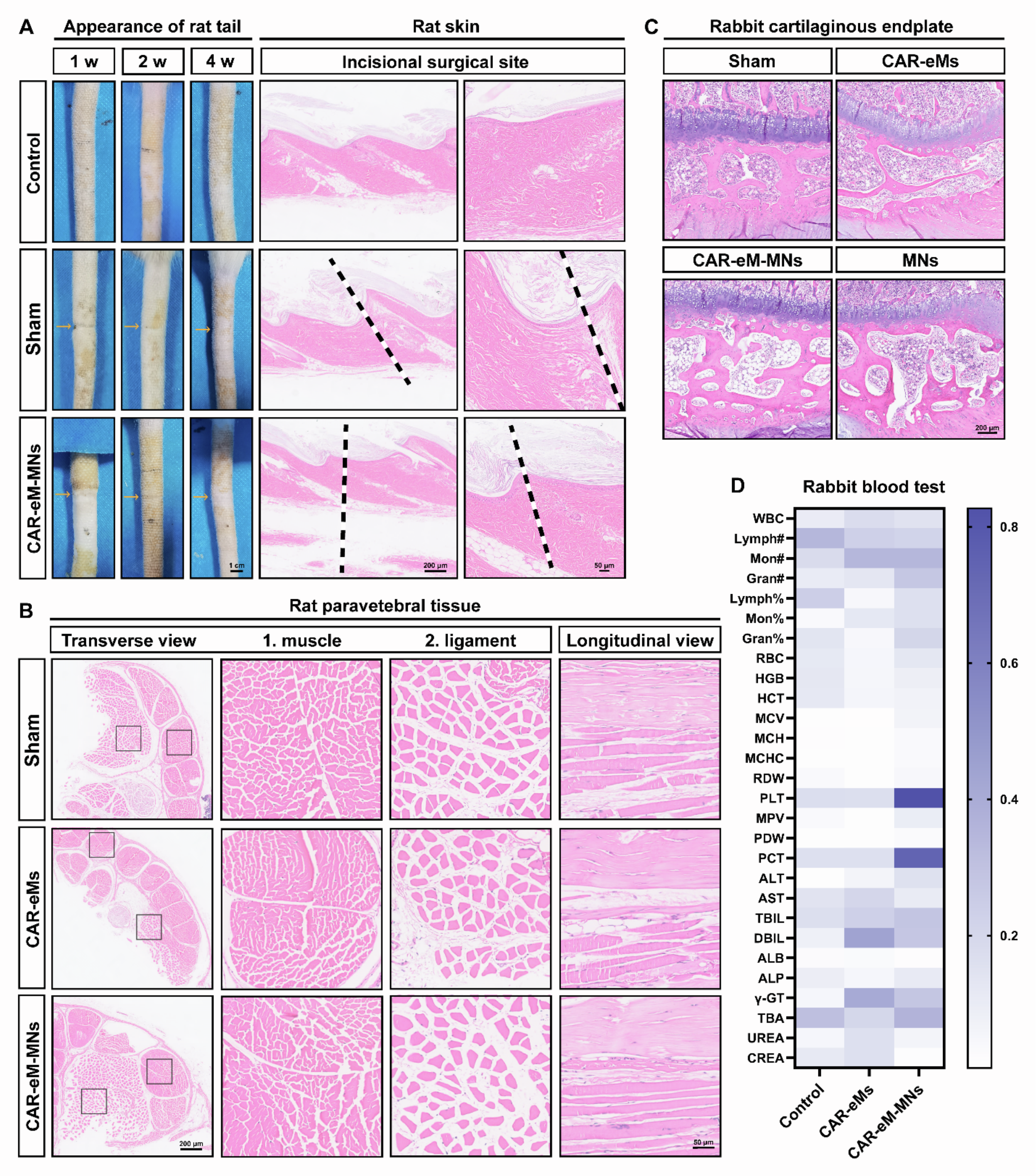

Figure S11. CAR-eM-MNs treatment didn't contribute to unexpected inflammatory response and liver and kidney function injury in vivo, related to Figure 6 and 7.

(A) Representative images of rat tails and the H&E staining of rat skin and subcutaneous tissues from different groups. The incisions are marked. (B) Representative images of the H&E staining of rat paravertebral tissues

including paravertebral muscles and paravertebral ligaments from different groups. (C) Representative images of the H&E staining of rabbit cartilaginous endplate from different groups. (D) Blood routine and biochemical tests on rabbit (n = 4 biological replicates). Scale bars are indicated separately in each group of images.

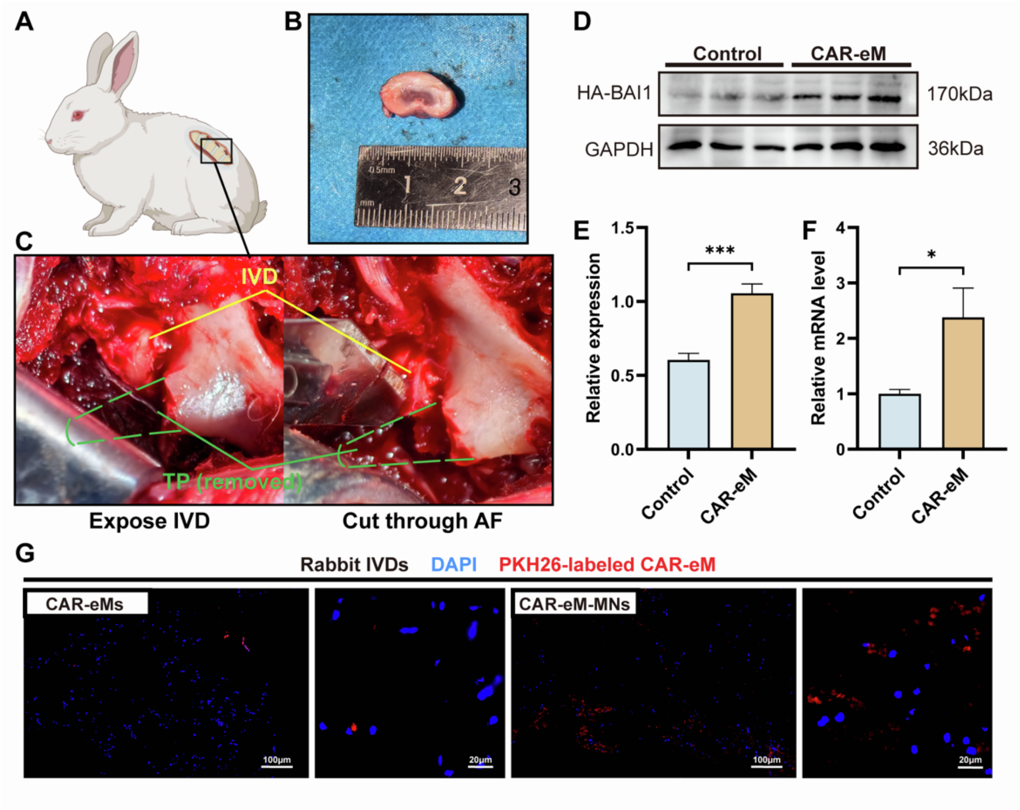

Figure S12. Therapeutic effect of CAR-eM-MNs in lumbar spine surgery, related to Figure 7.

(A) Schematic graph of retroperitoneal approach surgery for rabbits. Created with BioRender.com. (B) Images of rabbit lumbar IVD. (C) Surgical process images of exposing the third to sixth rabbit lumbar vertebrae (L3-L6) and the IVDs from corresponding segments. The positions of IVD and removed transverse process are marked. (D to F) Representative western blot (D and E) and RT-qPCR statistical analysis (F) showing BAI1 expression and its mRNA level in rabbit macrophages to verify the success of the engineered process. (G) Fluorescence images of PKH-26 labeled CAR-eMs delivered by MNs in lumbar IVDs show the long-term localization of macrophages. Scale bars are indicated separately in each image.

Table S1. Patient demographics, related to the experimental model and study participant details 'Human NP samples' in STAR Methods.

Case Number	Gender	Age (years)	Pfirrmann grade	Diagnosis	Health status	Type of consent
Case 1	Female	13	I	Idiopathic scolios	without other related diseases	Preoperative informed consent
Case 2	Male	36	II	Idiopathic scolios	without other related diseases	Preoperative informed consent
Case 3	Female	10	I	Idiopathic scolios	without other related diseases	Preoperative informed consent
Case 4	Female	15	I	Idiopathic scolios	without other related diseases	Preoperative informed consent
Case 5	Female	35	II	Idiopathic scolios	without other related diseases	Preoperative informed consent
Case 6	Male	17	I	Idiopathic scolios	without other related diseases	Preoperative informed consent
Case 7	Male	25	I	Idiopathic scolios	without other related diseases	Preoperative informed consent
Case 8	Male	13	I	Idiopathic scolios	without other related diseases	Preoperative informed consent
Case 9	Female	14	I	Idiopathic scolios	without other related diseases	Preoperative informed consent
Case 10	Female	60	IV	Lumbar disc herniation	without other related diseases	Preoperative informed consent
Case 11	Female	47	III	Lumbar disc herniation	without other related diseases	Preoperative informed consent
Case 12	Female	47	IV	Lumbar disc herniation	without other related diseases	Preoperative informed consent
Case 13	Female	25	III	Lumbar disc herniation	without other related diseases	Preoperative informed consent
Case 14	Male	34	II	Lumbar disc herniation	without other related diseases	Preoperative informed consent
Case 15	Male	53	IV	Lumbar spinal stenosis	without other related diseases	Preoperative informed consent
Case 16	Female	59	IV	Lumbar spinal stenosis	without other related diseases	Preoperative informed consent
Case 17	Female	68	IV	Lumbar spinal stenosis	without other related diseases	Preoperative informed consent
Case 18	Female	44	III	Lumbar spinal stenosis	without other related diseases	Preoperative informed consent
Case 19	Female	57	IV	Lumbar spinal stenosis	without other related diseases	Preoperative informed consent
Case 20	Male	36	III	Lumbar spinal stenosis	without other related diseases	Preoperative informed consent

Table S2. Oligonucleotide sequences used in this study, related to the method details 'RT-qPCR' and 'RNA interference and plasmid transfection' in STAR Methods.

Oligonucleotide	Sense (5'-3')	Antisense (3'-5')
**Primers for qPCR**		
Homo *IL-1β*	GGTTGAGTTTAAGCCAATCCA	TGCTGACCTAGGCTTGATGA
Homo *IL-6*	GCCCAGCTATGAACTCCTTCT	GAAGGCAGCAGGCAACAC
Homo *TGF-β*	GCAACAATTCCTGGCGATACC	CCGGTAGTGAACCCGTTGATG
Homo *IL-10*	TGCCTTCAGCAGAGTGAAGA	GCTTGGCAACCCAGGTAA
Homo *iNOS*	CGTGGAGACGGGAAAGAAGT	GACCCCAGGCAAGATTTGGA
Homo *arg-1*	TCTCAAAGGGACAGCCACGA	CGCTTGCTTTTCCCACAGAC
Homo *TNF-α*	CAGCCTCTTCTCCTTCCTGAT	GCCAGAGGGCTGATTAGAGA
Homo *CD86*	TCCCCCAGACCACATTCCTT	TCCATTGTGTTGGTTCCACATT
Homo *CD206*	TGCTACTGAACCCCCACAAC	ACCAGAGAGGAACCCATTCG
Homo *MMP3*	CAAAACATATTTCTTTGTAGAGGACAA	TTCAGCTATTTGCTTGGGAA
Homo *ADAMTS5*	TCCTGTTTACTCGGGAGGATT	GTCATGGGAGAGGCCAAGTA
Homo *Collagen II*	CCAGGACCAAAGGGACAGAA	ATCCCCTCTGGGTCCTTGTT
Homo *Aggrecan*	GCGAGCACTGTAACATAGACATT	GCCCTCCTCACATACCTCCT
Homo *BAI1*	TGCAGAACTGGACTTTGAGAAGA	CTCCTTGTCCTTCTCCGCTG
Rattus *BAI1*	TGTGCAGAACTTTGTCCAGATCA	CAAAGTCTTCCACCAGTCGAAAG
Homo *GAPDH*	CAAGAAGGTGAAGCAGG	TCAAAGGTGGAGGAGTGGGT
**siRNA sequence**		
Homo-siControl	UUCUCCGAACGUGUCACGU	ACGUGACACGUUCGGAGAA
Homo-siBAI1	CUGCACUGGUUGUGGCCAU	AUGGCCACAACCAGUGCAG

Table S3. Antibody information, related to the method details 'Western blotting analysis' and 'Flow cytometry' in STAR Methods.

Antibody	Company	Catalog#	Application/ Dilution
anti-GAPDH	Boster	BM3876	WB (1: 5000)
anti-β-actin	Boster	BM5422	WB (1: 10000)
anti-Flag tag epitope	Proteintech	66008-4-Ig	WB (1: 20000)
anti-HA tag	Proteintech	81290-1-RR	WB (1: 30000)
anti-Bcl-2	Proteintech	26593-1-AP	WB (1: 1000), IF (1: 200)
anti-Bax	Proteintech	50599-2-Ig	WB (1: 5000), IF (1: 200)
anti-Caspase3	Proteintech	19677-1-AP	WB (1: 500)
anti-MMP3	Boster	BM4074	WB (1: 2000), IHC (1: 100), IF (1: 200)
anti-ADAMTS5	Affinity	DF13268	WB (1: 500), IF (1: 200)
anti-Collagen II	Proteintech	28459-1-AP	IHC (1: 900)
anti-Collagen II	Affinity	AF0135	WB (1: 500), IF (1: 200)
anti-Aggrecan	Affinity	DF7561	WB (1: 1000), IF (1: 200)
anti-CD11b	Proteintech	66519-1-Ig	WB (1: 1000), IF (1: 500)
anti-CD68	Proteintech	66231-2-Ig	IF (1: 12000)
anti-CD86	Proteintech	26903-1-AP	WB (1: 500), IF (1: 200)
anti-CD206	Proteintech	18704-1-AP	WB (1: 500), IF (1: 200)
anti-BAI1	Abcam	Ab135907	WB (1: 100)
FITC anti-Human CD11b	Biolegend	301330	FC
BV421 anti-Human CD68	BD Pharmingen	564943	FC
PE-Cy7 anti-Human CD86	BD Pharmingen	561128	FC
APC anti-Human CD206	BD Pharmingen	550889	FC
