# Supplementary material for: Microneedle delivery of CAR-M-like engineered macrophages alleviates intervertebral disc degeneration through enhanced efferocytosis capacity
Source: Cell Rep Med. 2025 Apr 7;6(4):102079. doi: 10.1016/j.xcrm.2025.102079 (PMC12047514; doi:10.1016/j.xcrm.2025.102079)
Supplement: Document S2. Article plus supplemental information [file mmc4.pdf]

Cell Reports
Medicine

Article

Microneedle delivery of CAR-M-like engineered macrophages alleviates intervertebral disc degeneration through enhanced efferocytosis capacity

Graphical abstract

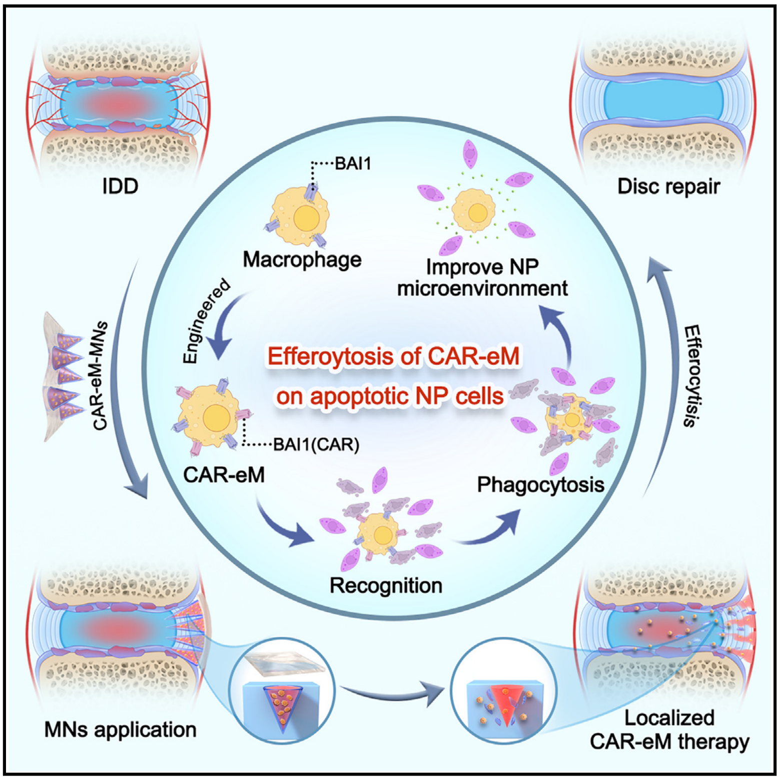

Authors

Xingyu Zhou, Dingchao Zhu, Di Wu, ..., Lei Tan, Bingjin Wang, Cao Yang

Correspondence

tanlei_hubu@163.com (L.T.),
wangbingjinly@163.com (B.W.),
caoyangunion@hust.edu.cn (C.Y.)

In brief

Zhou et al. develop a microneedle delivery system that delivers CAR-M-like engineered macrophages with enhanced efferocytosis capacity into the body, efficiently clearing apoptotic cells and improving the inflammatory microenvironment for the treatment of a highly prevalent inflammatory degenerative disorder.

Highlights

- Macrophages undergo phenotype and phagocytic ability changes after efferocytosing in IVD
- Design of CAR-M-like engineered macrophages (CAR-eMs) targeting efferocytosis
- Design of a microneedle delivery system to deliver CAR-eMs into the degenerated disc
- Microneedles loaded with CAR-eMs efficiently treat the inflammatory degenerative disorder

Zhou et al., 2025, Cell Reports Medicine 6, 102079
April 15, 2025 © 2025 The Authors. Published by Elsevier Inc.
https://doi.org/10.1016/j.xcrm.2025.102079

CellPress

Cell Reports Medicine

CellPress
OPEN ACCESS

Article

Microneedle delivery of CAR-M-like engineered macrophages alleviates intervertebral disc degeneration through enhanced efferocytosis capacity

Xingyu Zhou,[1,5] Dingchao Zhu,[1,5] Di Wu,[1,5] Gaocai Li,[1,5] Huaizhen Liang,[1] Weifeng Zhang,[1] Yali Wu,[2] Hanpeng Xu,[1] Zhengdong Zhang,[1,3] Bide Tong,[1] Yu Song,[1] Kun Wang,[1] Xiaobo Feng,[1] Jie Lei,[1] Hongchuan Wang,[1] Xiaoguang Zhang,[1] Liang Ma,[1] Yuhang Chen,[1] Junyu Wei,[1] Zixuan Ou,[1] Shuchang Peng,[1] Xinghuo Wu,[1] Lei Tan,[1,*] Bingjin Wang,[1,*] and Cao Yang[1,4,6,*]

[1]Department of Orthopaedics, Union Hospital, Tongji Medical College, Huazhong University of Science and Technology, Wuhan 430022, China

[2]Hubei Province Key Laboratory of Biological Targeted Therapy, MOE Key Laboratory of Biological Targeted Therapy, Union Hospital, Tongji Medical College, Huazhong University of Science and Technology, Wuhan 430022, China

[3]Department of Orthopedics, The First Affiliated Hospital of Chengdu Medical College, Chengdu 610500, China

[4]Shenzhen Huazhong University of Science and Technology Research Institute, Shenzhen 518057, China

[5]These authors contributed equally

[6]Lead contact

*Correspondence: tanlei_hubu@163.com (L.T.), wangbingjinly@163.com (B.W.), caoyangunion@hust.edu.cn (C.Y.)

https://doi.org/10.1016/j.xcrm.2025.102079

SUMMARY

Macrophages eliminate apoptotic cells produced daily in the body through efferocytosis. Restricted clearance can cause inflammation-related diseases. In intervertebral discs (IVDs), apoptotic nucleus pulposus cells (NPCs) are difficult to effectively remove, and their accumulation can cause changes in the inflammatory microenvironment, disrupt IVD homeostasis, and lead to IVD degeneration (IDD). Here, we present chimeric antigen receptor-M-like engineered macrophages (CAR-eMs) with enhanced efferocytosis capacity for IDD treatment. Macrophages undergo phenotypic transformation and a reduction in phagocytic ability after phagocyting apoptotic NPCs, but their efferocytosis capacity recovers with upregulated brain-specific angiogenesis inhibitor 1 (BAI1) expression. We develop a CAR-eM system with enhanced BAI1 expression and an IVD circular microneedle (MN) delivery system that utilizes arrays of MNs to deliver CAR-eMs into the deep IVD layers, thereby clearing apoptotic NPCs, ameliorating the inflammatory microenvironment, and repairing damaged IVDs. Our study explores the therapeutic potential of CAR-eM efferocytosis for IDD treatment.

INTRODUCTION

Macrophage therapy is an emerging immunotherapeutic approach that achieves therapeutic effects by injecting, transplanting, or implanting activated macrophages into patients.[1,2] Currently, it is primarily used in cancer treatment to kill tumor cells and improve the inflammatory microenvironment.[3–5] To improve the targeting of immune cells, the concept of chimeric antigen receptors (CARs) was proposed, and different CAR macrophages (CAR-Ms) were designed for macrophage therapy strategies. Some of these have entered clinical evaluation with significant effects.[1,6,7] However, few CAR-M therapy strategies have been developed for non-oncology applications. Therefore, the application of CAR-M therapy in more diseases can extend their therapeutic prospects.

The production of apoptotic cells occurs continuously in healthy adults.[8,9] These cells must be promptly cleared by phagocytic cells in the body, such as macrophages and dendritic cells, to avoid their accumulation and subsequent necrotic release of intracellular substances that can trigger inflammation.[10,11] Efferocytosis, the phagocytosis of damaged cells and intracellular substances, is crucial for maintaining the integrity of the body and the homeostatic turnover of cells.[10–12] In recent years, efferocytosis-disease relationships have been studied in various disease models such as atherosclerosis,[13,14] autoimmune system diseases,[15] chronic inflammation,[16] and cancer.[17,18]

Low back pain (LBP) is prevalent globally, affecting millions. Among spinal degenerative diseases, intervertebral disc (IVD) degeneration (IDD) is the primary cause of LBP and imposes a significant socioeconomic burden.[19–21] Previous studies have reported that IVD homeostasis disruption leads to degeneration, featuring nucleus pulposus (NP) cell (NPC) loss, extracellular matrix reduction, and increased type I collagen synthesis.[22–24] Importantly, NPC apoptosis and local accumulation of inflammatory cytokines play important roles in IDD progression and are potential targets.[25–27]

© 2025 The Authors. Published by Elsevier Inc.
This is an open access article under the CC BY license (http://creativecommons.org/licenses/by/4.0/).

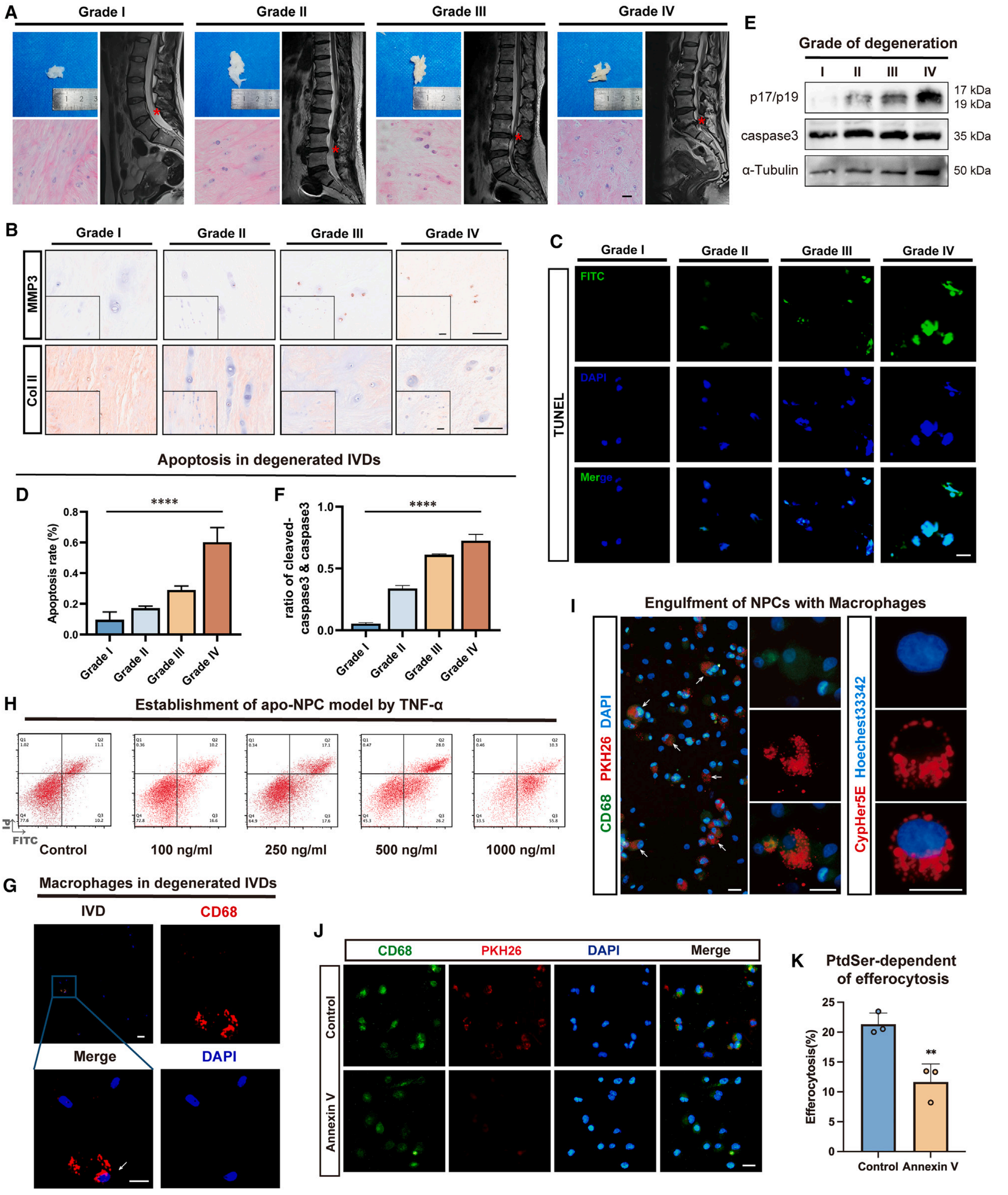

Figure 1. Macrophages infiltrate and efferocytose apoptotic NPCs in IDD

(A) Representative general views, H&E staining, and general views of human NP tissues with different degenerative grades.

(B) IHC staining of MMP3 and collagen type II in human NP tissues with different degenerative grades.

(C and D) TUNEL staining (green) (C) of human NP tissues and their statistical analysis results (D) of the percentage of TUNEL-positive cells in each grade.

(legend continued on next page)

Currently, few studies report macrophage phagocytosis in degenerated NP tissues, and it remains unknown if efferocytosis plays a role in the progression of IDD. Macrophages complete efferocytosis by recognizing phosphatidylserine (PtdSer) ligands on apoptotic cells via receptors like T cell immunoglobulin mucin (TIM)-4 and brain-specific angiogenesis inhibitor 1 (BAI1), enabling the phagocytosis of dying cells.[10,28] Engineering macrophage with a "CAR" targeting apoptotic cells to better exert its pro-phagocytic and pro-tissue repair capabilities is an intriguing and worthwhile exploration.

However, cell delivery for treatment faces unresolved issues, such as maintaining cell activity and mitigating local injection damage.[29–31] For IVDs, the damage caused by traditional acupuncture injection itself, as well as the sudden changes in the local mechanical and metabolic environment of the NP region during acupuncture injection, poses challenges to the clinical translation of cell therapy for IDD.[32,33] Microneedles (MNs) are effective drug delivery tools that consist of micron-scale needle tips and a substrate that can penetrate through a certain thickness of tissue and transport small-molecule drugs, biomolecules, and cells into deep tissues for precise therapy.[34] The advantages of MNs include smaller injuries, multiple uses, precise sustained release, and the ability to induce physical responses or stimuli. Their minimally invasive, flexible, and safe features greatly reduce dosage and adverse reactions.[2,34–36] Therefore, integrating cell therapy with MN delivery systems is a promising treatment approach.

In this study, we designed an MN delivery system to deliver CAR-M-like engineered macrophages (CAR-eMs) into the IVD to clear apoptotic NPCs (apo-NPCs), ameliorate inflammation, and delay the progression of degeneration through enhanced efferocytosis. First, we clarified the efferocytotic process in NPCs and its impact on NP tissues and explored macrophage mechanisms for treating degenerated NP tissues through efferocytosis. However, we observed that efferocytosis-induced phenotypic transformation reduced efferocytosis, linked to the downregulated phagocytic receptor BAI1. Macrophages with upregulated BAI1 exhibit a certain degree of enhanced efferocytotic ability. Therefore, we designed CAR-eMs with enhanced BAI1 expression and an IVD circular MN delivery system to deliver CAR-eMs into the deep layers of the IVD to exert therapeutic effects.

RESULTS

Macrophages infiltrate and efferocytose apo-NPCs in IDD

Previous studies have reported that IDD progression is associated with increased apo-NPCs and that immune cell infiltration also affects the immune microenvironment within the IVD.[37,38] To further investigate efferocytosis within IVDs, we collected NP tissues from volunteers undergoing surgery for idiopathic scoliosis or lumbar disc degenerative diseases. The Pfirrmann grading system, based on magnetic resonance imaging (MRI) T2-weighted hyperintense signal changes, assessed NP tissue degeneration, showing reduced moisture content and increased fibrosis during IDD progression[39] (Figure 1A). Hematoxylin and eosin (H&E) staining revealed varying degrees of disorder and calcification in the NP matrix of different degeneration grades[40] (Figure 1A). Additionally, we performed immunohistochemical (IHC) staining of these specimens and observed that as the NP tissue degeneration grade increased, the level of matrix metalloproteinase (MMP) 3 increased, whereas that of collagen type II (Col II) decreased[23] (Figure 1B), thus indicating a positive correlation between histological degeneration and imaging results. Subsequently, we used TUNEL staining to investigate apoptosis within the NP tissue during the progression of IDD[41] (Figures 1C and 1D). Degeneration of NP tissue is accompanied by an increase in the number of apoptotic cells. Western blotting (WB) showed a markedly elevated cleaved caspase-3 (p17/p19) to caspase-3 ratio[42] (Figures 1E and 1F).

Additionally, as IVD degenerates, neovascularization and neurogenesis occur, extending into the NP region, which lays the structural foundation for immune cell infiltration.[43–45] Studies have confirmed the infiltration of immune cells, including CD68$^+$ macrophages, into the IVD from the perspectives of histology and single-cell RNA sequencing.[46–48] Furthermore, we performed immunofluorescence (IF) analysis of the degenerated NP tissues and observed CD68$^+$ cells in the degenerated NP tissue, thus indicating that IDD was accompanied by macrophage infiltration (Figure 1G). These findings lay the foundation for efferocytosis of IVD.

To further study this process at the cellular level *in vitro*, we isolated human NPCs from the surgical specimens described earlier and used tumor necrosis factor alpha (TNF-α), an inflammatory cytokine involved in the pathogenesis of IDD,[25,49] to establish an apo-NPC model. Flow cytometric analysis using Annexin V/propidium iodide (PI) staining demonstrated that the proportion of apo-NPCs increased in a dose-dependent manner (Figure 1H). We selected a TNF-α concentration of 500 ng/mL for 24 h to establish an apo-NPC model and confirmed degeneration of NPCs using WB and IF analysis (Figures S1A and S1B). Additionally, we obtained THP-1-derived macrophages (TDMs) by inducing THP-1 cells and performed corresponding validation experiments[50,51] (Figures S2A–S2C). Based on these results, we co-cultured TDMs with apo-NPCs labeled with PKH26 or Cypher5E for 6 h and detected macrophage phagocytosis and corpse acidification

(E and F) Representative western blots (E) and their statistical analysis results (F) of the ratio of cleaved caspase-3 (p17/p19) to caspase-3 showing relative degree of apoptosis in human NP tissues with different degenerative grades.

(G) IF staining of CD68 (red) to verify the infiltration of macrophages in degenerated IVDs.

(H) Flow cytometry analysis using Annexin V/PI staining in human NPCs stimulated with TNF-α gradient dose of 0, 100, 250, 500, and 1,000 ng/mL for 24 h.

(I) CD68-stained (green, left) or Hoechest33342-labeled (right) TDMs co-cultured with PKH26-labeled (left) or Cypher5E-labeled (right) apo-NPCs to show efferocytosis within the NP tissue.

(J) CD68-stained TDMs co-cultured with PKH26-labeled apo-NPCs pretreated with or without Annexin V.

(K) Efferocytosis rates calculated from analyses of results shown in (J). Scale bars, 20 μm. At least 3 independent experiments were performed.

Data represent means ± SD, $^{**}p < 0.01$, $^{****}p < 0.0001$, by one-way ANOVA (D and F) and Student's t test (K).

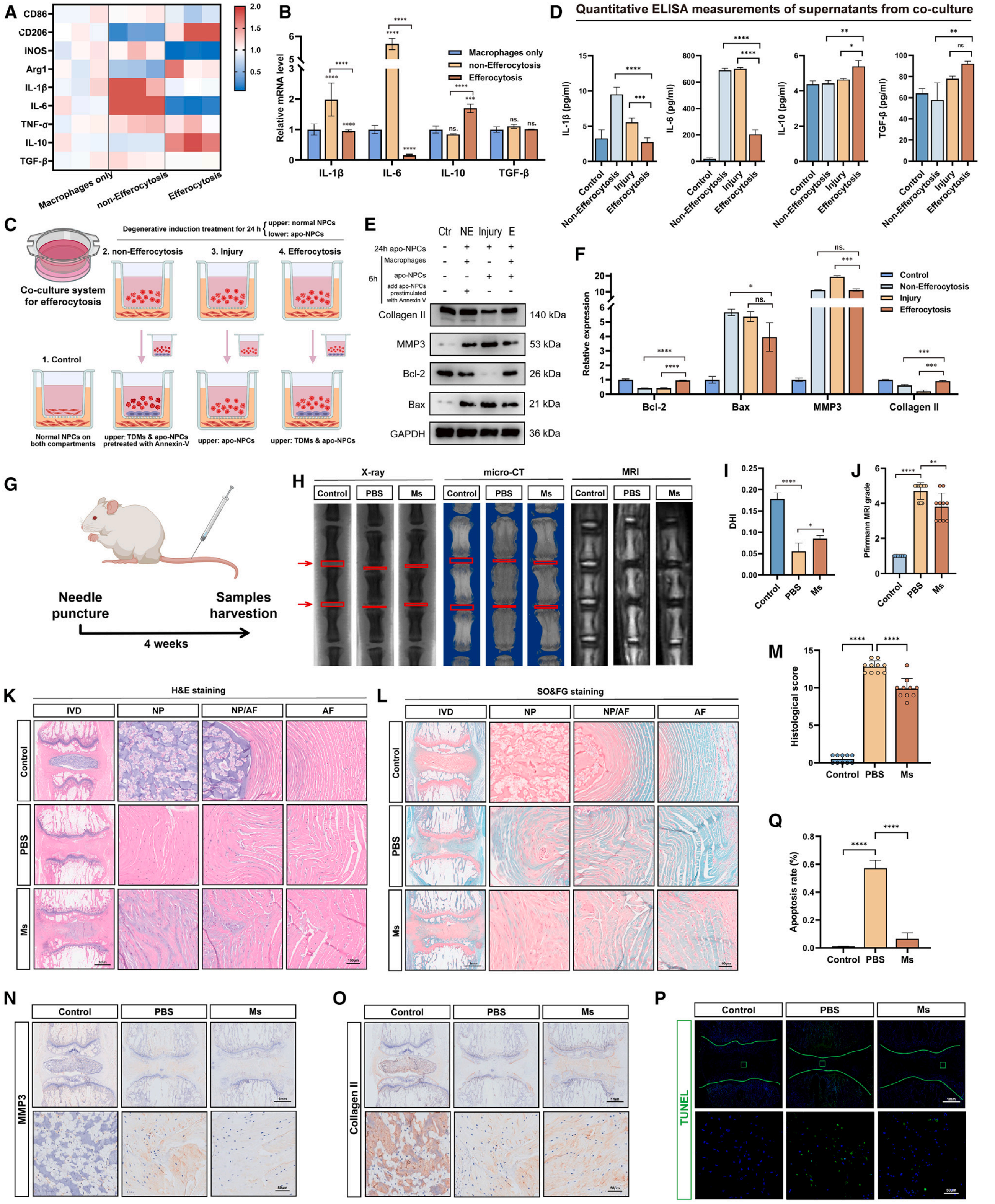

Figure 2. Macrophages regulate extracellular matrix synthesis and catabolism through efferocytosis

(A and B) Heatmap (A) and bar graph (B) of quantitative reverse-transcription PCR (RT-qPCR) statistical analysis showing the transcription levels of CD86, CD206, iNOS, Arg1, IL-1β, IL-6, TNF-α, IL-10, and TGF-β in TDMs with or without efferocytosis.

(legend continued on next page)

CellPress
OPEN ACCESS

using fluorescence microscopy (Figures 1I and S3A). The addition of Annexin V inhibited the uptake, indicating that phagocytosis was completed through the recognition of PtdSer on the surface of apo-NPCs[52] (Figures 1J and 1K). These findings were validated in primary human bone-marrow-derived macrophages (hBMDMs) (Figures S3B–S3D), revealing an interaction between macrophages and apo-NPCs via efferocytosis.

Macrophages regulate extracellular matrix synthesis and catabolism through efferocytosis

To investigate the regulatory role of macrophage efferocytosis in the surrounding NP tissues, we detected TDMs (macrophages only), TDMs incubated with apo-NPCs (efferocytosis), and TDMs incubated with Annexin V-pretreated apo-NPCs (which almost completely inhibited efferocytosis, non-efferocytosis). Quantitative polymerase chain reaction indicated that after 6 h of efferocytosis, TDMs exhibited an increased release of anti-inflammatory substances (such as interleukin [IL]-10) and a reduced release of pro-inflammatory substances (such as TNF-α and IL-1β) (Figures 2A and 2B). This indicated a phenotypic transformation from a pro-inflammatory to anti-inflammatory state in macrophages following efferocytosis. To explore the effect of efferocytosis on surrounding NP tissue, we constructed a co-culture model with indirect cell-to-cell contact[53] (Figure 2C). The experiment was divided into four groups, with the lower compartment seeded with normal NPCs. In the control group, the upper compartment was seeded with normal NPCs, whereas in the other three groups, the upper compartment was seeded with apo-NPCs, thus indicating that they had received degenerative induction treatment. After 24 h of co-culture, the upper-compartment cells were removed, and the control group received normal NPCs again. The non-efferocytosis group received TDMs co-cultured with apo-NPCs pretreated with Annexin V, the injury group received apo-NPCs, and the efferocytosis group received TDMs co-cultured with apo-NPCs. After another 6 h of culture, upper-compartment supernatants and lower-compartment NPCs were collected for detection.

Enzyme-linked immunosorbent assays (ELISAs) of the supernatants showed increased inflammatory substance production in the injury group, while changes in the non-efferocytosis and efferocytosis groups were similar to the transcript-level changes (Figure 2D). Due to the limited co-culture time, there was no significant difference in the anti-inflammatory substance content between the supernatant of the injury group and efferocytosis group. Extending the co-culture time to 12 and 18 h revealed a significant increase in transforming growth factor β (TGF-β) content in the supernatant of the efferocytosis group after co-culture for 18 h (compared to the injury group), while maintaining the overall culture time (Figure S4B). To exclude TDM-induced secretion changes, additional three groups were set up, i.e., control, macrophages, and apo-NPCs, where normal NPCs, macrophages, and apo-NPCs were added to the upper compartment (Figure S4A), respectively. After co-culturing for 6 h, ELISA was performed on the upper-compartment supernatants. Macrophages alone without efferocytosis had no significant impact on inflammatory substance secretion, whereas apo-NPCs significantly increased the secretion of pro-inflammatory substances (Figure S4C). In terms of expression levels, NPCs co-cultured with apo-NPCs exhibited heightened degeneration and apoptosis compared to the control group. However, the co-culture treatment of the efferocytosis group showed some mitigation of apo-NPC-induced degeneration (Figures 2E and 2F). Extending co-culture to 18 h revealed significant differences in Bax and MMP3 expression levels in the efferocytosis group compared to the injury and non-efferocytosis groups (Figures S4D–S4G). Similarly, WB of lower-compartment NPCs in the co-culture task mentioned in Figure S4A excluded the effects of adding TDM alone on NPCs (Figures S4H and S4I).

Subsequently, we established a disc percutaneous needle puncture animal model (rat IDD model) in Sprague-Dawley rats and treated the PBS and macrophages groups (Ms group) with PBS and TDMs, respectively (Figure 2G). After 4 weeks of treatment, X-ray and micro-computed tomography (CT) analyses revealed a significant decrease in IVD height and bone destruction in the upper and lower vertebral surfaces in the PBS group. However, disc height index (DHI) was slightly higher in the Ms group than it was in the PBS group (Figures 2H and 2I). MRI indicated that the T2-weighted signal intensity was slightly higher in the Ms group than it was in the PBS group (Figures 2H and 2J). H&E staining and safranin O/fast green (SO&FG) staining revealed that the introduction of TDMs improved the morphology and arrangement of cells as well as the structural organization within the NP tissue (Figures 2K–2M). IHC and TUNEL staining revealed that compared to injection with PBS, NP tissue treated with TDMs exhibited lower MMP3 expression and higher Col II expression along with fewer apoptotic cells (Figures 2N–2Q and S4J–S4M). These findings demonstrate that exogenous macrophages introduced into NP tissue can alleviate the damage caused by needle puncture and delay the degeneration of rat IVDs. In summary, these experiments demonstrate the potential therapeutic effects of TDMs in alleviating IVD damage and delaying degeneration.

(C) Schematic graph of the experimental workflow with the co-culture model without cell-to-cell contact. Created with BioRender.com.
(D) Quantitative ELISA measurements of IL-1β, IL-6, IL-10, and TGF-β in co-culture supernatants.
(E and F) Representative western blots (E) and their statistical analysis results (F) showing expression of Bax, Bcl-2, MMP3, and collagen type II in human NPCs after cultured in the co-cultured model.
(G) Schematic illustration of the animal experimental design.
(H) Representative X-ray images, micro-CT images, and MRI of rat coccygeal IVDs from the control, PBS, and macrophages (Ms) groups.
(I and J) Disc height index (I) and Pfirrmann degenerative grades (J) of rat coccygeal IVDs (n = 10 biological replicates).
(K–M) H&E staining (K), SO&FG staining (L), and histological scores (M) of rat coccygeal IVDs (n = 10 biological replicates).
(N and O) IHC staining of MMP3 (N) and collagen type II (O) in rat coccygeal IVDs.
(P and Q) TUNEL staining (green) (P) in rat coccygeal IVDs and their statistical analysis results (Q) of apoptosis rate in each group.
Scale bars are indicated separately in each group of images. At least 3 independent experiments were performed. Data represent means ± SD, $^{*}p < 0.05$, $^{**}p < 0.01$, $^{***}p < 0.001$, $^{****}p < 0.0001$, ns. no significance, by one-way ANOVA.

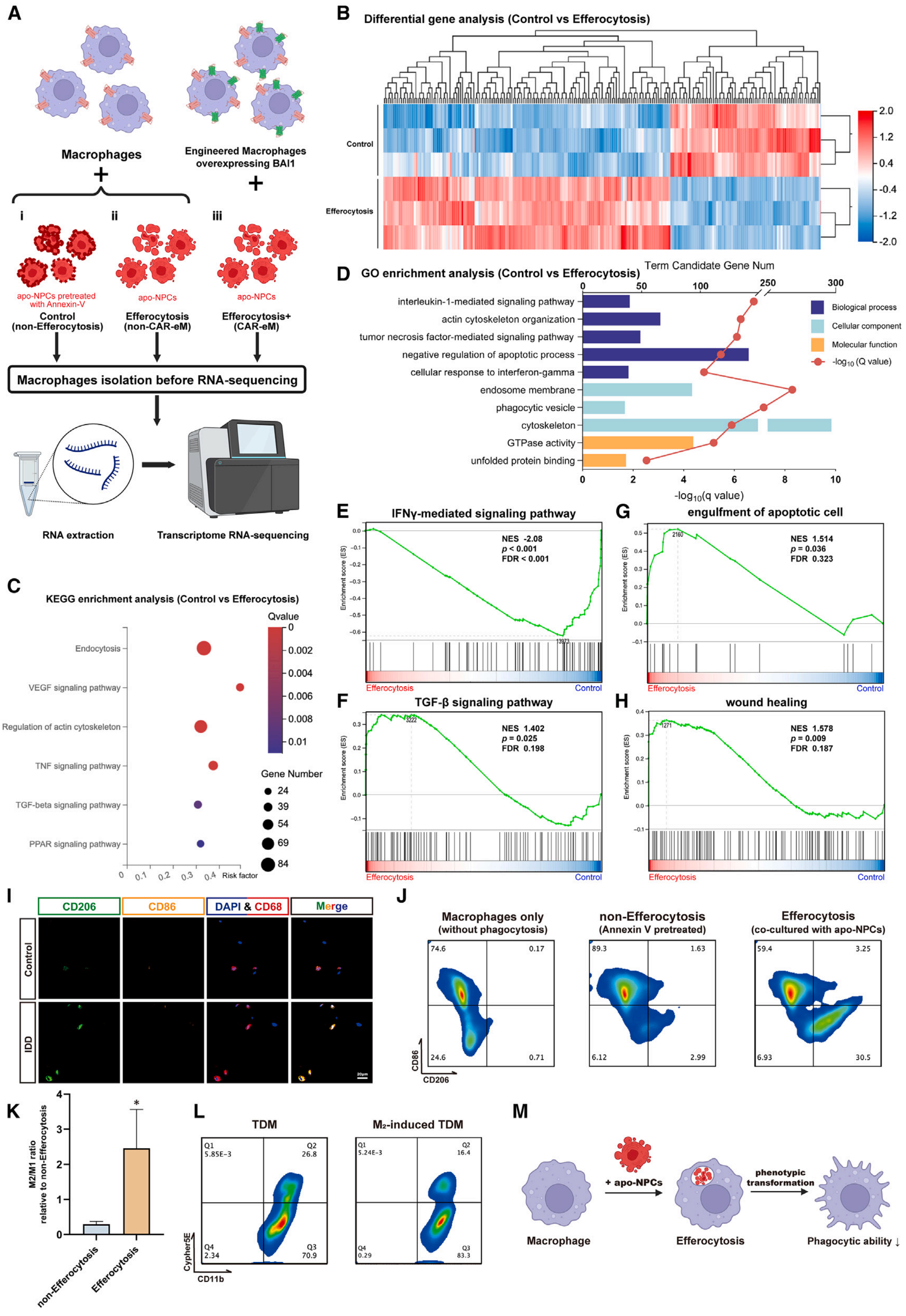

(legend on next page)

Macrophages polarize toward M2 after efferocytosis, and this is accompanied by decreased expression of phagocytic receptors and a reduction in phagocytic capacity

In this study, we explored the regulatory role of macrophages in the IVD microenvironment mediated by efferocytosis. To better understand this process, we performed RNA sequencing (RNA-seq) analysis of macrophages co-cultured with apo-NPCs (efferocytosis group) and apo-NPCs pretreated with Annexin V (control group) (Figures 3Ai and 3Aii). There was a significant difference in gene expression between the control and efferocytosis groups (Figures 3B and S5A). Kyoto Encyclopedia of Genes and Genomes (KEGG) enrichment analysis demonstrated that during efferocytosis, the inflammatory pathway and phagocytosis-related cytoskeletal changes were activated, and the transcription factor, peroxisome proliferator-activated receptor (PPAR) signaling pathway that may regulate phagocytic receptor expression during efferocytosis also exhibited significant differences compared to the control group[15] (Figures 3C and S5B). Gene ontology (GO) analysis indicated that the signaling pathways mediated by inflammatory factors and genes related to cellular inflammatory response were significantly enriched in macrophages of the efferocytosis group (Figures 3D and S5C–S5E). Additionally, gene set enrichment analysis (GSEA) demonstrated that compared to the non-efferocytosis group, macrophages undergoing efferocytosis exhibited downregulated pro-inflammatory pathways and upregulated anti-inflammatory pathways (Figures 3E and 3F), thus indicating the phenotypic transformation of macrophages after efferocytosis and confirming the previous results. Upregulation of phagocytic regulation genes aided efferocytic functions[15] (Figures 3G, S5F, and S5G). Furthermore, genes associated with "wound healing" were significantly upregulated during efferocytosis[54] (Figure 3H).

Efferocytosis regulates macrophage phenotypes, promoting pro-tissue repair effects during later stages of their action.[28,54] The tissue repair and inflammatory regulatory capabilities of macrophages are closely related to their polarization phenotypes.[10,55] In surgically excised NP tissue, IF analysis revealed a significantly increased proportion of $CD68^{+}CD206^{+}$ cells/$CD68^{+}CD86^{+}$ cells in severely degenerated NP tissue, indicating a tendency for macrophages toward M2 polarization (Figures 3I and S6A). Co-culture experiments of TDMs and BMDMs with apo-NPCs *in vitro* as demonstrated by flow cytometry indicated that efferocytosis may regulate the potential for M2 polarization (Figures 3J and S6B), and this was also verified at the transcript level (Figures 3K and S6C). To investigate the effect of macrophage phenotype on efferocytosis capacity, we induced polarization of TDMs toward the M2 phenotype[50,51] (Figures S2B, S6D, and S6E). However, polarized M2-type TDMs co-cultured with apo-NPCs exhibited a significantly lower efferocytosis rate compared to unpolarized M2-type TDMs (Figure 3L). This suggests that macrophage efferocytosis promotes the transformation toward an anti-inflammatory and pro-tissue repair phenotype, but the phagocytic ability is limited during this transformation (Figure 3M). To exclude the influence of apoptosis induction on the phenotype and phagocytic ability of macrophages, we included an additional apo-NPC model induced by tert-butyl hydroperoxide (TBHP) (100 μM)[56,57] (Figure S1C). Changes in the efferocytosis rate of hBMDM and M2-induced hBMDM co-cultured with both types of apo-NPCs were consistent with the results described earlier (Figure S6F).

The macrophage efferocytosis process comprises three stages that include the "smell phase," the "eating phase," and the "digestion phase."[10,28] The "eating phase" relies on phagocytic receptors and PtdSer for recognition and ingestion.[10,28] BAI1, a direct PtdSer-binding receptor, participates in various pathological processes and is a potential therapeutic target.[14,16,52,58] BAI1 binds to PtdSer on apoptotic cells and interacts with the cytoplasmic protein ELMO through its cytoplasmic tail domain, affecting efferocytosis efficiency.[52] To explore the decline in macrophage phagocytic ability following efferocytosis, we measured the expression of BAI1 on macrophages before and after. The results revealed that after efferocytosis, the transcriptional and translational levels of BAI1 were downregulated (Figures 4A, 4B, and S6G–S6I), and the expression level of BAI1 in polarized M2-type TDMs was lower than that in unpolarized M2-type TDMs (Figures 4C and 4D). These results suggest that in IDD, macrophage efferocytosis possesses the potential to polarize macrophages toward the M2 phenotype but is accompanied by a decrease in the expression of the phagocytic receptor BAI1 and a reduction in phagocytic ability.

Overexpression of BAI1 in CAR-eM results in enhanced efferocytosis capacity and regulatory ability of the NP tissue microenvironment

To investigate if altered BAI1 expression directly affects macrophage efferocytosis strength, we constructed a small interfering

Figure 3. Phenotype transformation, decreased expression of phagocytic receptors, and reduction in phagocytic ability of macrophages after efferocytosis

(A) Schematic workflow of the RNA-seq of TDMs from the control (non-efferocytosis, i), efferocytosis (non-CAR-eM, ii), and efferocytosis+ (CAR-eM, iii) groups. Created with BioRender.com.

(B) Heatmap of differential gene expression between the control and efferocytosis groups.

(C and D) KEGG (C) and GO (D) enrichment analysis between the control and efferocytosis groups.

(E–H) GSEA showing enrichment of "IFNγ-mediated signaling pathway," "TGF-β signaling pathway," "engulfment of apoptotic cell," and "wound healing" in TDMs between the control and efferocytosis groups.

(I) IF staining using CD68 (red), CD86 (yellow), and CD206 (green) to verify the phenotype of macrophages in IVDs. Scale bar is marked in the image.

(J) Flow cytometry analysis of CD86 and CD206 in TDMs of each group.

(K) The M_2/M_1 ratio calculated by determining the ratio of the transcription levels of CD206 to CD86 in each group to observe phenotypic changes of TDMs. Data represent means ± SD, $^{*}p < 0.05$, by Student's t test.

(L) Flow cytometry analysis of TDMs treated with Cypher5E-labeled apo-NPCs to show the difference in efferocytosis capacity with or without M_2 induction.

(M) Schematic illustration of the changes within phenotypic transformation and phagocytic ability. Created with BioRender.com. At least 3 independent experiments were performed.

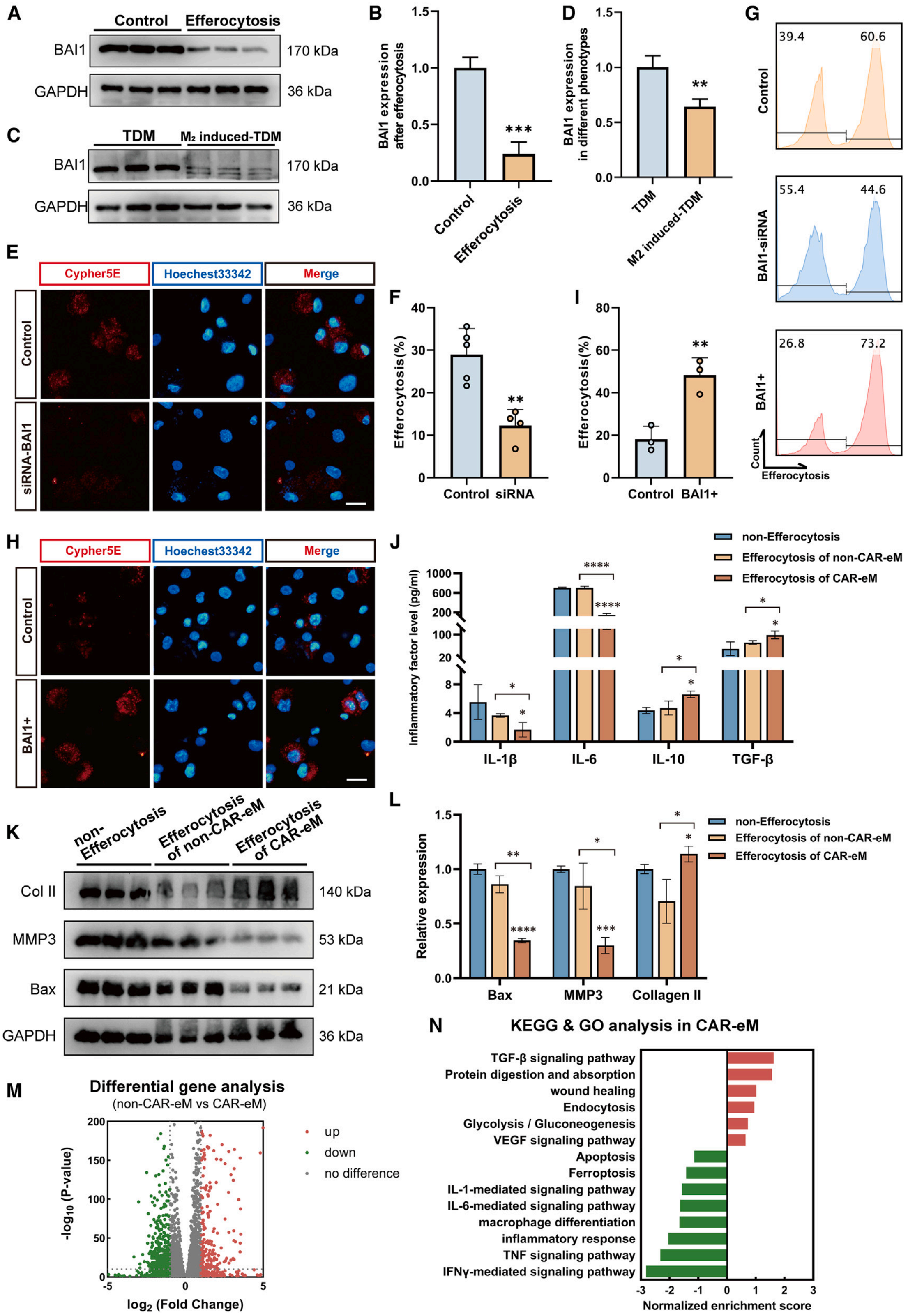

(legend on next page)

RNA (siRNA) targeting BAI1 and transfected it into TDMs. We detected a decrease in BAI1 expression on transfected TDMs (Figures S6J and S6K), and flow cytometry and IF analysis revealed that silencing of BAI1 expression led to fewer apo-NPCs phagocytosed by TDMs (Figures 4E–4G), suggesting that it limits efferocytosis. Conversely, overexpressing BAI1 in TDMs using an FLAG-tagged BAI1 plasmid (Figures S6L and S6M) increased apo-NPC phagocytosis, suggesting enhanced efferocytosis (Figures 4G–4I). These findings highlight BAI1's crucial role in regulating macrophage efferocytosis and its potential to enhance apoptotic cell recognition with increased expression.

To this end, we propose using CAR-eMs, exogenous macrophages overexpressing BAI1, with enhanced efferocytotic capacity to exert therapeutic effects on IDD. To validate this, we used a co-culture system and transfected engineered TDMs (eTDMs). The lower compartment of the co-culture system was seeded with normal NPCs and pretreated with degeneration induction as described earlier for 24 h, followed by replacement of the upper compartment with eTDMs and apo-NPC co-culture, thus simulating the introduction of exogenous CAR-eM treatment (efferocytosis of CAR-eM group). The upper layer was seeded with eTDMs, and the apo-NPCs pretreated with Annexin V co-culture served as the non-efferocytosis treatment (non-efferocytosis group) control. In addition, the upper layer was seeded with TDMs, and the apo-NPC co-culture served as the treatment control with non-engineered macrophages (efferocytosis of non-CAR-eM group). After co-culturing for 6 h, all supernatants of the NP layer were collected for ELISA detection. Compared to the non-efferocytosis group and efferocytosis of the non-CAR-eM group, the efferocytosis of CAR-eM group had a significantly altered inflammatory environment with decreased pro-inflammatory cytokines (IL-1β and IL-6) and increased anti-inflammatory cytokines (IL-10 and TGF-β) (Figure 4J). Additionally, NPCs in the lower compartment were collected, and their transcriptional and translational levels were analyzed. Results showed that NPCs in the efferocytosis of CAR-eM group exhibited fewer apoptotic tendencies and a lower degenerative phenotype (Figures 4K and 4L).

To investigate the mechanism of action of CAR-eMs in the treatment of degenerative NP tissue, we added a CAR-eM group (or efferocytosis+ group) in which eTDMs were co-cultured with apo-NPCs in previous RNA-seq (Figure 3Aiii). Compared to the non-CAR-eM group (efferocytosis group), the CAR-eM group exhibited significant transcriptome changes in macrophages (Figures 4M and S7A). According to KEGG and GO analyses, in addition to some differences in gene expression related to the inflammatory response and cytokine signaling pathways, changes in other pathways of macrophages from the CAR-eM group also confirmed the conclusions of other studies such as upregulation of genes encoding glycolysis and gluconeogenesis and downregulation of macrophage differentiation[10,11,59–61] (Figures 4N and S7B–S7F). The downregulation of cell death pathways (such as ferroptosis, apoptosis, and others) may be a protective mechanism for macrophages to clear apoptotic cells, while the upregulation of "protein digestion and absorption" genes aids in timely processing of apoptotic cell corpses (Figures S7G–S7I).

These findings suggest that introducing exogenous CAR-eMs into degenerative NP tissues possesses the potential to improve the NP tissue microenvironment, reduce NPC apoptosis, and achieve better therapeutic effects than introducing unmodified macrophages.

A plastic MN patch loaded with CAR-eM is designed for the treatment of IDD

To deliver engineered macrophages into the IVD with minimal damage and improved therapeutic effects, we designed a delivery device (CAR-eM-MNs). It consists of a poly(lactic-co-glycolic) acid (PLGA) shell and a gelatin methacryloyl (GelMA)-CAR-eM mixture, forming the needle body and a soft tape substrate (Figure 5A). PLGA provides mechanical stability,[62–64] while GelMA, crosslinkable under UV and supportive of cell viability, serves as an excellent material for cell loading,[65,66] making them both biocompatible and biodegradable.[67] To prepare this cell-loaded MN patch, we first collected peritoneal lavage fluid from rats and isolated the peritoneal macrophages (Figure 5B). Macrophages were added to the prepared GelMA solution to create a pre-polymer solution of GelMA-macrophages (Figure 5A).

The MN shells were produced by casting a mixture of PLGA and DMSO into a polydimethylsiloxane (PDMS) mold containing an array of needle cavities. The MNs possessed a height of 1,000 μm, a pitch of 650 μm, and a base diameter of 350 μm. The PLGA and DMSO mixture was cast onto the PDMS mold surface modified with O_2, and the excess material was removed by doctor blading. After drying at 70°C for 2 h to evaporate DMSO, the PLGA shell solidified (Figure 5A). To

Figure 4. Overexpression of BAI1 in CAR-eM results in enhanced efferocytosis capacity and regulation of the NP tissue microenvironment
(A and B) Western blots (A) and their statistical analysis (B) showing the transcription and expression level of BAI1 in control and efferocytosis groups.
(C and D) Representative western blots (C) and their statistical analysis (D) showing expression of BAI1 in TDMs and M_2-induced TDMs.
(E and F) Hoechest33342-labeled TDMs with or without siRNA treatment co-cultured with Cypher5E-labeled apo-NPCs. Fluorescence images (E) and their statistical analysis results (F) of the calculated efferocytosis rate to show the change in efferocytosis capacity.
(G) Flow cytometry analysis of TDMs treated with Cypher5E-labeled apo-NPCs to show the difference in efferocytosis capacity in each group.
(H and I) Hoechest33342-labeled TDMs with or without plasmid treatment co-cultured with Cypher5E-labeled apo-NPCs. Fluorescence image (H) and its statistical analysis results (I) of the calculated efferocytosis rate to show the change in efferocytosis capacity.
(J) Quantitative ELISA measurements of IL-1β, IL-6, IL-10, and TGF-β in supernatants from co-culture in each group.
(K and L) Representative western blots (K) and their statistical analysis results (L) showing expression of Bax, MMP3, and collagen type II in human NPCs in the co-cultured model.
(M) Volcano plot showing the differential gene expression between the CAR-eM and non-CAR-eM groups.
(N) KEGG and GO analysis in CAR-eM relative to non-CAR-eM. Scale bars, 20 μm. At least 3 independent experiments were performed.
Data represent means ± SD, *$p < 0.05$, **$p < 0.01$, ***$p < 0.001$, ****$p < 0.0001$, by Student's t test (B, D, F, and I) and one-way ANOVA (J and L).

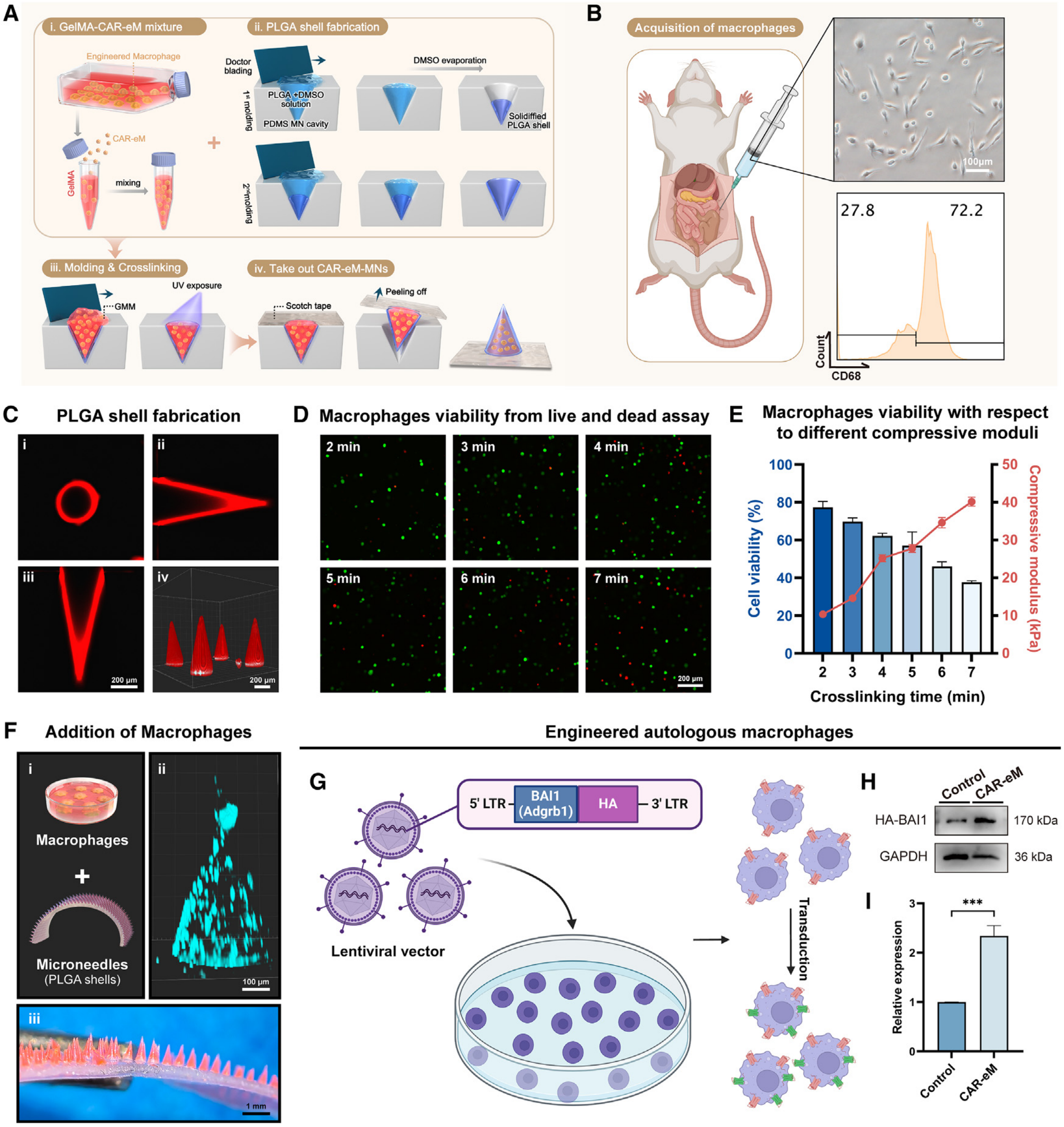

Figure 5. A plastic MN patch loaded with CAR-eM is designed for the treatment of IDD

(A) Schematic diagram of the preparation of the plastic MN patch loaded with CAR-eM.
(B) Acquisition of macrophages from rat peritoneal lavage fluid, shown by microscopic image and flow cytometry analysis.
(C) Confocal microscopic images of PLGA shell after the molding processes from three mutually perpendicular cross-sections (i, ii, and iii), and (iv) shows the three-dimensional structure.
(D) Representative images of macrophage viability from live and dead assay under different crosslinking times.
(E) Viability of 3D GelMA-macrophages with respect to different compressive moduli of GelMA tuned by crosslinking time.
(F) Addition of macrophages in PLGA shells. Schematic graph of MN fabrication (i), confocal microscopic images of Hoechest33342-labeled macrophages in MN (ii), and magnified image of the MN patch with rhodamine B-dyed PLGA shell loaded with macrophages (iii).
(G) Schematic graph of the engineering process of rat autologous macrophages. Created with BioRender.com.
(H and I) Representative western blot (H) and its statistical analysis results (I) showing BAI1 expression in rat macrophages to verify the success of the engineered process. Scale bars are marked separately on each group of images. At least 3 independent experiments were performed.
Data represent means ± SD, *** $p < 0.001$, by Student's t test.

observe the morphology of the MN shells, we added rhodamine B, a red fluorescent dye, to the mixture and repeatedly cast it to make the shells more robust and uniform. After two rounds of drying and shaping, we observed and imaged the shells using confocal fluorescence microscopy. The images indicate that the PLGA shell filled the needle cavity of the mold and exhibited a relatively uniform thickness across all confocal planes (Figure 5C).

Next, the GelMA-macrophage prepolymer solution was added to the PLGA shells and crosslinked using UV light. Considering the potential damage caused by UV exposure, we investigated the effect of crosslinking time on cell viability. Calcein-AM/PI staining revealed that the macrophage viability decreased with increasing crosslinking time (Figures 5D and 5E). At a crosslinking time of 5 min, the viability reached 64%; however, when the crosslinking time exceeded 5 min, the macrophage viability significantly decreased. The stiffness of the gel changes inversely with the crosslinking time, and both excessively soft and stiff environments are detrimental to cell survival.[67] At crosslinking times of 4–5 min, the gel stiffness ranged between 25 and 30 kPa, which was also appropriate for maintaining macrophage viability (Figure 5E). After adding the prepolymer solution of GelMA-macrophages to the PDMS mold and crosslinking for 5 min, double-sided tape was used to attach the MNs and detach them from the mold (Figures 5A and 5F). Confocal imaging of MNs containing macrophages labeled with Hoechest33342 indicated that the macrophages were evenly distributed within the conical needle body (Figure 5F).

To obtain CAR-eMs with an enhanced efferocytotic capacity, we constructed a lentiviral expression vector carrying the rat adgrb-1 (BAI1) gene. We integrated adgrb-1 into the genome of rat peritoneal macrophages using lentiviral transduction (Figure 5G). The resulting CAR-eM cells exhibited stable upregulation of BAI1 (Figures 5H and 5I), and their enhanced efferocytosis was verified by flow cytometry (Figures S8A and S8B). By using these engineered macrophages to create a plastic MN patch, we obtained CAR-eM-MNs carrying CAR-eMs. To characterize the delivery and release behavior of CAR-eMs, we introduced CAR-eM-MNs into a GelMA crosslinked hydrogel and incubated them in complete medium.[68,69] CAR-eM viability was assayed at 48-h, 1-week, and 2-week time points after the introduction of MNs (Figure S9A). Confocal imaging was performed on planes 1, 1.5, and 2 mm away from the substrate. The fluorescence area ratio of the images reflects the release of macrophages (Figures S9A and S9B), and the nutrients in culture medium and GelMA can sustain CAR-eM activity *in vitro* for at least 2 weeks (Figures S9A and S9C).

CAR-eM MNs applied in the *in vivo* treatment of IDD

We established a rat model by percutaneous needle puncture and accessed the subcutaneous tissue at the tail. Then, we separated the surface tissues of the degenerative IVD and applied CAR-eM-MNs to the IVD surface. After 1 min, the tape was removed, and the MNs were successfully transferred to the IVD tissue. This served as the CAR-eM-MN group (Figure 6A). We also used MNs loaded with primary autologous macrophages as a control for treatment with unmodified macrophages, thus forming the non-CAR-eM-MN group (Figure 6A). Additionally, CAR-eMs were injected into the rat tail IVD using needle puncture to serve as a control for the non-MN treatment, thus forming the CAR-eM group (Figure 6A). All three groups were observed for 4 weeks after treatment (Figure 6B). Prior to treatment, we labeled the isolated autologous macrophages of each group with PKH26 to detect the presence of macrophages in the IVD using *in vitro* imaging. We detected the fluorescence distribution and intensity in the tail at 0, 2, and 4 weeks after the treatment. The results revealed that after macrophage treatment, all three groups contained macrophages that were retained around the rat tail IVD in the short term. However, as the treatment time increased, PKH26 fluorescence intensity in the CAR-eM group decreased sharply. The non-CAR-eM-MN and CAR-eM-MN groups that received MN treatment maintained a certain fluorescence intensity (Figure 6C), thus indicating that the MNs provided a retention environment for macrophages during treatment. With the degradation of PLGA and GelMA, macrophages were released into the IVD to exert their therapeutic effects. The sham-operated group with only skin incision and tissue separation was compared with the untreated control group and the degeneration group with needle puncture modeling to exclude the influence of the surgery on IVDs. After 4 weeks of treatment, X-ray and micro-CT analyses suggested that CAR-eMs delivered by MNs delayed disc height loss (Figures 6C–6E, S10A, and S10B). MRI examination revealed increased T2-weighted signal intensity in the CAR-eM-MN group, thus indicating a higher IVD water content (Figures 6D, 6F, S10A, and S10C). Histologically, H&E and SO/FG staining showed preserved NP structure with good continuity of the annulus fibrosus (AF) tissue and a distinct boundary between the NP and AF regions in the CAR-eM-MN group (Figures 6G–6I, S10D, and S10E). Compared to the CAR-eM group, the AF laminar structures were better maintained, indicating that MN delivery caused less damage to the AF tissue than did conventional drug delivery. IHC and TUNEL staining assessed degenerative phenotype and apoptosis. As expected, MMP3 expression was significantly reduced in the IVDs of the CAR-eM-MN group, whereas type II collagen expression was significantly increased (Figures 6J, 6K, S10F, and S10G). The number of TUNEL-positive cells was also reduced significantly compared to that in the other groups (Figures 6L and 6M), and cleaved caspase-3 expression showed the same results (Figures S10H and S10I), indicating that macrophage efferocytosis effectively cleared a large number of apoptotic cells from degenerative IVD tissue and regulated inflammatory factor expression through phenotypic transformation to thereby improve the inflammatory microenvironment in the IVD. Finally, as observed by fluorescence imaging, macrophages delivered by MNs effectively resided in the IVD tissue, promoting long-term tissue repair (Figure 6N).

Based on the appearance of the rat tail and the morphological characteristics of the skin at the incision site and paravertebral tissues (muscles and ligaments), the surgery and implantation of MNs did not cause other safety concerns[70] (Figures S11A and S11B).

Therapeutic effect of CAR-eM-MNs in lumbar spine surgery

Surgical intervention such as lateral lumbar interbody fusion is a primary method for lumbar degenerative disease. Surgical procedures through a retroperitoneal approach preserve the bone structure of the posterior column, providing the advantages of minimally invasive surgery.[71,72]

To evaluate the potential application of MNs in lumbar spine surgery, we selected a rabbit lumbar IDD model and exposed the third to sixth lumbar vertebrae (L3–L6) and the IVDs from corresponding segments via a retroperitoneal approach,[73] as

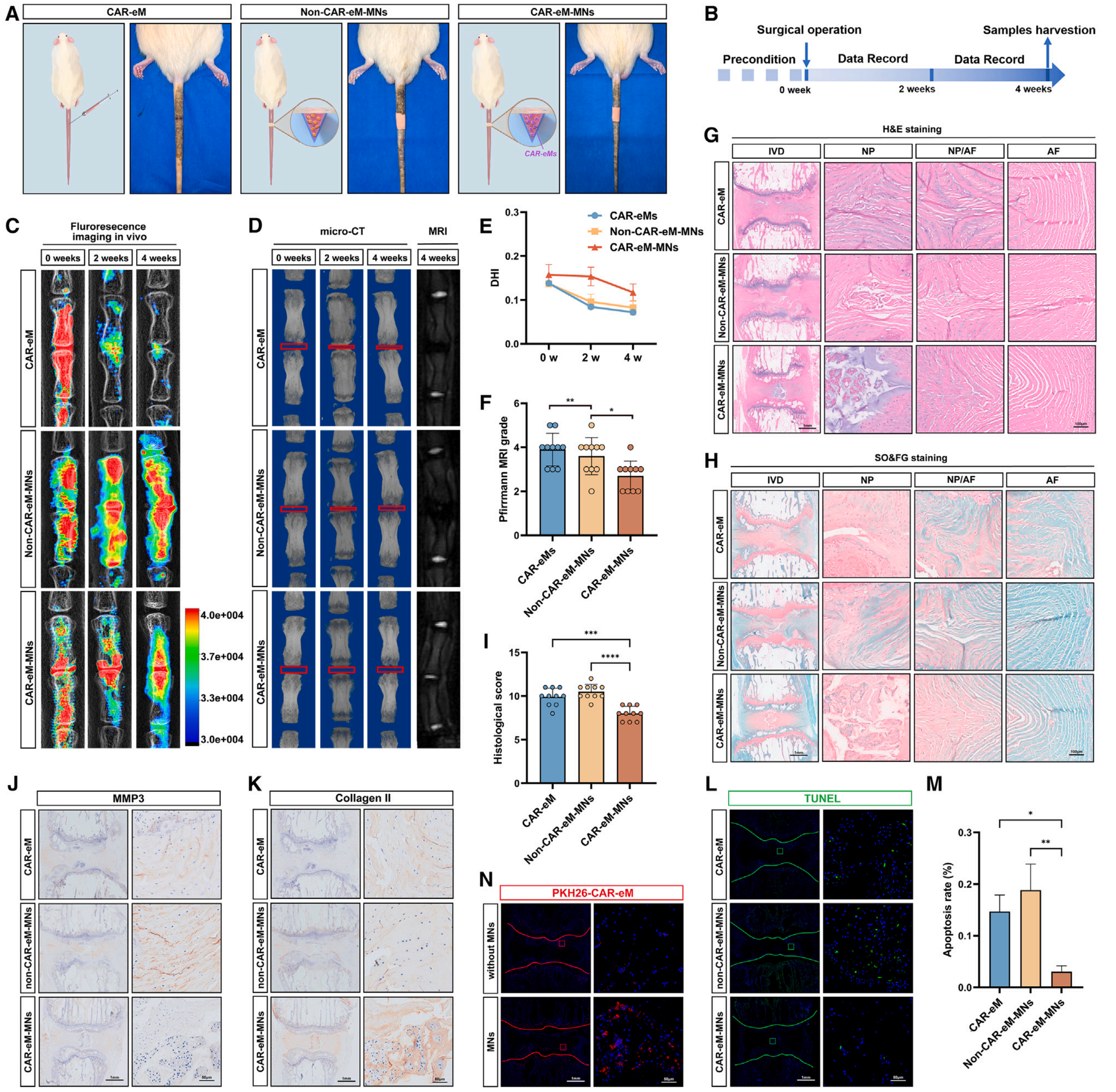

Figure 6. CAR-eM MNs applied in the *in vivo* treatment of IDD

(A) Images and schematic graphs of rat coccygeal IVDs under different treatments (CAR-eM, non-CAR-eM-MNs, and CAR-eM-MNs).

(B) Schematic workflow of the animal experimental design and different treatment methods applied in the rat coccygeal IDD model by percutaneous needle puncture.

(C) Representative X-ray images and PKH26-labeled macrophages *in vivo* show the localization of macrophages in coccygeal IVDs at different time points.

(D) Representative micro-CT images and MRI images of rat coccygeal IVDs in each group at different time points.

(E and F) Disc height index (E) and Pfirrmann degenerative grades (F) of rat coccygeal IVDs in each group.

(G–I) H&E staining (G), SO&FG staining (H), and histological score (I) of rat coccygeal IVDs in each group.

(J and K) IHC staining of MMP3 (J) and collagen type II (K) in rat coccygeal IVDs in each group.

(L and M) TUNEL staining (green) in rat coccygeal IVDs to show the apoptosis rates in each group.

(N) Fluorescence images of PKH-26-labeled CAR-eM delivered by MNs in coccygeal IVDs show the long-term localization of macrophages. Scale bars are indicated separately in each group of images. For animal experiments in this section, $n = 10$ biological replicates. Data represent means ± SD, *$p < 0.05$, **$p < 0.01$, ***$p < 0.001$, ****$p < 0.0001$, by one-way ANOVA.

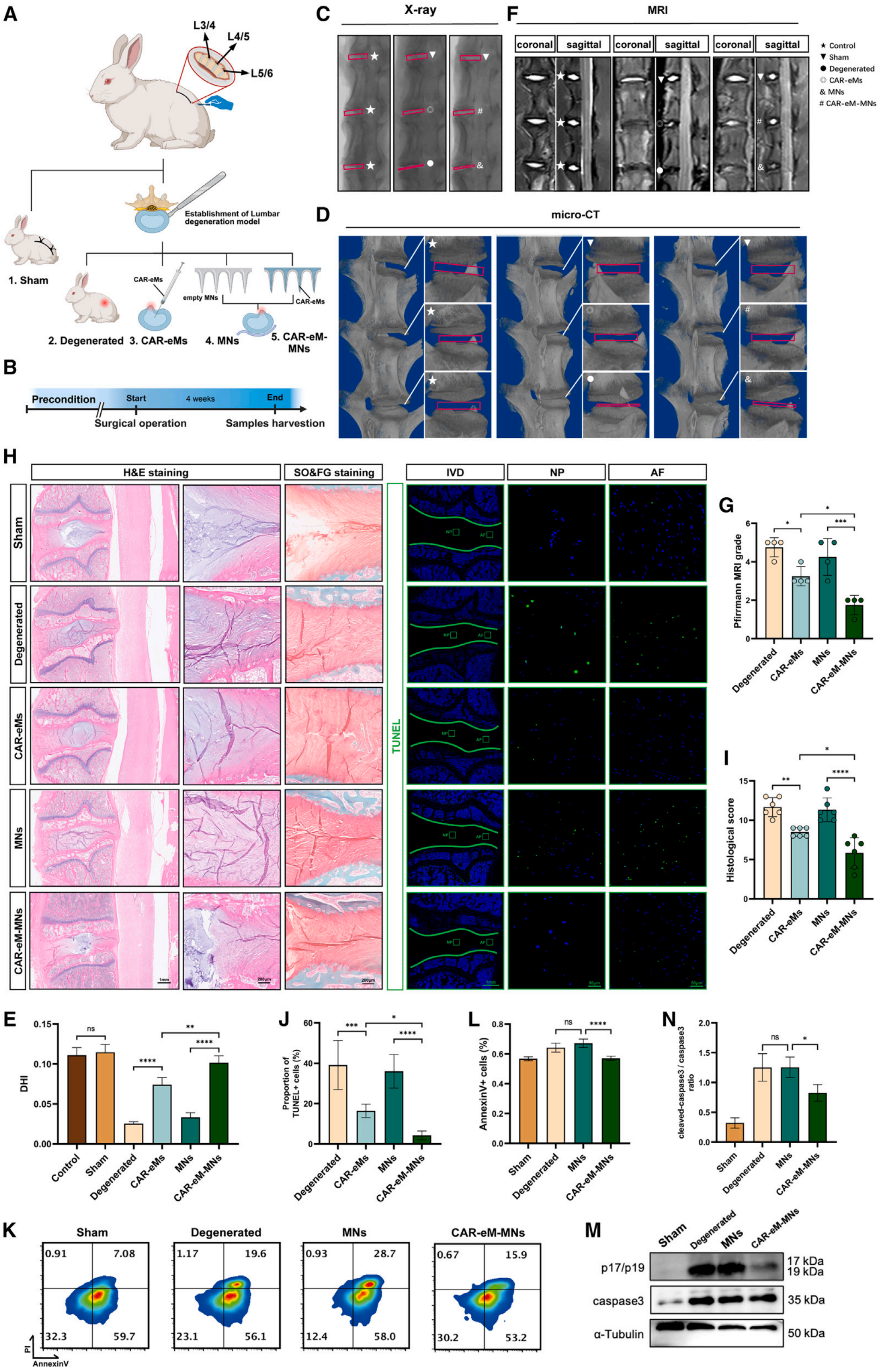

(legend on next page)

shown in Figures 7A and S12A–S12C. Subsequently, a surgical knife loading 11# blade was used to cut through the AF tissue and remove the unilateral transverse process (TP), causing lumbar instability and accelerating IDD[70] (Figure S12C). Due to the unique anatomical features of TPs in rabbits, removing them reduces the difficulty of surgical implantation of MNs. The IVDs subjected to this modeling treatment were randomly divided into the following groups: IVDs receiving CAR-eM injection through needle puncture (CAR-eMs group), IVDs receiving CAR-eM-MN treatment (CAR-eM-MNs group), IVDs implanted with MNs without loading any cells (MNs group; control), and IVDs without any additional treatments (degenerated groups). IVDs with intact AF and TP were used as the sham-operated group (sham group). The IVDs of the rabbits without surgical operation were used as the complete control group (Figures 7A and S12D–S12F). All IVD samples were collected and evaluated after 4 weeks of postoperative feeding in rabbits (Figure 7B).

The X-ray, micro-CT, and MRI images showed that the introduction of CAR-eMs reduced the degree of IVD injury after surgery and delayed the progression of degeneration, and the use of MNs improved the therapeutic effect of CAR-eM (Figures 7C–7G). H&E staining and safranin O-fast green staining showed that CAR-eM-MN application relatively better preserved IVD morphology (Figures 7H and 7I). TUNEL staining demonstrated that CAR-eM-MNs effectively reduced apoptotic cells in lumbar IVDs (Figures 7H and 7J). To better demonstrate the ability of CAR-eM to clear apoptotic cells in IVDs, we performed Annexin V/PI staining tissue flow cytometry analysis and WB-based detection of tissue cleaved caspase-3 on IVDs from the sham, degenerated, MNs, and CAR-eM-MNs groups. The results showed that in response to CAR-eM-MN treatment, the number of apoptotic cells in IVDs and the apoptotic phenotype reduced significantly, almost approaching the level in normal IVDs (Figures 7K–7N). Moreover, as evidenced through fluorescence imaging, macrophages administered via MNs successfully resided within the IVD and demonstrated sustained therapeutic efficacy in promoting tissue repair over an extended duration (Figure S12G).

Additionally, we verified that CAR-eM introduction and MN application did not contribute to unexpected inflammatory responses in the cartilaginous endplates or paravertebral tissues, including paravertebral muscles and paravertebral ligaments (Figures 7H and S11C). Blood routine and biochemical tests did not identify any unwanted immune effects and liver and kidney function injury following CAR-eM treatment[74,75] (Figure S11D).

In summary, through lumbar spine surgery, CAR-eM-MNs delivered exogenous autologous engineered macrophages into the IVD. By upregulating the expression of the phagocytic receptor BAI1 and enhancing macrophage efferocytosis, they effectively cleared apo-NPCs in the NP tissue of IDD and improved the inflammatory microenvironment in the IVD, ultimately delaying IDD progression by slowing disc degeneration.

DISCUSSION

Macrophage therapies have been demonstrated to be effective treatment strategies,[1,5,76] and the concept of CAR-M has been proposed and applied clinically to enhance targetability.[1,6,7] IDD is a pathological process characterized by the inflammatory reaction in NP tissues[38,77] causing pain and impaired daily activities.[78] Various stress reactions alter the microenvironment of IVDs, leading to apoptosis of NPCs, and apo-NPCs that cannot be cleared in time accumulate in large numbers, further exacerbating the progression of IVD.[25,79] In this study, we successfully designed an MN delivery system to deliver CAR-M-like engineered autologous macrophages (CAR-eMs) into the IVD. This delivery therapy can clear apo-NPCs, improve the inflammatory microenvironment of the IVD, repair damaged IVDs, and delay the progression of degeneration through enhanced efferocytosis.

Macrophage-mediated efferocytosis is crucial for clearing apoptotic cells and maintaining cellular homeostasis.[10,11] It also aids tissue repair by removing apoptotic cells and fragments,[54,80,81] while releasing nutrients and cytokines to support cell growth.[10,28] Our research revealed that macrophages release anti-inflammatory and tissue repair factors by phagocytizing and clearing apo-NPCs, and this improves the microenvironment of the NP tissue and restores the extracellular matrix of the IVD.

Macrophages primarily rely on phagocytic receptors such as T cell immunoglobulin mucin (TIM) family members, BAI1, stabilin-2, and the Tyro3/Axl/Mer family of tyrosine kinases that directly or indirectly bind to PtdSer and mediate efferocytosis in apoptotic cells.[10,28] BAI1 is expressed on macrophage membranes, and its extracellular domain recognizes and binds to apoptotic cells, whereas the intracellular segment interacts with ELMO1 to initiate efferocytosis-related processes.[82,83] Multiple studies have reported that the expression level of BAI1 affects efferocytosis capacity, and the effective clearance of apoptotic cells and remodeling of the tissue microenvironment are of great significance for the treatment and recovery of inflammatory diseases.[14,16,52]

Figure 7. Therapeutic effect of CAR-eM-MNs in lumbar spine surgery
(A) Schematic graphs of rabbit lumbar surgery showing experimental grouping under different treatments (sham, CAR-eMs, MNs, and CAR-eM-MNs).
(B) Schematic workflow of the rabbit experimental design.
(C–E) Representative X-ray (C), micro-CT (D), and MRI (E) images of rabbit lumbar IVDs in each group.
(F and G) Disc height index (F) and Pfirrmann degenerative grades (G) of rabbit lumbar IVDs in each group.
(H) H&E staining, SO&FG staining, and TUNEL staining (green) in rabbit lumbar IVDs in each group.
(I and J) Histological score (I) and the proportion of TUNEL+ cells (J) of rabbit lumbar IVDs in each group calculated from analyses of results shown in (H).
(K and L) Annexin V/PI staining tissue flow cytometry analysis (K) and the calculated proportion Annexin+ cells (L) of rabbit lumbar IVDs in each group.
(M and N) Representative western blots (M) and the calculated ratio of cleaved caspase-3 to caspase-3 (N) showing the apoptotic degree of rabbit lumbar IVDs in each group. For animal experiments in this section, $n > 3$ biological replicates.
Data represent means ± SD, $^{*}p < 0.05$, $^{**}p < 0.01$, $^{***}p < 0.001$, $^{****}p < 0.0001$, ns. no significance, by two-way ANOVA.

Efferocytosis is a continuous process that ensures the timely clearance of apoptotic cells in tissues. However, we observed that macrophages underwent phenotypic transformation and a reduction in phagocytic ability after efferocytosis, and this may be related to the downregulation of BAI1 expression. In our work, we used two different stimuli-induced apo-NPC models to exclude the influence of different sources of apoptosis induction on macrophage phenotype and phagocytic ability. Therefore, the phenotype transition we propose may only be one of the factors affecting efferocytosis, and perhaps efferocytosis has unique mechanisms of action on different cell types.[84] Firstly, unlike other types of cells, NPCs and their extracellular matrix contain collagens, proteoglycans, MMPs, a disintegrin and metalloproteinase with thrombospondin motifs, cathepsin proteases, some non-collagenous proteins, and other proteases,[23] which may affect macrophage activation of different signaling pathways after recognizing and engulfing apo-NPCs. Secondly, the specific cytokine milieu resulting from interactions with the apo-NPCs may further modulate macrophage responses, which is interesting and worthy of further exploration. In brief, apo-NPCs significantly affect macrophage phenotype and phagocytic ability, impacting the inflammatory response in IVD health and disease. Understanding these dynamics is crucial for developing targeted therapies aimed at modulating macrophage function in disc degeneration and associated conditions.

We investigated the role of BAI1 in mediating macrophage efferocytosis to apo-NPCs and proposed the design of an engineered macrophage with BAI1 as the "CAR" (CAR-eM) for the treatment of IDD. These results support the therapeutic potential of CAR-eMs. The upregulation of BAI1 expression in CAR-eM cells significantly improved the clearance efficiency of apo-NPCs. CAR-eMs not only exhibit increased phagocytic activity but also release higher levels of anti-inflammatory cytokines and tissue repair factors. This suggests that CAR-eMs exhibit strong therapeutic effects for the treatment of IDD.

IVD discectomy is a commonly used surgical treatment in clinical practice[85–87]; however, the procedure results in high recurrence and surgical revision rates due to the failure to repair AF defects.[88] Local injection of biologically active molecules is a common local treatment approach; however, secondary injury caused by the injection must also be considered.[68,89,90] In contrast, minimally invasive, flexible, and safe MN delivery systems exhibit broad prospects and research value for the treatment of IDD.[2,34] We designed an IVD circular MN delivery system with a soft substrate, CAR-eM-MNs, for the *in vivo* delivery of CAR-eMs. This delivery method reduces damage to the AF tissue and helps to maintain the structure of the IVDs and mechanical integrity of the spine, thereby reducing the risk of postoperative segmental instability. Its sustained-release characteristics reduce the required dosage, enhance therapeutic efficacy, and greatly improve treatment safety, thus making it a promising emerging treatment modality for IDD with significant potential.

In conclusion, our study provides evidence that CAR-eMs can effectively clear apo-NPCs and improve the inflammatory microenvironment of IVD through enhanced efferocytosis. The design of the CAR-eM-MN delivery system allows for targeted delivery of CAR-eMs to the deep layers of the IVD for therapeutic effects. However, further studies are required to confirm the therapeutic effect of CAR-eMs in larger animal models and to explore their long-term retention and survival rates *in vivo*.

Limitations of the study

Our study possesses some limitations. First, we validated the impact of BAI1 expression on macrophage efferocytosis at the cellular level using siRNA and overexpression plasmids. Employing BAI1 knockout animal models would be better for *in vivo* investigation. Large animals like goats or primates are more conducive to assessing clinical transformation potential. Furthermore, due to the spine's deep location *in vivo*, low-intensity fluorescent signals limit *in vitro* imaging observations. Methods such as isotope labeling are more favorable for macrophage tracing observations. Although our MN delivery system successfully delivered CAR-eMs into the deep layers of the IVD, the long-term retention and survival rates of these cells require further investigation. Lastly, the specific mechanisms of CAR-eM-mediated IVD repair require further exploration through efferocytosis-related intracellular signaling pathways.

RESOURCE AVAILABILITY

Lead contact

Further information and requests for resources and reagents should be directed to and will be fulfilled by the lead contact, Prof. C. Yang (caoyangunion@hust.edu.cn).

Materials availability

This study did not generate new unique reagents.

Data and code availability

- The RNA-seq data have been deposited in the Mendeley Data and are publicly accessible as of the publication date. The web address is provided in the key resources table for easy reference.
- This study did not involve the reporting of novel or original code.
- All data essential for evaluating the conclusions drawn in this paper are presented within the paper itself and/or in the supplemental information.
- Any additional information required to reanalyze the data reported in this work paper is available from the lead contact upon request.

ACKNOWLEDGMENTS

This work was supported by the National Natural Science Foundation of China (NSFC) (no. 82130072, 82072505, 82202725, 22175058, 82372380, and 82402871), the Natural Science Foundation of Hubei Province (no. 2023AFB805 and 2023AFB770), the Natural Science Foundation of Hubei Province Innovative Group Project (no. 2021CFA007), Shenzhen Science and Technology Program (SGDX20230116093544006), and the Fundamental Research Funds for the Central Universities (HUST. YCJJ20242118). We thank the Huazhong University of Science and Technology Analytical & Testing Center, Medical sub-center and Wuhan Center for Magnetic Resonance, Innovation Academy for Precision Measurement Science and Technology, Chinese Academy of Sciences, for technical support. The animal experiments in this work were also supported by Huazhong University of Science and Technology laboratory animal center. We thank the BioRender.com for providing some illustration in this manuscript.

AUTHOR CONTRIBUTIONS

C.Y., X. Zhou, G.L., B.W., and L.T. designed the experiments. X. Zhou, D.Z., and D.W. performed most of the experiments and analyzed the data with the assistance of H.L., W.Z., and Y.W. L.T., J.L., H.W., X. Zhang, L.M., and Y.C. assisted in the synthesis of materials. G.L., H.X., Z.Z., B.T., J.W., Z.O., and S.P. helped to perform the animal surgery. B.W., Y.S., K.W., X.F., and X.W.

collected the clinical specimens. X. Zhou, B.W., and C.Y. wrote this manuscript.

DECLARATION OF INTERESTS

The authors declare no completing interests.

STAR★METHODS

Detailed methods are provided in the online version of this paper and include the following:

- KEY RESOURCES TABLE
- EXPERIMENTAL MODEL AND STUDY PARTICIPANT DETAILS
  - Human NP samples
  - Animals
- METHOD DETAILS
  - Cell culture and treatments
  - Isolation of primary macrophages
  - Flow cytometry
  - Reverse transcription quantitative polymerase chain reaction (RT-qPCR)
  - Western blotting analysis
  - Immunofluorescence analysis
  - RNA interference and plasmid transfection
  - Synthesis of GelMA-macrophages
  - Fabrication of PLGA shells
  - Rat coccygeal IVDs needle puncture IDD model
  - Surgical procedure of rabbit lumbar IDD model
  - *In vivo* imaging of macrophages
  - Radiological analysis
  - Histological analysis
  - Immunohistochemistry (IHC) staining
- QUANTIFICATION AND STATISTIC ANALYSIS
  - Apoptosis assays
  - Enzyme-linked immunosorbent assays (ELISA)
  - Efferocytosis assays
  - The analysis of RNA sequencing data and the examination of gene sets using gene set enrichment analysis (GSEA)
  - Mechanical testing of GelMA-macrophages
  - Macrophage viability test
  - Statistical analysis

SUPPLEMENTAL INFORMATION

Supplemental information can be found online at https://doi.org/10.1016/j.xcrm.2025.102079.

Received: March 19, 2024
Revised: October 9, 2024
Accepted: March 18, 2025
Published: April 7, 2025

REFERENCES

1. Mantovani, A., Allavena, P., Marchesi, F., and Garlanda, C. (2022). Macrophages as tools and targets in cancer therapy. Nat. Rev. Drug Discov. *21*, 799–820. https://doi.org/10.1038/s41573-022-00520-5.
2. Chang, H., Chew, S.W.T., Zheng, M., Lio, D.C.S., Wiraja, C., Mei, Y., Ning, X., Cui, M., Than, A., Shi, P., et al. (2021). Cryomicroneedles for transdermal cell delivery. Nat. Biomed. Eng. *5*, 1008–1018. https://doi.org/10.1038/s41551-021-00720-1.
3. Mantovani, A., Marchesi, F., Malesci, A., Laghi, L., and Allavena, P. (2017). Tumour-associated macrophages as treatment targets in oncology. Nat. Rev. Clin. Oncol. *14*, 399–416. https://doi.org/10.1038/nrclinonc.2016.217.
4. Sly, L.M., and McKay, D.M. (2022). Macrophage immunotherapy: overcoming impediments to realize promise. Trends Immunol. *43*, 959–968. https://doi.org/10.1016/j.it.2022.10.002.
5. Morrison, C. (2016). Immuno-oncologists eye up macrophage targets. Nat. Rev. Drug Discov. *15*, 373–374. https://doi.org/10.1038/nrd.2016.111.
6. Zhang, L., Tian, L., Dai, X., Yu, H., Wang, J., Lei, A., Zhu, M., Xu, J., Zhao, W., Zhu, Y., et al. (2020). Pluripotent stem cell-derived CAR-macrophage cells with antigen-dependent anti-cancer cell functions. J. Hematol. Oncol. *13*, 153. https://doi.org/10.1186/s13045-020-00983-2.
7. Klichinsky, M., Ruella, M., Shestova, O., Lu, X.M., Best, A., Zeeman, M., Schmierer, M., Gabrusiewicz, K., Anderson, N.R., Petty, N.E., et al. (2020). Human chimeric antigen receptor macrophages for cancer immunotherapy. Nat. Biotechnol. *38*, 947–953. https://doi.org/10.1038/s41587-020-0462-y.
8. Arandjelovic, S., and Ravichandran, K.S. (2015). Phagocytosis of apoptotic cells in homeostasis. Nat. Immunol. *16*, 907–917. https://doi.org/10.1038/ni.3253.
9. Elliott, M.R., and Ravichandran, K.S. (2016). The Dynamics of Apoptotic Cell Clearance. Dev. Cell *38*, 147–160. https://doi.org/10.1016/j.devcel.2016.06.029.
10. Morioka, S., Maueröder, C., and Ravichandran, K.S. (2019). Living on the Edge: Efferocytosis at the Interface of Homeostasis and Pathology. Immunity *50*, 1149–1162. https://doi.org/10.1016/j.immuni.2019.04.018.
11. Doran, A.C., Yurdagul, A., and Tabas, I. (2020). Efferocytosis in health and disease. Nat. Rev. Immunol. *20*, 254–267. https://doi.org/10.1038/s41577-019-0240-6.
12. Henson, P.M. (2017). Cell Removal: Efferocytosis. Annu. Rev. Cell Dev. Biol. *33*, 127–144. https://doi.org/10.1146/annurev-cellbio-111315-125315.
13. Yurdagul, A., Subramanian, M., Wang, X., Crown, S.B., Ilkayeva, O.R., Darville, L., Kolluru, G.K., Rymond, C.C., Gerlach, B.D., Zheng, Z., et al. (2020). Macrophage Metabolism of Apoptotic Cell-Derived Arginine Promotes Continual Efferocytosis and Resolution of Injury. Cell Metab. *31*, 518–533.e10. https://doi.org/10.1016/j.cmet.2020.01.001.
14. Fond, A.M., Lee, C.S., Schulman, I.G., Kiss, R.S., and Ravichandran, K.S. (2015). Apoptotic cells trigger a membrane-initiated pathway to increase ABCA1. J. Clin. Investig. *125*, 2748–2758. https://doi.org/10.1172/jci80300.
15. Luo, B., Gan, W., Liu, Z., Shen, Z., Wang, J., Shi, R., Liu, Y., Liu, Y., Jiang, M., Zhang, Z., and Wu, Y. (2016). Erythropoeitin Signaling in Macrophages Promotes Dying Cell Clearance and Immune Tolerance. Immunity *44*, 287–302. https://doi.org/10.1016/j.immuni.2016.01.002.
16. Lee, C.S., Penberthy, K.K., Wheeler, K.M., Juncadella, I.J., Vandenabeele, P., Lysiak, J.J., and Ravichandran, K.S. (2016). Boosting Apoptotic Cell Clearance by Colonic Epithelial Cells Attenuates Inflammation In Vivo. Immunity *44*, 807–820. https://doi.org/10.1016/j.immuni.2016.02.005.
17. Werfel, T.A., Elion, D.L., Rahman, B., Hicks, D.J., Sanchez, V., Gonzales-Ericsson, P.I., Nixon, M.J., James, J.L., Balko, J.M., Scherle, P.A., et al. (2019). Treatment-Induced Tumor Cell Apoptosis and Secondary Necrosis Drive Tumor Progression in the Residual Tumor Microenvironment through MerTK and IDO1. Cancer Res. *79*, 171–182. https://doi.org/10.1158/0008-5472.Can-18-1106.
18. Zhou, Y., Fei, M., Zhang, G., Liang, W.C., Lin, W., Wu, Y., Piskol, R., Ridgway, J., McNamara, E., Huang, H., et al. (2020). Blockade of the Phagocytic Receptor MerTK on Tumor-Associated Macrophages Enhances P2X7R-Dependent STING Activation by Tumor-Derived cGAMP. Immunity *52*, 357–373.e9. https://doi.org/10.1016/j.immuni.2020.01.014.
19. Cieza, A., Causey, K., Kamenov, K., Hanson, S.W., Chatterji, S., and Vos, T. (2021). Global estimates of the need for rehabilitation based on the Global Burden of Disease study 2019: a systematic analysis for the Global Burden of Disease Study 2019. Lancet *396*, 2006–2017. https://doi.org/10.1016/s0140-6736(20)32340-0.

20. Chou, R. (2021). Low Back Pain. Ann. Intern. Med. *174*, ITC113–ITC128. https://doi.org/10.7326/aitc202108170.

21. Knezevic, N.N., Candido, K.D., Vlaeyen, J.W.S., Van Zundert, J., and Cohen, S.P. (2021). Low back pain. Lancet (London, England) *398*, 78–92. https://doi.org/10.1016/s0140-6736(21)00733-9.

22. Roughley, P.J. (2004). Biology of intervertebral disc aging and degeneration: involvement of the extracellular matrix. Spine *29*, 2691–2699. https://doi.org/10.1097/01.brs.0000146101.53784.b1.

23. Liang, H., Luo, R., Li, G., Zhang, W., Song, Y., and Yang, C. (2022). The Proteolysis of ECM in Intervertebral Disc Degeneration. Int. J. Mol. Sci. *23*, 1715. https://doi.org/10.3390/ijms23031715.

24. Le Maitre, C.L., Pockert, A., Buttle, D.J., Freemont, A.J., and Hoyland, J.A. (2007). Matrix synthesis and degradation in human intervertebral disc degeneration. Biochem. Soc. Trans. *35*, 652–655. https://doi.org/10.1042/bst0350652.

25. Chen, S., Lei, L., Li, Z., Chen, F., Huang, Y., Jiang, G., Guo, X., Zhao, Z., Liu, H., Wang, H., et al. (2022). Grem1 accelerates nucleus pulposus cell apoptosis and intervertebral disc degeneration by inhibiting TGF-β-mediated Smad2/3 phosphorylation. Exp. Mol. Med. *54*, 518–530. https://doi.org/10.1038/s12276-022-00753-9.

26. Ding, F., Shao, Z.W., Yang, S.H., Wu, Q., Gao, F., and Xiong, L.M. (2012). Role of mitochondrial pathway in compression-induced apoptosis of nucleus pulposus cells. Apoptosis. Apoptosis *17*, 579–590. https://doi.org/10.1007/s10495-012-0708-3.

27. Chen, D., Xia, D., Pan, Z., Xu, D., Zhou, Y., Wu, Y., Cai, N., Tang, Q., Wang, C., Yan, M., et al. (2016). Metformin protects against apoptosis and senescence in nucleus pulposus cells and ameliorates disc degeneration in vivo. Cell Death Dis. *7*, e2441. https://doi.org/10.1038/cddis.2016.334.

28. Mehrotra, P., and Ravichandran, K.S. (2022). Drugging the efferocytosis process: concepts and opportunities. Nat. Rev. Drug Discov. *21*, 601–620. https://doi.org/10.1038/s41573-022-00470-y.

29. Mohamadnejad, M., Namiri, M., Bagheri, M., Hashemi, S.M., Ghanaati, H., Zare Mehrjardi, N., Kazemi Ashtiani, S., Malekzadeh, R., and Baharvand, H. (2007). Phase 1 human trial of autologous bone marrow-hematopoietic stem cell transplantation in patients with decompensated cirrhosis. World J. Gastroenterol. *13*, 3359–3363. https://doi.org/10.3748/wjg.v13.i24.3359.

30. Meier, R.P.H., Mahou, R., Morel, P., Meyer, J., Montanari, E., Muller, Y.D., Christofilopoulos, P., Wandrey, C., Gonelle-Gispert, C., and Bühler, L.H. (2015). Microencapsulated human mesenchymal stem cells decrease liver fibrosis in mice. J. Hepatol. *62*, 634–641. https://doi.org/10.1016/j.jhep.2014.10.030.

31. Liebl, H., and Kloth, L.C. (2012). Skin cell proliferation stimulated by microneedles. J. Am. Coll. Clin. Wound Spec. *4*, 2–6. https://doi.org/10.1016/j.jccw.2012.11.001.

32. Meng, Q., Xie, E., Sun, H., Wang, H., Li, J., Liu, Z., Li, K., Hu, J., Chen, Q., Liu, C., et al. (2024). High-Strength Smart Microneedles with "Offensive and Defensive" Effects for Intervertebral Disc Repair. Adv. Mater. *36*, e2305468. https://doi.org/10.1002/adma.202305468.

33. Wu, D., Li, G., Zhou, X., Zhang, W., Liang, H., Luo, R., Wang, K., Feng, X., Song, Y., and Yang, C. (2022). Repair Strategies and Bioactive Functional Materials for Intervertebral Disc. Adv. Funct. Mater. *32*, 2209471. https://doi.org/10.1002/adfm.202209471.

34. Zhang, Y., Xu, Y., Kong, H., Zhang, J., Chan, H.F., Wang, J., Shao, D., Tao, Y., and Li, M. (2023). Microneedle system for tissue engineering and regenerative medicine. Exploration (Beijing, China) *3*, 20210170. https://doi.org/10.1002/exp.20210170.

35. Zhang, W., Li, G., Wang, B., Zhu, Q., Zeng, L., Wen, Z., Yang, C., and Pan, Y. (2022). Triboelectric Nanogenerators for Cellular Bioelectrical Stimulation. Adv. Funct. Mater. *32*, 2203029. https://doi.org/10.1002/adfm.202203029.

36. Zhou, X., Li, G., Wu, D., Liang, H., Zhang, W., Zeng, L., Zhu, Q., Lai, P., Wen, Z., Yang, C., and Pan, Y. (2023). Recent advances of cellular stimulation with triboelectric nanogenerators. Exploration (Beijing, China) *3*, 20220090. https://doi.org/10.1002/exp.20220090.

37. Shen, J., Xu, S., Zhou, H., Liu, H., Jiang, W., Hao, J., and Hu, Z. (2017). IL-1β induces apoptosis and autophagy via mitochondria pathway in human degenerative nucleus pulposus cells. Sci. Rep. *7*, 41067. https://doi.org/10.1038/srep41067.

38. Risbud, M.V., and Shapiro, I.M. (2014). Role of cytokines in intervertebral disc degeneration: pain and disc content. Nat. Rev. Rheumatol. *10*, 44–56. https://doi.org/10.1038/nrrheum.2013.160.

39. Pfirrmann, C.W., Metzdorf, A., Zanetti, M., Hodler, J., and Boos, N. (2001). Magnetic resonance classification of lumbar intervertebral disc degeneration. Spine *26*, 1873–1878. https://doi.org/10.1097/00007632-200109010-00011.

40. Rutges, J.P.H.J., Duit, R.A., Kummer, J.A., Bekkers, J.E.J., Oner, F.C., Castelein, R.M., Dhert, W.J.A., and Creemers, L.B. (2013). A validated new histological classification for intervertebral disc degeneration. Osteoarthr. Cartil. *21*, 2039–2047. https://doi.org/10.1016/j.joca.2013.10.001.

41. Huang, Z.N., Wang, Z.Y., Cheng, X.F., Huang, Z.Z., Han, Y.L., Cui, Y.Z., Liu, B., and Tian, W. (2023). Melatonin alleviates oxidative stress-induced injury to nucleus pulposus-derived mesenchymal stem cells through activating PI3K/Akt pathway. J. Orthop. Translat. *43*, 66–84. https://doi.org/10.1016/j.jot.2023.10.002.

42. Ying, Z., Naijin, Z., Yuanming, Z., Chunyu, S., Kexin, C., Boquan, W., Shilong, Y., Saien, L., Dong, W., Jiaqi, X., et al. (2023). Deacetylation of Septin4 by SIRT2 (Silent Mating Type Information Regulation 2 Homolog-2) Mitigates Damaging of Hypertensive Nephropathy. Circ. Res. *132*, 601–624. https://doi.org/10.1161/circresaha.122.321591.

43. Vernon-Roberts, B., Moore, R.J., and Fraser, R.D. (2007). The natural history of age-related disc degeneration: the pathology and sequelae of tears. Spine *32*, 2797–2804. https://doi.org/10.1097/BRS.0b013e31815b64d2.

44. Melrose, J., Roberts, S., Smith, S., Menage, J., and Ghosh, P. (2002). Increased Nerve and Blood Vessel Ingrowth Associated With Proteoglycan Depletion in an Ovine Anular Lesion Model of Experimental Disc Degeneration. Spine *27*, 1278–1285.

45. Freemont, A.J., Watkins, A., Le Maitre, C., Baird, P., Jeziorska, M., Knight, M.T.N., Ross, E.R.S., O'Brien, J.P., and Hoyland, J.A. (2002). Nerve growth factor expression and innervation of the painful intervertebral disc. J. Pathol. *197*, 286–292. https://doi.org/10.1002/path.1108.

46. Kokubo, Y., Uchida, K., Kobayashi, S., Yayama, T., Sato, R., Nakajima, H., Takamura, T., Mwaka, E., Orwotho, N., Bangirana, A., and Baba, H. (2008). Herniated and spondylotic intervertebral discs of the human cervical spine: histological and immunohistological findings in 500 en bloc surgical samples. Laboratory investigation. J. Neurosurg. Spine *9*, 285–295. https://doi.org/10.3171/spi/2008/9/9/285.

47. Jiang, W., Glaeser, J.D., Kaneda, G., Sheyn, J., Wechsler, J.T., Stephan, S., Salehi, K., Chan, J.L., Tawackoli, W., Avalos, P., et al. (2023). Intervertebral disc human nucleus pulposus cells associated with back pain trigger neurite outgrowth in vitro and pain behaviors in rats. Sci. Transl. Med. *15*, eadg7020. https://doi.org/10.1126/scitranslmed.adg7020.

48. Tu, J., Li, W., Yang, S., Yang, P., Yan, Q., Wang, S., Lai, K., Bai, X., Wu, C., Ding, W., et al. (2022). Single-Cell Transcriptome Profiling Reveals Multicellular Ecosystem of Nucleus Pulposus during Degeneration Progression. Adv. Sci. *9*, e2103631. https://doi.org/10.1002/advs.202103631.

49. Wei, A., Brisby, H., Chung, S.A., and Diwan, A.D. (2008). Bone morphogenetic protein-7 protects human intervertebral disc cells in vitro from apoptosis. Spine J. *8*, 466–474. https://doi.org/10.1016/j.spinee.2007.04.021.

50. Chanput, W., Mes, J.J., and Wichers, H.J. (2014). THP-1 cell line: An in vitro cell model for immune modulation approach. Int. Immunopharmacol. *23*, 37–45. https://doi.org/10.1016/j.intimp.2014.08.002.

51. Mohd Yasin, Z.N., Mohd Idrus, F.N., Hoe, C.H., and Yvonne-Tee, G.B. (2022). Macrophage polarization in THP-1 cell line and primary

monocytes: A systematic review. Differentiation. *128*, 67–82. https://doi.org/10.1016/j.diff.2022.10.001.

52. Morioka, S., Kajioka, D., Yamaoka, Y., Ellison, R.M., Tufan, T., Werkman, I.L., Tanaka, S., Barron, B., Ito, S.T., Kucenas, S., et al. (2022). Chimeric efferocytic receptors improve apoptotic cell clearance and alleviate inflammation. Cell *185*, 4887–4903.e17. https://doi.org/10.1016/j.cell.2022.11.029.
53. Liao, Z., Liu, H., Ma, L., Lei, J., Tong, B., Li, G., Ke, W., Wang, K., Feng, X., Hua, W., et al. (2021). Engineering Extracellular Vesicles Restore the Impaired Cellular Uptake and Attenuate Intervertebral Disc Degeneration. ACS Nano *15*, 14709–14724. https://doi.org/10.1021/acsnano.1c04514.
54. Maschalidi, S., Mehrotra, P., Keçeli, B.N., De Cleene, H.K.L., Lecomte, K., Van der Cruyssen, R., Janssen, P., Pinney, J., van Loo, G., Elewaut, D., et al. (2022). Targeting SLC7A11 improves efferocytosis by dendritic cells and wound healing in diabetes. Nature *606*, 776–784. https://doi.org/10.1038/s41586-022-04754-6.
55. Shapouri-Moghaddam, A., Mohammadian, S., Vazini, H., Taghadosi, M., Esmaeili, S.A., Mardani, F., Seifi, B., Mohammadi, A., Afshari, J.T., and Sahebkar, A. (2018). Macrophage plasticity, polarization, and function in health and disease. J. Cell. Physiol. *233*, 6425–6440. https://doi.org/10.1002/jcp.26429.
56. Peng, W., Shuo, Z., Weijian, L., Songfeng, C., Xiao, L., Binwu, H., and Zengwu, S. (2022). Selenium Attenuates TBHP-Induced Apoptosis of Nucleus Pulposus Cells by Suppressing Mitochondrial Fission through Activating Nuclear Factor Erythroid 2-Related Factor 2. Oxid. Med. Cell. Longev. *2022*, 7531788. https://doi.org/10.1155/2022/7531788.
57. Run-Ze, Y., Wen-Ning, X., Huo-Liang, Z., Xin-Feng, Z., Bo, L., Lei-Sheng, J., and Sheng-Dan, J. (2020). Involvement of oxidative stress-induced annulus fibrosus cell and nucleus pulposus cell ferroptosis in intervertebral disc degeneration pathogenesis. J. Cell. Physiol. *236*, 2725–2739. https://doi.org/10.1002/jcp.30039.
58. Zhu, D., Li, C., Swanson, A.M., Villalba, R.M., Guo, J., Zhang, Z., Matheny, S., Murakami, T., Stephenson, J.R., Daniel, S., et al. (2015). BAI1 regulates spatial learning and synaptic plasticity in the hippocampus. J. Clin. Investig. *125*, 1497–1508. https://doi.org/10.1172/jci74603.
59. Perry, J.S.A., Morioka, S., Medina, C.B., Iker Etchegaray, J., Barron, B., Raymond, M.H., Lucas, C.D., Onengut-Gumuscu, S., Delpire, E., and Ravichandran, K.S. (2019). Interpreting an apoptotic corpse as anti-inflammatory involves a chloride sensing pathway. Nat. Cell Biol. *21*, 1532–1543. https://doi.org/10.1038/s41556-019-0431-1.
60. A-Gonzalez, N., Quintana, J.A., García-Silva, S., Mazariegos, M., González de la Aleja, A., Nicolás-Ávila, J.A., Walter, W., Adrover, J.M., Crainiciuc, G., Kuchroo, V.K., et al. (2017). Phagocytosis imprints heterogeneity in tissue-resident macrophages. J. Exp. Med. *214*, 1281–1296. https://doi.org/10.1084/jem.20161375.
61. Cummings, R.J., Barbet, G., Bongers, G., Hartmann, B.M., Gettler, K., Muniz, L., Furtado, G.C., Cho, J., Lira, S.A., and Blander, J.M. (2016). Different tissue phagocytes sample apoptotic cells to direct distinct homeostasis programs. Nature *539*, 565–569. https://doi.org/10.1038/nature20138.
62. Rocha, C., Gonçalves, V., da Silva, M., Bañobre-López, M., and Gallo, J. (2022). PLGA-Based Composites for Various Biomedical Applications. Int. J. Mol. Sci. *23*, 2034. https://doi.org/10.3390/ijms23042034.
63. Makadia, H.K., and Siegel, S.J. (2011). Poly Lactic-co-Glycolic Acid (PLGA) as Biodegradable Controlled Drug Delivery Carrier. Polymers *3*, 1377–1397. https://doi.org/10.3390/polym3031377.
64. Sadat Tabatabaei Mirakabad, F., Nejati-Koshki, K., Akbarzadeh, A., Yamchi, M.R., Milani, M., Zarghami, N., Zeighamian, V., Rahimzadeh, A., Alimohammadi, S., Hanifehpour, Y., and Joo, S.W. (2014). PLGA-based nanoparticles as cancer drug delivery systems. Asian Pac. J. Cancer Prev. *15*, 517–535. https://doi.org/10.7314/apjcp.2014.15.2.517.
65. Luo, Z., Sun, W., Fang, J., Lee, K., Li, S., Gu, Z., Dokmeci, M.R., and Khademhosseini, A. (2019). Biodegradable Gelatin Methacryloyl Microneedles for Transdermal Drug Delivery. Adv. Healthc. Mater. *8*, e1801054. https://doi.org/10.1002/adhm.201801054.
66. Yue, K., Trujillo-de Santiago, G., Alvarez, M.M., Tamayol, A., Annabi, N., and Khademhosseini, A. (2015). Synthesis, properties, and biomedical applications of gelatin methacryloyl (GelMA) hydrogels. Biomaterials *73*, 254–271. https://doi.org/10.1016/j.biomaterials.2015.08.045.
67. Lee, K., Xue, Y., Lee, J., Kim, H.J., Liu, Y., Tebon, P., Sarikhani, E., Sun, W., Zhang, S., Haghniaz, R., et al. (2020). A Patch of Detachable Hybrid Microneedle Depot for Localized Delivery of Mesenchymal Stem Cells in Regeneration Therapy. Adv. Funct. Mater. *30*, 2000086. https://doi.org/10.1002/adfm.202000086.
68. Chen, Z., Lv, Z., Zhuang, Y., Saiding, Q., Yang, W., Xiong, W., Zhang, Z., Chen, H., Cui, W., and Zhang, Y. (2023). Mechanical Signal-Tailored Hydrogel Microspheres Recruit and Train Stem Cells for Precise Differentiation. Adv. Mater. *35*, e2300180. https://doi.org/10.1002/adma.202300180.
69. Daniela, L., Christoph, M., Elke, K., Laure C, M., Kan, Y., Peter A, L., Travis, J.,K., Ferry, P.W.,M., Ali, K., and Dietmar, W.,H. (2016). Functionalization, preparation and use of cell-laden gelatin methacryloyl-based hydrogels as modular tissue culture platforms. Nat. Protoc. *11*, 727–746. https://doi.org/10.1038/nprot.2016.037.
70. Weifeng, Z., Xuan, Q., Gaocai, L., Xingyu, Z., Hongyang, L., Di, W., Yu, S., Kangcheng, Z., Kun, W., Xiaobo, F., et al. (2024). Self-powered triboelectric-responsive microneedles with controllable release of optogenetically engineered extracellular vesicles for intervertebral disc degeneration repair. Nat. Commun. *15*, 5736. https://doi.org/10.1038/s41467-024-50045-1.
71. Jiaqi, L., Bingyi, Z., Weijian, W., Yafei, X., Haoyu, W., and Wei, Z. (2024). Improved intervertebral fusion in LLIF rabbit model with a novel titanium cage. Spine J. *24*, 1109–1120. https://doi.org/10.1016/j.spinee.2023.12.011.
72. Rodrigo, A., Gabriel, P., Fernando, M., Rafael, M., Jullyene, P., Igor, B., Weby, M., Marcelo, Y., Sebastião, F., and Luiz, P. (2023). Lateral versus posterior approaches to treat degenerative lumbar pathologies-systematic review and meta-analysis of recent literature. Eur. Spine J. *32*, 1655–1677. https://doi.org/10.1007/s00586-023-07619-2.
73. Isaac, L.,M., Yejia, Z., Peng, S., Ana, C., Marge J, P., and Howard, S.,A. (2013). Retroperitoneal approach to the intervertebral disc for the annular puncture model of intervertebral disc degeneration in the rabbit. Spine J. *13*, 229–234. https://doi.org/10.1016/j.spinee.2012.02.028.
74. Liang, M., Yu, C., Xiaobo, F., Xiaoguang, Z., Jie, L., Hongchuan, W., Yan, X., Bide, T., Dingchao, Z., Di, W., et al. (2023). A Janus-ROS Healing System Promoting Infectious Bone Regeneration via Sono-Epigenetic Modulation. Adv. Mater *36*, e2307846. https://doi.org/10.1002/adma.202307846.
75. Jie, L., Weifeng, Z., Liang, M., Yaqi, H., Huaizhen, L., Xiaoguang, Z., Gaocai, L., Xiaobo, F., Lei, T., and Cao, Y. (2023). Sonodynamic amplification of cGAS-STING activation by cobalt-based nanoagonist against bone and metastatic tumor. Biomaterials *302*, 122295. https://doi.org/10.1016/j.biomaterials.2023.122295.
76. Binch, A.L.A., Fitzgerald, J.C., Growney, E.A., and Barry, F. (2021). Cell-based strategies for IVD repair: clinical progress and translational obstacles. Nat. Rev. Rheumatol. *17*, 158–175. https://doi.org/10.1038/s41584-020-00568-w.
77. Wang, Y., Che, M., Xin, J., Zheng, Z., Li, J., and Zhang, S. (2020). The role of IL-1β and TNF-α in intervertebral disc degeneration. Biomed. Pharmacother. *131*, 110660. https://doi.org/10.1016/j.biopha.2020.110660.
78. Traeger, A.C., Underwood, M., Ivers, R., and Buchbinder, R. (2022). Low back pain in people aged 60 years and over. BMJ (Clinical research ed.) *376*, e066928. https://doi.org/10.1136/bmj-2021-066928.
79. Lyu, F.J., Cui, H., Pan, H., Mc Cheung, K., Cao, X., Iatridis, J.C., and Zheng, Z. (2021). Painful intervertebral disc degeneration and inflammation: from laboratory evidence to clinical interventions. Bone Res. *9*, 7. https://doi.org/10.1038/s41413-020-00125-x.
80. Morioka, S., Perry, J.S.A., Raymond, M.H., Medina, C.B., Zhu, Y., Zhao, L., Serbulea, V., Onengut-Gumuscu, S., Leitinger, N., Kucenas, S., et al. (2018). Efferocytosis induces a novel SLC program to promote glucose

CellPress
OPEN ACCESS

uptake and lactate release. Nature *563*, 714–718. https://doi.org/10.1038/s41586-018-0735-5.

81. Bagaitkar, J., Huang, J., Zeng, M.Y., Pech, N.K., Monlish, D.A., Perez-Zapata, L.J., Miralda, I., Schuettpelz, L.G., and Dinauer, M.C. (2018). NADPH oxidase activation regulates apoptotic neutrophil clearance by murine macrophages. Blood *131*, 2367–2378. https://doi.org/10.1182/blood-2017-09-809004.

82. Das, S., Sarkar, A., Ryan, K.A., Fox, S., Berger, A.H., Juncadella, I.J., Bimczok, D., Smythies, L.E., Harris, P.R., Ravichandran, K.S., et al. (2014). Brain angiogenesis inhibitor 1 is expressed by gastric phagocytes during infection with Helicobacter pylori and mediates the recognition and engulfment of human apoptotic gastric epithelial cells. FASEB J. *28*, 2214–2224. https://doi.org/10.1096/fj.13-243238.

83. Kim, H.J., Yang, K., Kim, K., Lee, Y.J., Lee, S., Ahn, S.Y., Ahn, Y.H., and Kang, J.L. (2022). Reprogramming of cancer-associated fibroblasts by apoptotic cancer cells inhibits lung metastasis via Notch1-WISP-1 signaling. Cell. Mol. Immunol. *19*, 1373–1391. https://doi.org/10.1038/s41423-022-00930-w.

84. Imke, L., Amirah, A.J., Christian, C., Clarissa, L., Stephanie, L., Madeleine, H., Milagros N, W., Dominik, K., Stefanie K, G., Ilka, E., et al. (2024). Apoptotic cell identity induces distinct functional responses to IL-4 in efferocytic macrophages. Science *384*, eabo7027. https://doi.org/10.1126/science.abo7027.

85. McHugh, J. (2023). Cognitive functional therapy improves low back pain. Nat. Rev. Rheumatol. *19*, 397. https://doi.org/10.1038/s41584-023-00988-4.

86. Bagg, M.K., Wand, B.M., Cashin, A.G., Lee, H., Hübscher, M., Stanton, T.R., O'Connell, N.E., O'Hagan, E.T., Rizzo, R.R.N., Wewege, M.A., et al. (2022). Effect of Graded Sensorimotor Retraining on Pain Intensity in Patients With Chronic Low Back Pain: A Randomized Clinical Trial. JAMA *328*, 430–439. https://doi.org/10.1001/jama.2022.9930.

87. Knotkova, H., Hamani, C., Sivanesan, E., Le Beuffe, M.F.E., Moon, J.Y., Cohen, S.P., and Huntoon, M.A. (2021). Neuromodulation for chronic pain. Lancet (London, England) *397*, 2111–2124. https://doi.org/10.1016/s0140-6736(21)00794-7.

88. DiStefano, T.J., Shmukler, J.O., Danias, G., Di Pauli von Treuheim, T., Hom, W.W., Goldberg, D.A., Laudier, D.M., Nasser, P.R., Hecht, A.C., Nicoll, S.B., and Iatridis, J.C. (2020). Development of a two-part biomaterial adhesive strategy for annulus fibrosus repair and ex vivo evaluation of implant herniation risk. Biomaterials *258*, 120309. https://doi.org/10.1016/j.biomaterials.2020.120309.

89. Wang, L., Jin, K., Li, N., Xu, P., Yuan, H., Ramaraju, H., Hollister, S.J., and Fan, Y. (2023). Innovative design of minimal invasive biodegradable poly(glycerol-dodecanoate) nucleus pulposus scaffold with function regeneration. Nat. Commun. *14*, 3865. https://doi.org/10.1038/s41467-023-39604-0.

90. Kamali, A., Ziadlou, R., Lang, G., Pfannkuche, J., Cui, S., Li, Z., Richards, R.G., Alini, M., and Grad, S. (2021). Small molecule-based treatment approaches for intervertebral disc degeneration: Current options and future directions. Theranostics *11*, 27–47. https://doi.org/10.7150/thno.48987.

91. Cheng, X., Zhang, L., Zhang, K., Zhang, G., Hu, Y., Sun, X., Zhao, C., Li, H., Li, Y.M., and Zhao, J. (2018). Circular RNA VMA21 protects against intervertebral disc degeneration through targeting miR-200c and X linked inhibitor-of-apoptosis protein. Ann. Rheum. Dis. *77*, 770–779. https://doi.org/10.1136/annrheumdis-2017-212056.

92. Kerur, N., Fukuda, S., Banerjee, D., Kim, Y., Fu, D., Apicella, I., Varshney, A., Yasuma, R., Fowler, B.J., Baghdasaryan, E., et al. (2018). cGAS drives noncanonical-inflammasome activation in age-related macular degeneration. Nat. Med. *24*, 50–61. https://doi.org/10.1038/nm.4450.

93. Gotaro, T., Toshimasa, Y., Takashi, K., and Kohjiro, U. (2021). Preparation and culture of bone marrow-derived macrophages from mice for functional analysis. STAR Protoc. *2*, 100246. https://doi.org/10.1016/j.xpro.2020.100246.

94. Stoyan, I., Alexandre, G., Marilyn, G., Marion I, S., Johanna, M., Nathalie, V., Laurent, Y.-C., and Rodolphe R, G. (2019). Mesothelial cell CSF1 sustains peritoneal macrophage proliferation. Eur. J. Immunol. *49*, 2012–2018. https://doi.org/10.1002/eji.201948164.

95. Chen, S., Wu, X., Lai, Y., Chen, D., Bai, X., Liu, S., Wu, Y., Chen, M., Lai, Y., Cao, H., et al. (2022). Kindlin-2 inhibits Nlrp3 inflammasome activation in nucleus pulposus to maintain homeostasis of the intervertebral disc. Bone Res. *10*, 5. https://doi.org/10.1038/s41413-021-00179-5.

96. Li, G., Ma, L., He, S., Luo, R., Wang, B., Zhang, W., Song, Y., Liao, Z., Ke, W., Xiang, Q., et al. (2022). WTAP-mediated mA modification of lncRNA NORAD promotes intervertebral disc degeneration. Nat. Commun. *13*, 1469. https://doi.org/10.1038/s41467-022-28990-6.

97. Yu, W., Ye, W., Mingyan, D., and Qingquan, K. (2021). Establishment of a Rabbit Intervertebral Disc Degeneration Model by Percutaneous Posterolateral Puncturing of Lumbar Discs Under Local Anesthesia. World Neurosurg. *154*, e830–e837. https://doi.org/10.1016/j.wneu.2021.08.024.

STAR★METHODS

KEY RESOURCES TABLE

REAGENT or RESOURCE	SOURCE	IDENTIFIER
Antibodies		
Anti-GAPDH Antibody	Boster	Cat#BM3876
GAPDH Monoclonal antibody	Proteintech	Cat#60004-1-Ig; RRID: AB_2107436
Alpha Tubulin Monoclonal antibody	Proteintech	Cat#66031-1-Ig; RRID: AB_11042766
Beta Actin Monoclonal antibody	Proteintech	Cat#66009-1-Ig; RRID: AB_2687938
FLAG® tag Monoclonal antibody	Proteintech	Cat#66008-4-Ig; RRID: AB_2918475
HA Tag Recombinant antibody	Proteintech	Cat#81290-1-RR; RRID: AB_2935602
Bcl2 Polyclonal antibody	Proteintech	Cat#26593-1-AP; RRID: AB_2818996
BAX Polyclonal antibody	Proteintech	Cat#50599-2-Ig; RRID: AB_2061561
Caspase 3/p17/p19 Polyclonal antibody	Proteintech	Cat#19677-1-AP; RRID: AB_10733244
Cleaved-Caspase 3, p17 Antibody	Affinity	Cat# AF7022; RRID: AB_2835326
Anti-MMP3 Antibody	Boster	Cat#BM4074
MMP3 Polyclonal antibody	Proteintech	Cat#17873-1-AP; RRID: AB_2146587
ADAMTS5 Antibody	Affinity	Cat#DF13268; RRID: AB_2846287
Collagen Type II Polyclonal antibody	Proteintech	Cat#28459-1-AP; RRID: AB_2881147
Collagen II Antibody	Affinity	Cat#AF0135; RRID: AB_2833318
Aggrecan Antibody	Affinity	Cat#DF7561; RRID: AB_2841055
CD11b Monoclonal antibody	Proteintech	Cat#66519-1-Ig; RRID: AB_2881882
CD68 Monoclonal antibody	Proteintech	Cat#66231-2-Ig; RRID: AB_2881622
CD86 Polyclonal antibody	Proteintech	Cat#26903-1-AP; RRID: AB_2880677
CD206 Polyclonal antibody	Proteintech	Cat#18704-1-AP; RRID: AB_10597232
BAI1 antibody	Abcam	Cat#Ab135907; RRID: AB_725600
FITC anti-Human CD11b	Biolegend	Cat#301330; RRID: AB_2561703
BV421 anti-Human CD68	BD Pharmingen	Cat#564943; RRID: AB_2739020
PE-Cy7 anti-Human CD86	BD Pharmingen	Cat#561128; RRID: AB_10563077
APC anti-Human CD206	BD Pharmingen	Cat#550889; RRID: AB_398476
Bacterial and virus strains		
pEnCMV-ADGRB1(human)-3×FLAG-SV40-Neo	Miaoling Plasmid	P30778
Biological samples		
Human nucleus pulposus tissues	This paper	Patient demographics shown in Table S1.
Chemicals, peptides, and recombinant proteins		
Annexin V Peptide	Acmec Biochemical	Cat#AC13300
phorbol 12-myristate 13-acetate	MedChemExpress	Cat#HY-18739
Hoechest33342	MedChemExpress	Cat#HY-15559
M-CSF Protein, Human	MedChemExpress	Cat#HY-P7050
M-CSF Protein, Rat	MedChemExpress	Cat#HY-P7386
Rabbit M-CSF Recombinant Protein	Biorbyt	Cat#orb1819800
Human IL-4	Peprotech	Cat#200-04
Human IL-13	Peprotech	Cat#200-13
Human TNF-α	Peprotech	Cat#300-01A
Cypher5E	Cytiva	Cat#PA15401
PKH26	Sigma-Aldrich	Cat#PKH-26GL
PLGA 50/50	Sigma-Aldrich	Cat#900664

(*Continued on next page*)

Continued

REAGENT or RESOURCE	SOURCE	IDENTIFIER
2-Hydroxy40-(2-hydroxyethoxy)-2-methylpropiophenone	Sigma-Aldrich	Cat#410896
GelMA	Sigma-Aldrich	Cat#900629
Zoletil 50	Virbac	N/A
Isoflurane	RWD	N/A
Critical commercial assays		
Human IL-1β ELISA Kit	Elabscience	Cat#E-EL-H0149
Human IL-6 ELISA Kit	Elabscience	Cat#E-EL-H6156
Human IL-10 ELISA Kit	Bioswamp	Cat#HM10203
Human TGF-β ELISA Kit	Bioswamp	Cat#HM10058
Calcein/PI Cell Viability Assay Kit	Beyotime	Cat#C2015L
Annexin V-FITC Apoptosis Detection Kit	BD Biosciences	Cat#556547
Deposited data		
RNA sequencing data	This paper	Mendeley Data: https://data.mendeley.com/datasets/fp33xxkbnk
Experimental models: Cell lines		
Human: THP-1	ATCC	TIB-202
Experimental models: Organisms/strains		
Sprague-Dawley rats	Laboratory Animal Center of Huazhong University of Science and Technology	Two-month-old, male, 200 ± 20 g, SPF-grade
New Zealand White rabbits	Laboratory Animal Center of Huazhong University of Science and Technology	Three-month-old, male, 2.5 ± 0.5 kg, conventional grade
Oligonucleotides		
Primers for real-time PCR analysis, see Table S2	This paper	N/A
siRNA sequence, see Table S2	This paper	N/A
Software and algorithms		
ImageJ	NIH	V2.0.0
FlowJo	BD Biosciences	V10.4.0
GraphPad Prism	GraphPad	V9

EXPERIMENTAL MODEL AND STUDY PARTICIPANT DETAILS

Human NP samples

Human NP samples were collected from 20 volunteers (7 males and 13 females, aged 10 to 68 years) who suffered from idiopathic scoliosis or lumbar disc herniation and spinal stenosis without hereditary diseases, neurologic abnormalities, cancer, infective diseases, autoimmunity and endocrine diseases, and underwent spinal open or minimally invasive surgery (Table S1). Prior to collecting clinical samples and data, we informed each volunteer about the research purpose, experimental procedures, and potential risks associated with the study, and obtain their written consent in accordance with the institutional ethical guidelines. The degenerative grade of human NP samples was assessed using clinical medical records and magnetic resonance imaging (MRI) results based on the Pfirrmann grading system.[39] These samples are mainly used for histological staining, while some of them are used for cell culture and subsequent experiments. Approval for the sample collection was granted by the Ethics Committee at Tongji Medical College, Huazhong University of Science and Technology (No. S341).

Animals

Two-month-old Sprague-Dawley rats (SD rat, male, 200 ± 20 g) were obtained from the Laboratory Animal Center of Huazhong University of Science and Technology and maintained in the specific pathogen–free barrier facility under 12:12 h light:dark cycle at 21°C. All rat experiments were approved by the Laboratory Animal Center of Huazhong University of Science and Technology (No. 3951).

Three-month-old New Zealand White rabbits (2.5 ± 0.5 kg) were obtained from the Laboratory Animal Center of Huazhong University of Science and Technology, acclimatizing to the local environment for at least 1 week. The rabbits were provided with

maintenance ration specifically formulated for rabbits and were reared under ambient room temperature conditions in a conventional environment. All rabbit experiments were approved by the Laboratory Animal Center of Huazhong University of Science and Technology (No. 3952).

METHOD DETAILS

Cell culture and treatments

Human NP samples classified as Grade I were obtained from volunteers who were diagnosed with lumbar vertebral fracture or idiopathic scoliosis, as previous described.[91,92] The specimens were cut into pieces, rinsed with phosphate buffer saline (PBS) twice, treated with 0.4% collagenase II (Invitrogen, USA) for 4 h at 37°C, centrifuged at 800 rpm for 5 min, and rinsed with PBS twice. Cellular precipitates were suspended and grown in 1:1 Dulbecco's Modified Eagle's Medium: F12 (1:1 DMEM: F12; Gibco, USA) containing 10% FBS (Gibco, USA) and 1% penicillin-streptomycin (Sigma-Aldrich, USA) in the incubator at 37°C with 5% CO_2. The culture medium was first replaced after one week. Then, the culture medium was replaced every three days until NP cells covered the bottom of the culture flask and reached over 95% confluence. Cells were passaged at a ratio of 1:3 or 1:4. NP cells in the second passage were regarded as normal state and used to perform experiments.

The human monocyte leukemia cell line (THP-1) was obtained from the ATTC (American Type Tissue Culture Collection, USA). We used RPMI 1640 medium with 10% FBS (Gibco, USA) to culture THP-1 cells at a density of 5×10^5 cells/mL at 37°C and 5% CO_2 and replaced the cell culture medium every two days. THP-1 monocytes were cultured in 6-well plates treated with 100 ng/mL phorbol 12-myristate 13-acetate (PMA) (MedChemExpress, USA) in all experiments for 24 h to become adherent macrophages, TDMs (Figure S2A).

Cytokines were administered at concentrations of 100 ng/mL LPS (Sigma-Aldrich, USA) or 20 ng/mL IL-4 (Peprotech, USA) with 20 ng/mL IL-13 (Peprotech, USA) separately in order to polarize macrophages toward different phenotypes (Figure S4A).

Isolation of primary macrophages

Human bone marrow-derived macrophages (BMDM) were isolated from bone marrow aspirates obtained from volunteers (with informed consent and in accordance with institutional ethical guidelines) undergoing orthopedic surgeries that did not require the bone marrow for diagnostic or therapeutic purposes. BMDMs were isolated using density gradient centrifugation and cultured in RPMI 1640 medium supplemented with 10% fetal bovine serum (FBS; Gibco, USA), 1% penicillin-streptomycin (Sigma-Aldrich, USA), and 20 ng/mL human M-CSF (MedChemExpress, USA) in an incubator at 37°C with 5% CO_2 for a week.[93] The purity of BMDMs was assessed by flow cytometry, calculating the proportion of CD11b+CD68$^+$ cells.

Rat and rabbit peritoneal macrophage were isolated from rat and rabbit peritoneal lavage fluid (PF). PF was collected via peritoneal lavage with 5 mL (for rat) or 10 mL (for rabbit) of ice-cold PBS and immediately placed on ice. PF was centrifuged at 300 g for 5 min at 4°C, and the resulting pellet was resuspended in RPMI 1640 medium containing 10% FBS (Gibco, USA), 1% penicillin-streptomycin (Sigma-Aldrich, USA) and 20 ng/mL rat or rabbit M-CSF (MedChemExpress, USA).[94] The macrophages were allowed to adhere in the incubator at 37°C with 5% CO_2 for a week for subsequent experiments.

Flow cytometry

Trypsin was added to well plates until TDMs became detached and RPMI complete medium was added to neutralize the effects of the enzyme. The cell suspension was centrifuged at 400 × g for 5 min and re-suspended in a volume of 200 μL media. For each sample, 200,000 cells were added into a tube, blocked for 30 min at 4°C and stained with 100 times diluted FITC-conjugated anti-CD11b (Biolegend, USA), BV421-conjugated anti-CD68 (BD Pharmingen, USA), PE-Cy7-conjugated anti-CD86 (BD Pharmingen, USA) and APC-conjugated anti-CD206 (BD Pharmingen, USA) in the dark for 30 min. Cells were subsequently washed for 3 times. Flow cytometry was performed using BD FACSymphony A1 (BD Biosciences, USA) and data was recorded for the corresponding channel.

FlowJo software (BD Biosciences, USA) was utilized for all analyses. All antibodies used are listed in Table S3.

Reverse transcription quantitative polymerase chain reaction (RT-qPCR)

Total RNA from NP cells was extracted with TRIzol reagent (Thermo Fisher Scientific, USA) according to the standard protocols. Complimentary DNA (cDNA) was synthetized using the HiScript III RT SuperMix for qPCR (Vazyme, China). AceQ qPCR SYBR Green Master Mix (Vazyme, China) was utilized for qPCR analysis, with detection of signals performed using the CFX96 Touch sequence detection system (Bio-Rad, USA) and GAPDH was used as an internal control to calculate the relative fold changes in gene expressions. All primers used are listed in Table S2.

Western blotting analysis

Treated cells were lysed with RIPA (Boster, China) supplemented with 1% PMSF (Boster, China) for 30 min on the ice, centrifuged at 12000 rpm for 15 min, and the protein content of supernatant was measured using a BCA protein assay kit (Boster, China). Proteins (30–40 μg) were separated on a 7.5–12.5% SDS-PAGE and moved onto a polyvinylidene difluoride (PVDF) membrane (Millipore, USA). The membrane was blocked with 5% milk and exposed to primary antibodies at 4°C overnight. Following two washes with Tris-buffered saline (TBS) containing 0.1% Tween 20 and incubation with secondary antibodies, proteins were exposed to

chemiluminescence reagents (Affinity, China) and imaged by ChemiDoc MP Imaging System (Bio-Rad, USA). The application and dilution ratios for all antibodies used are listed in Table S3.

Immunofluorescence analysis

NP cells or macrophages were planted in the glass coverslip. After the medium was replaced twice, cells were exposed to indicated treatments. The coverslip was washed twice with PBS, treated with 4% paraformaldehyde for 20 min, made permeable with 0.5% Triton X-100 for 15 min due to the target protein being situated inside the cell, and obstructed with regular goat serum for 30 min. The cells were exposed to primary antibodies at 4°C overnight, rinsed twice with PBS supplemented with 0.1% Tween 20 and incubated with secondary antibodies of anti-mouse/rabbit Alexa Fluor 488 nm or 569 nm. Nuclei were stained with DAPI (Invitrogen, USA). Fluorescence images were captured using a fluorescence microscope (Olympus, Japan).

RNA interference and plasmid transfection

BAI1 protein knockdown in TDMs was performed using small interfering RNA (siRNA) technology (siRNA sequences shown in Table S2). In brief, 50 nM siRNA (General Biol, China) against BAI1 (siBAI1) were transfected to TDMs using Lipofectamine 2000 (Invitrogen, USA) for 72 h. After confirming the highly-efficient silencing, TDMs were used for further analysis.

Human adgrb1 were tagged with FLAG and cloned into the empty vector plasmid pECMV-3×FLAG-C to obtain the overexpression plasmids. For efficiently transient transfection, TDMs were transfected with the previous plasmids using Lipofectamine 2000 (Invitrogen, USA) for 72 h.

Synthesis of GelMA-macrophages

To create the prepolymer solution for the GelMA-macrophages, lyophilized GelMA (Sigma-Aldrich, USA) at a concentration of 10% (w/v) and photoinitiator (2-Hydroxy40-(2-hydroxyethoxy)-2-methylpropiophenone, Sigma-Aldrich, USA) at a concentration of 0.5% (w/v) were dissolved in cell culture medium.[67] Macrophages were added to the prepared GelMA solution to create a prepolymer solution of the GelMA-macrophages.

Fabrication of PLGA shells

Multiple polydimethylsiloxane (PDMS) molds were created with an array of MN cavities measuring height of 1000 μm, pitch of 650 μm, and base diameter of 350 μm for the production of the MN PLGA shell. PLGA50/50 (Sigma-Aldrich, USA) solution with a PLGA-DMSO ratio of 1:3 (w/w) was poured into the mold repeatedly. The PDMS mold surface was treated with O_2 plasma before casting to hydrophilize the surface, enabling the PLGA solution to adhere to the walls of the cavity and fill it. The PLGA was poured onto the mold and then dried in a vacuum to harden the PLGA shell and eliminate any air bubbles. To thicken and strengthen the PLGA shell, the identical procedure was performed again.[67]

Rat coccygeal IVDs needle puncture IDD model

Twenty-gauge needles were used to establish an IDD model in two-month-old SD rats, with needle punctures at the Co7-8, Co8-9, and Co9-10 levels.[95,96] Briefly, rats were anesthetized using 3% pentobarbital and positioned in a prone posture. A twenty-gauge needle punctured the Co7-8, Co8-9, and Co9-10 coccygeal IVDs vertically and parallel to the endplates. The needle was inserted for 5 mm to reach the center of NP region, and rotated by 180° in the axial direction and held for 10 s. Co6-7 and Co10-11 IVDs were left as self-negative control.

Surgical procedure of rabbit lumbar IDD model

Rabbits were anesthetized using marginal ear vein anesthesia with Zoletil 50 (Virbac, France) and gas anesthesia with isoflurane (RWD, China). They were positioned prone, with their limbs secured to the operating table, and the skin over the left side of the lumbar spine was shaved. A longitudinal incision of approximately 7cm was made on the left side of the waist, using the iliac crest as a bony landmark.[97] The L3-L7 vertebral bodies were exposed by carefully separating the paravertebral tissues through the incision.[71] After removing the transverse process, IVD was located, and the surrounding tissues were cleaned. A surgical knife loading 11# blade was then used to cut through the AF tissue and the MNs were subsequently loaded.

In vivo imaging of macrophages

According to the experimental design, randomly selected SD rats were sacrificed, and rat tails were collected. Macrophages *in vivo* imaging at each time point was performed by using an FX PRO imaging system (Bruker, Germany), and the scanning parameters were set at an excitation wavelength of 550 nm and an emission wavelength of 600 nm.

Radiological analysis

X-ray, micro-Computed Tomography (μCT), and MRI analyses were used to evaluate the radiological degenerative degrees of coccygeal IVDs of rats. After 4 weeks of treatment, the rats were anesthetized with 3% pentobarbital and sacrificed for collecting rat tails. X-ray scanning of rat tails was performed using a DRX Ascend system (Carestream, Canada) with the following parameters: exposure time, 0.06 s; distance, 100 cm; current, 160 mA; voltage, 50 kV. The μCT scanning system (SkyScan 1176, Bruker, Germany) was

operated using the following parameters: imaging pixel size, 18 μm; current, 100 μA; voltage, 60 kV. Subsequently, coccygeal IVDs were three-dimensionally restricted using CT-Vox software (Bruker, Germany). T2-weighted imaging was performed using a 7.0 T animal specific MRI system using the following parameters: fast spin echo sequence with time to repetition, 3,000 ms; time to echo, 70 ms; slice thickness, 0.5 mm with a 0 mm gap.

Histological analysis

Human NP specimens were washed with PBS twice and fixed for two days with 4% paraformaldehyde, dehydrated, embedded in paraffin, and cut into 5 μm sections. After radiological analysis, coccygeal IVD specimens of rats were washed with PBS twice and fixed for two days with 4% paraformaldehyde, decalcified with 10% EDTA solution, dehydrated, embedded in paraffin, and cut into 5 μm sections. The sections were stained by hematoxylin/eosin (H&E) and safranin O/fast green (SO&FG) to analyze the degenerative degrees of IVDs. The sections were imaged using the BX53 microscope (Olympus, Japan).

Immunohistochemistry (IHC) staining

Slides of human NP samples or rat coccygeal IVD specimens were deparaffinized with xylene, rehydrated with ethanol in gradient concentrations, antigen-retrieved with citrate buffer, blocked with normal goat serum, and incubated with special primary antibodies overnight. After incubation with secondary antibodies and visualizing using DAB peroxidase substrate kit, histological images were analyzed using a BX53 microscope (Olympus, Japan).

QUANTIFICATION AND STATISTIC ANALYSIS

Apoptosis assays

NP cells were harvested with 0.25% trypsin (Boster, China), followed by staining with an Annexin V-FITC Apoptosis Detection Kit (BD Bioscience, USA) as the provided guidelines. After washing twice with PBS, NP cells were resuspended in 150 μL of binding buffer at a concentration of 6×10^5 cells/mL. After staining with 4 μL of Annexin V-FITC and 10 μL propidium (PI) and incubation for 15 min at room temperature, NP cells were analyzed using BD FACSymphony C6 Plus (BD Bioscience, USA). The apoptotic index was calculated by dividing the total cell count by the number of cells undergoing apoptosis.

After 24 h of growth, NP cells were treated and apoptosis was assessed using the TUNEL assay (Elabscience, China) as the manufacturer's instructions. Briefly, the cells were treated to deparaffinized, hydrated, rinsed with PBS, a and subsequently exposed to the TUNEL reaction solution for 60 min at 37°C. Ultimately, the microscope (Olympus, Japan) was used to analyze the images.

Enzyme-linked immunosorbent assays (ELISA)

Inflammatory cytokine levels were measured in supernatants of co-cultured systems using the Human IL-1β ELISA Kit (Elabscience, China), Human IL-6 ELISA Kit (Elabscience, China), Human IL-10 ELISA Kit (Bioswamp, China) and Human TGF-β ELISA Kit (Bioswamp, China).

Efferocytosis assays

Human apo-NPCs were stained with CypHer5E (Cytiva, USA) or PKH26 (Sigma-Aldrich, USA), and then resuspended in RPMI 1640 medium. Macrophages pre-labeled with Hoechest33342 (MedChemExpress, USA) were incubated with apoptotic targets at a 1:5 ratio of phagocytes to targets for the specified durations. The efferocytosis rates were based on the phagocytosis rates evaluated through a flow cytometry assay or fluorescence imaging.

The analysis of RNA sequencing data and the examination of gene sets using gene set enrichment analysis (GSEA)

As previously described, total RNA was harvested from nine culture TDM samples using TRIzol reagent (Thermo Fisher Scientific, USA). The RNA sequencing library was constructed using 1 μg of RNA and an Illumina TruSeq RNA Sample Prep Kit (Illumina, USA). Libraries were prepared using Illumina HiSeq X Ten accompanying the Ovation RNA-Seq System V2, qualitatively checked by a Bioptic Qse100 Analyzer and sequenced on the Illumina HiSeq X Ten.

GSEA was conducted using GSEA 4.3.2 software with 1000 permutations and default parameters according to standard procedure. The C2 (curated gene sets) collection and C5 (ontology gene set) collection of the Molecular Signatures Database (MSigDB) version 7.5.1 were used to map overlaps between gene sets in MsigDB and expression set.

Mechanical testing of GelMA-macrophages

UV light (14 mW/cm^2) was used to crosslink the standardized gel structure to prepare the samples for mechanical testing. Stiffness of hydrogels was assessed through compressive tests using a universal testing machine (SUNS, China) equipped with a 10 N load cell. The compressive modulus of gels crosslinked for durations ranging from 2 to 7 min was determined.

CellPress
OPEN ACCESS

Macrophage viability test

Macrophage viability was evaluated using a Calcein-AM/PI staining kit (Beyotime, China). A mixture of 0.05% calcein and 0.2% ethidium homodimer-1 in PBS was applied to every well and left to incubate for 30 min. The samples were imaged using a fluorescent microscope (Olympus, Japan) after being washed three times with PBS, to determine the ideal GelMA characteristic.

Statistical analysis

The data in this study were obtained from at least three independent experiments. Unpaired Student's t test was used to analyze the significance between two groups, whereas two-way ANOVA was used to analyze the significance among multiple groups. GraphPad Prism version 9.3.0 was used to analyze data and statistical results. Data are shown as mean ± standard deviation. Asterisks indicate the degree of significance by *p* values: *$p < 0.05$; **$p < 0.01$; ***$p < 0.001$, ****$p < 0.0001$, ns. No significance.

Cell Reports Medicine, Volume *6*

Supplemental information

Microneedle delivery of CAR-M-like engineered macrophages alleviates intervertebral disc degeneration through enhanced efferocytosis capacity

Xingyu Zhou, Dingchao Zhu, Di Wu, Gaocai Li, Huaizhen Liang, Weifeng Zhang, Yali Wu, Hanpeng Xu, Zhengdong Zhang, Bide Tong, Yu Song, Kun Wang, Xiaobo Feng, Jie Lei, Hongchuan Wang, Xiaoguang Zhang, Liang Ma, Yuhang Chen, Junyu Wei, Zixuan Ou, Shuchang Peng, Xinghuo Wu, Lei Tan, Bingjin Wang, and Cao Yang

Supplemental Information

This file includes:

Figure S1 to S12

Table S1 to S3

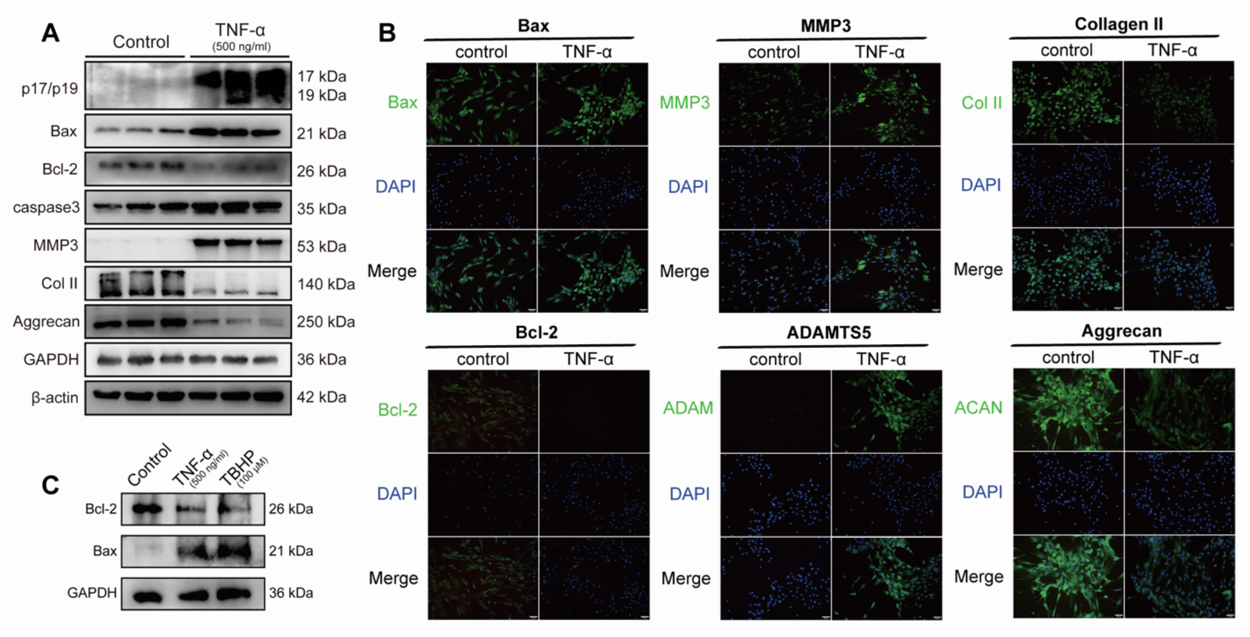

Figure S1. Establishment of apo-NPCs model, related to Figure 1 and the method detail, 'Cell culture and treatments' in STAR Methods.

(A) Representative western blots showing expression of Bax, Bcl-2, caspase3/p17/p19, MMP3, collagen type II and aggrecan in human NPCs treated with 500 ng/mL TNF-α for 24 h. (B) IF staining of Bax, Bcl-2, MMP3, ADAMTS5, Collagen type II, Aggrecan reflecting the success of apo-NPCs model establishment with the treatment of 500ng/mL TNF-α for 24 hours. (C) Representative western blots showing expression of Bax and Bcl-2 in human NPCs treated with 500 ng/mL TNF-α or 100 μM TBHP for 24 h. Scale bars = 100 μm.

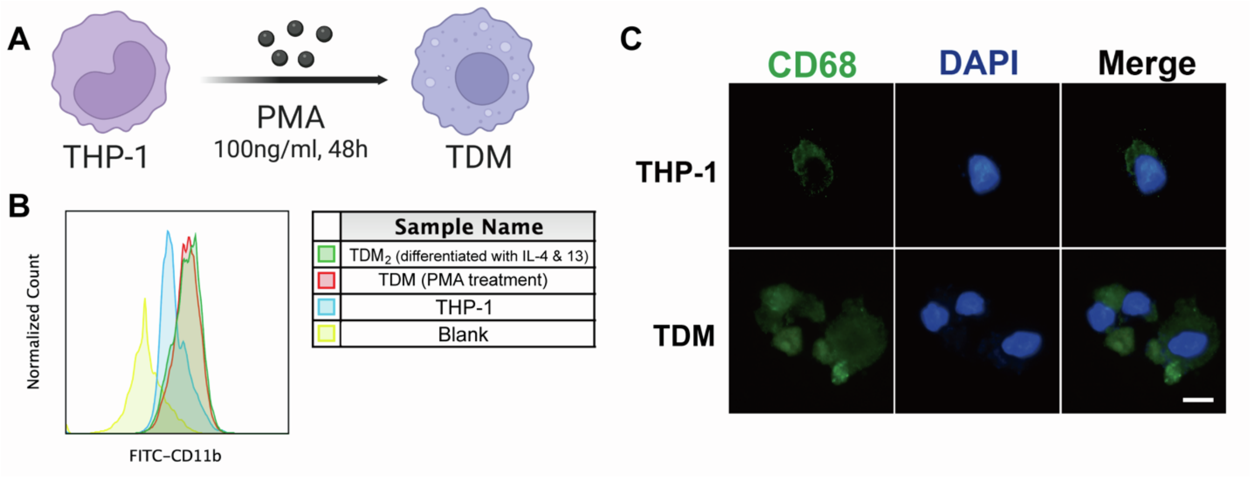

Figure S2. Acquisition of THP-1 derived macrophages, related to Figure 1 and the method detail, 'Cell culture and treatments' in STAR Methods.

(A) Schematic workflow of the acquisition of THP-1 derived macrophages. Created with BioRender.com. Flow cytometry analysis (B) and IF staining images (C) validating the success of the acquisition of TDMs. Scale bars = 10 μm.

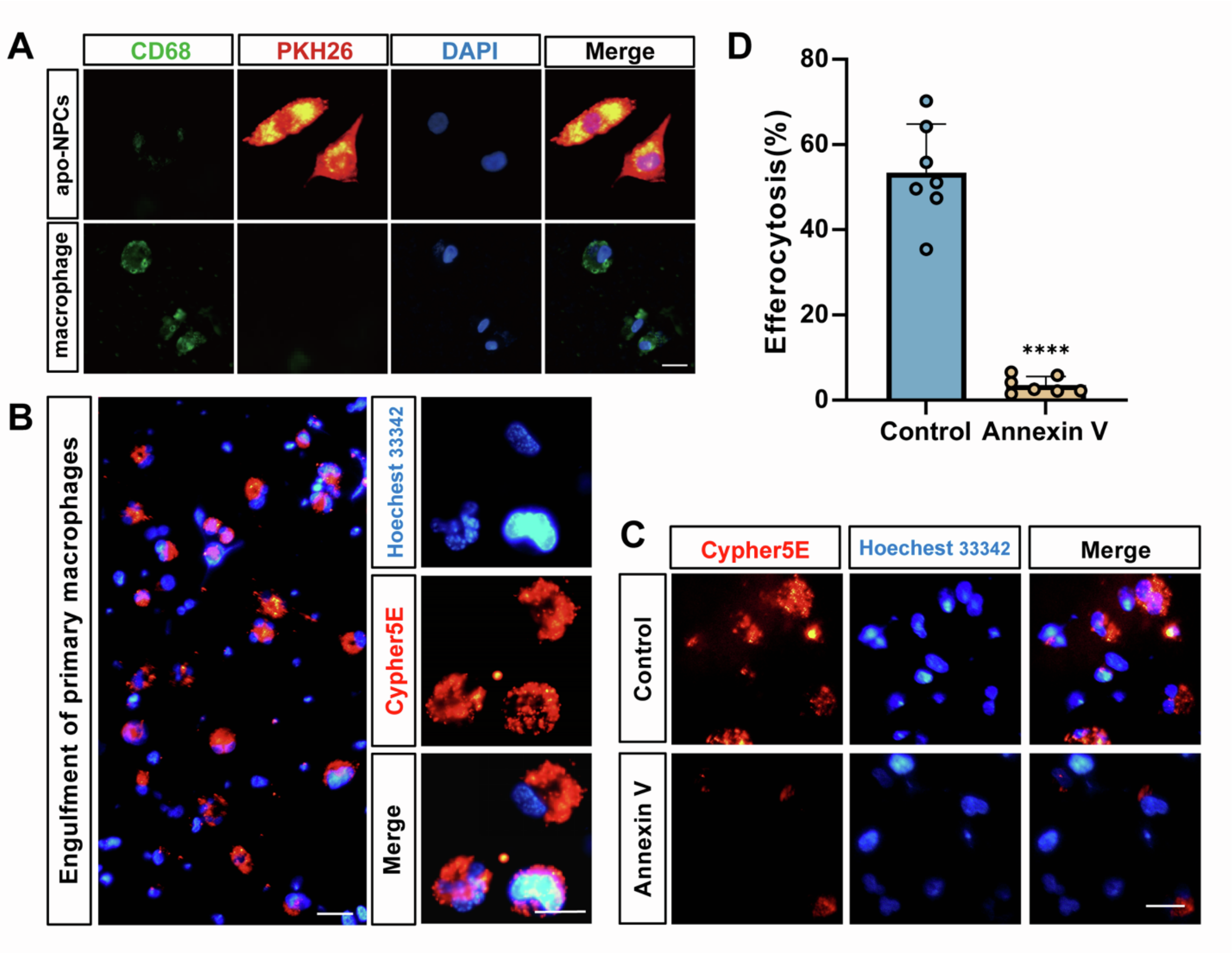

Figure S3. Efferocytosis of primary macrophages, related to Figure 1.

(A) Fluorescence image of PKH26-labeled apo-NPCs and CD68-stained macrophages beforeco-cultured. (B) Hoechest33342-labeled BMDMs co-cultured with Cypher5E-labeled apo-NPCs to show efferocytosis within the NP tissue. (C) Hoechest 33342-labeled BMDMs co-cultured with Cypher5E-labeled apo-NPCs pretreated with or without Annexin V. (D) Efferocytosis rates calculated from analyses of results shown in (C). Scale bars = 20 µm. At least 3 independent experiments were performed. Data represent means ± SD; ****$p < 0.0001$, by Student's *t* test.

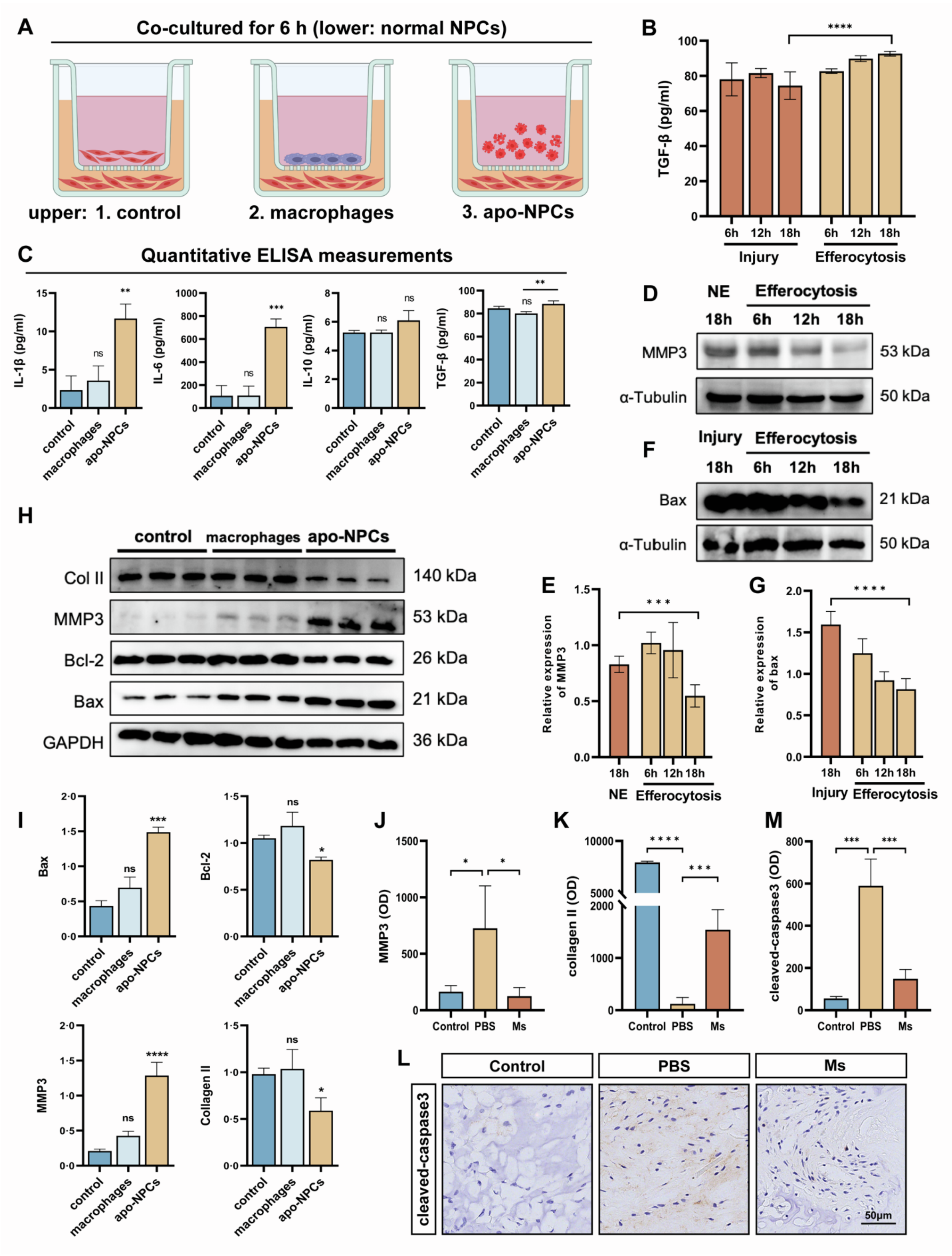
A
Co-cultured for 6 h (lower: normal NPCs)
upper: 1. control
2. macrophages
3. apo-NPCs
B
TGF-β (pg/ml)
Injury
Efferocytosis
C
Quantitative ELISA measurements
IL-1β (pg/ml)
IL-6 (pg/ml)
IL-10 (pg/ml)
TGF-β (pg/ml)
control
macrophages
apo-NPCs
D
NE
Efferocytosis
MMP3
α-Tubulin
53 kDa
50 kDa
F
Injury
Bax
21 kDa
H
Col II
Bcl-2
GAPDH
140 kDa
26 kDa
36 kDa
E
Relative expression of MMP3
G
Relative expression of bax
I
Bax
Bcl-2
MMP3
Collagen II
J
MMP3 (OD)
K
collagen II (OD)
M
cleaved-caspase3 (OD)
Control
PBS
Ms
L
cleaved-caspase3
50μm

Figure S4. Macrophages regulate extracellular matrix synthesis and catabolism through efferocytosis, related to Figure 2.

(A) Schematic graph of the supplementary experimental workflow with the co-culture model without cell-to-cell contact. Created with BioRender.com. (B) Quantitative ELISA measurements of TGF-β in supernatants from injury and efferocytosis groups after 6, 12, 18 hours of co-culture. (C) Quantitative ELISA measurements of IL-1β, IL-6, IL-10 and TGF-β in co-culture supernatants from the supplementary experiment. (D to G) Representative western blots showing expression of MMP3 (D and E) and Bax (F and G) in human NPCs after cultured in the co-cultured model for 6, 12, 18 hours. (H and I) Representative western blots (H) and their statistical analysis results (I) showing expression of Bax, Bcl-2, MMP3, and collagen type II in human NPCs after cultured in the co-cultured model from the supplementary experiment. (J and K) Mean optical density of IHC staining of MMP3 (J) and collagen type II (K) calculated from analyses of results shown in Figure 2N and 2O. (L and M) Representative images (L) and the calculated mean optical density (M) of cleaved-caspase3 IHC staining in rat coccygeal IVDs. Scale bars are indicated separately in each group of images. At least 3 independent experiments were performed. Data represent means ± SD, $*p < 0.05$, $**p < 0.01$, $***p < 0.001$, $****p < 0.0001$, ns. no significance, by two-way ANOVA (C, I to K and M) and Student's *t* test (B, E and G).

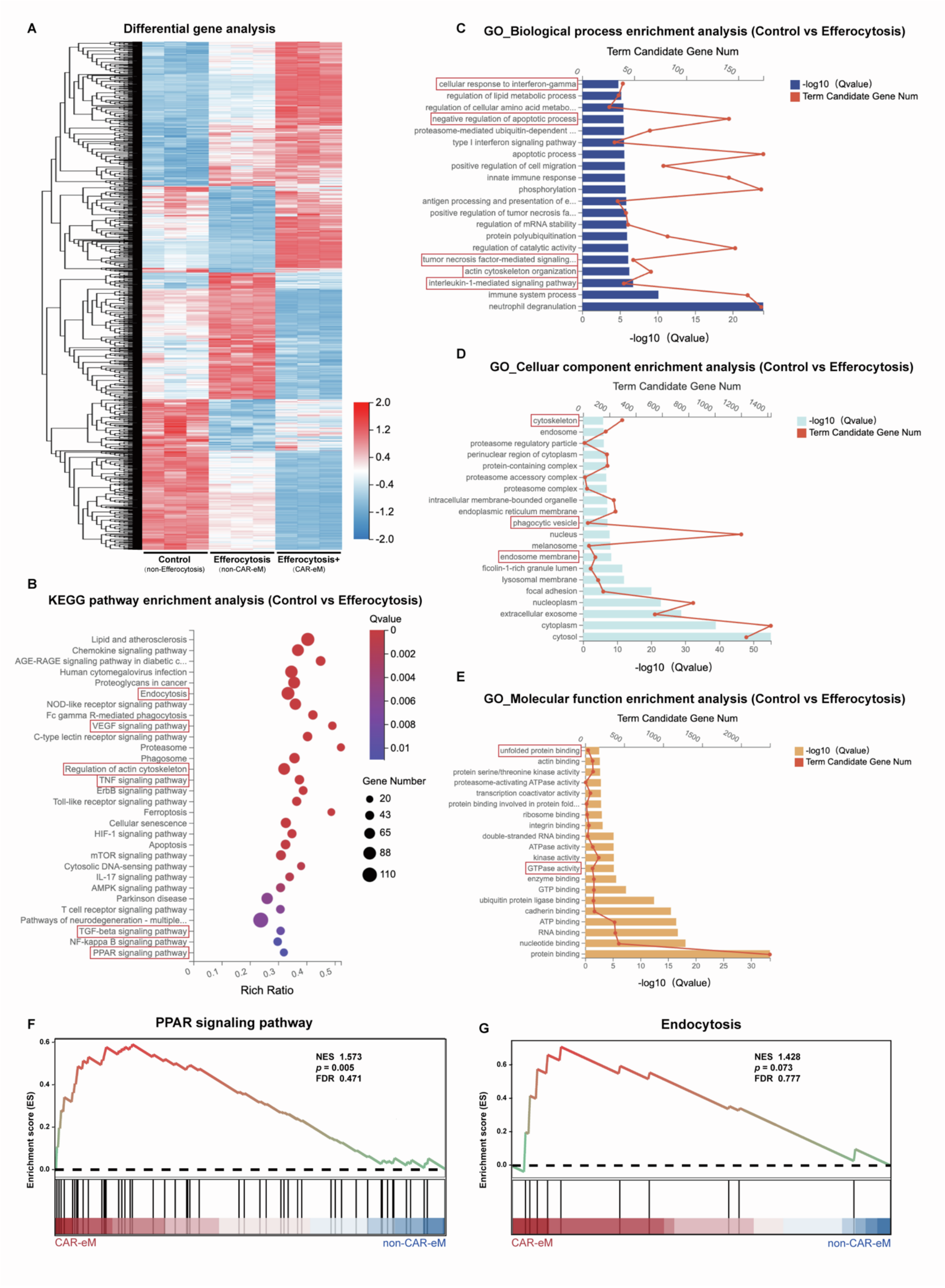
A
Differential gene analysis
2.0
1.2
0.4
-0.4
-1.2
-2.0
Control
(non-Efferocytosis)
Efferocytosis
(non-CAR-eM)
Efferocytosis+
(CAR-eM)
B
KEGG pathway enrichment analysis (Control vs Efferocytosis)
Lipid and atherosclerosis
Chemokine signaling pathway
AGE-RAGE signaling pathway in diabetic c...
Human cytomegalovirus infection
Proteoglycans in cancer
Endocytosis
NOD-like receptor signaling pathway
Fc gamma R-mediated phagocytosis
VEGF signaling pathway
C-type lectin receptor signaling pathway
Proteasome
Phagosome
Regulation of actin cytoskeleton
TNF signaling pathway
ErbB signaling pathway
Toll-like receptor signaling pathway
Ferroptosis
Cellular senescence
HIF-1 signaling pathway
Apoptosis
mTOR signaling pathway
Cytosolic DNA-sensing pathway
IL-17 signaling pathway
AMPK signaling pathway
Parkinson disease
T cell receptor signaling pathway
Pathways of neurodegeneration - multiple...
TGF-beta signaling pathway
NF-kappa B signaling pathway
PPAR signaling pathway
Rich Ratio
Qvalue
0
0.002
0.004
0.006
0.008
0.01
Gene Number
20
43
65
88
110
C
GO_Biological process enrichment analysis (Control vs Efferocytosis)
Term Candidate Gene Num
-log10（Qvalue）
Term Candidate Gene Num
cellular response to interferon-gamma
regulation of lipid metabolic process
regulation of cellular amino acid metabo...
negative regulation of apoptotic process
proteasome-mediated ubiquitin-dependent ...
type I interferon signaling pathway
apoptotic process
positive regulation of cell migration
innate immune response
phosphorylation
antigen processing and presentation of e...
positive regulation of tumor necrosis fa...
regulation of mRNA stability
protein polyubiquitination
regulation of catalytic activity
tumor necrosis factor-mediated signaling...
actin cytoskeleton organization
interleukin-1-mediated signaling pathway
immune system process
neutrophil degranulation
-log10（Qvalue）
D
GO_Celluar component enrichment analysis (Control vs Efferocytosis)
Term Candidate Gene Num
-log10（Qvalue）
Term Candidate Gene Num
cytoskeleton
endosome
proteasome regulatory particle
perinuclear region of cytoplasm
protein-containing complex
proteasome accessory complex
proteasome complex
intracellular membrane-bounded organelle
endoplasmic reticulum membrane
phagocytic vesicle
nucleus
melanosome
endosome membrane
ficolin-1-rich granule lumen
lysosomal membrane
focal adhesion
nucleoplasm
extracellular exosome
cytoplasm
cytosol
-log10（Qvalue）
E
GO_Molecular function enrichment analysis (Control vs Efferocytosis)
Term Candidate Gene Num
-log10（Qvalue）
Term Candidate Gene Num
unfolded protein binding
actin binding
protein serine/threonine kinase activity
proteasome-activating ATPase activity
transcription coactivator activity
protein binding involved in protein fold...
ribosome binding
integrin binding
double-stranded RNA binding
ATPase activity
kinase activity
GTPase activity
enzyme binding
GTP binding
ubiquitin protein ligase binding
cadherin binding
ATP binding
RNA binding
nucleotide binding
protein binding
-log10（Qvalue）
F
PPAR signaling pathway
Enrichment score (ES)
NES 1.573
p = 0.005
FDR 0.471
CAR-eM
non-CAR-eM
G
Endocytosis
Enrichment score (ES)
NES 1.428
p = 0.073
FDR 0.777
CAR-eM
non-CAR-eM

Figure S5. Supplementary molecular mechanisms of efferocytosis therapy, related to Figure 3.

(A) Heatmap of differential gene expression between the control (non-efferocytosis), efferocytosis (non-CAR-eM) and efferocytosis+ (CAR-eM) groups. Top 20 upregulated of KEGG pathway (B), GO_Biological process (C), GO_Cellular component (D) and GO_Molecular function(E) enrichment analysis between the control and efferocytosis groups. (F and G) GSEA showing enrichment of “PPAR signaling pathway” and “Endocytosis” in TDMs between the control and efferocytosis groups.

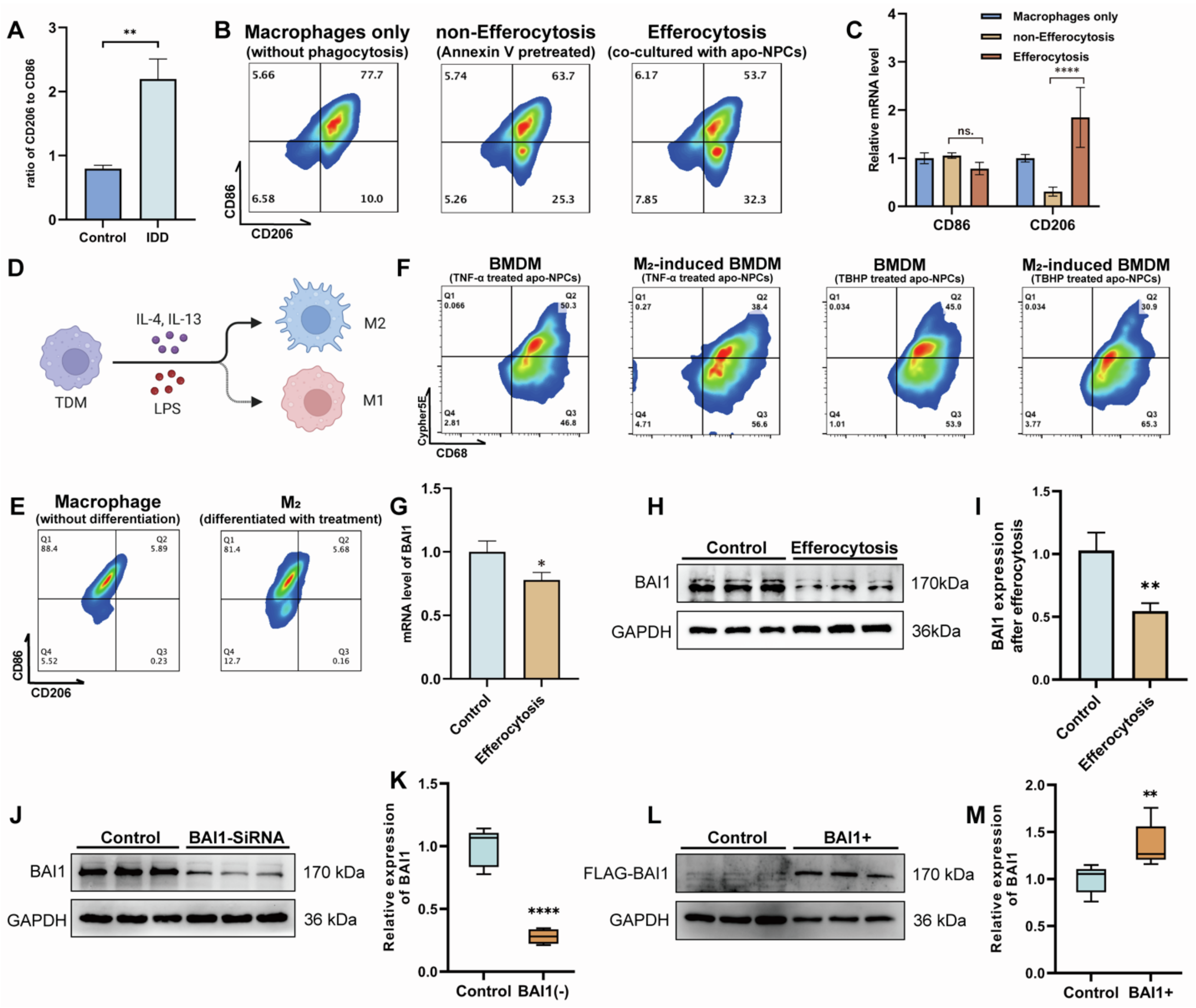

Figure S6. Phenotype transformation, decreased expression of phagocytic receptors, and reduction in phagocytic ability of macrophages after efferocytosis, related to Figure 3 and 4.

(A) Ratio of the amount of CD68+CD206+ cells to CD68+CD86+ cells calculated from analyses of results shown in Figure 4A. (B) Flow cytometry analysis of CD86 and CD206 in BMDMs of each group. (C) RT-qPCR statistical analysis showing the transcription level of CD86, CD206 in TDMs in each group. (D) Schematic workflow of acquisition of macrophage polarization. Created with BioRender.com. (E) Flow cytometry analysis validating the success of the acquisition of M_2-induced BMDMs. (F) Flow cytometry analysis of BMDMs treated with Cypher5E-labeled apo-NPCs treated with TNF-α and TBHP respectively to show the difference in efferocytosis capacity with or without M2 induction. (G) RT-qPCR statistical analysis showing the transcription level of BAI1 in control and efferocytosis groups. (H and I) Representative western blots showing the expression level of BAI1 in BMDMs in control and efferocytosis groups. (J and K) Representative western blot of BAI1 (J) and its statistical analysis (K)

showing expression changes after taking up the BAI1-siRNA. (L and M) Representative western blot of BAI1 (L) and its statistical analysis (M) showing expression changes after the upregulation treatment. At least 3 independent experiments were performed. Data represent means ± SD, *$p < 0.05$, **$p < 0.01$, ****$p < 0.0001$, ns. no significance, by Student's *t* test (A, G, I, K and M) and one-way ANOVA (C).

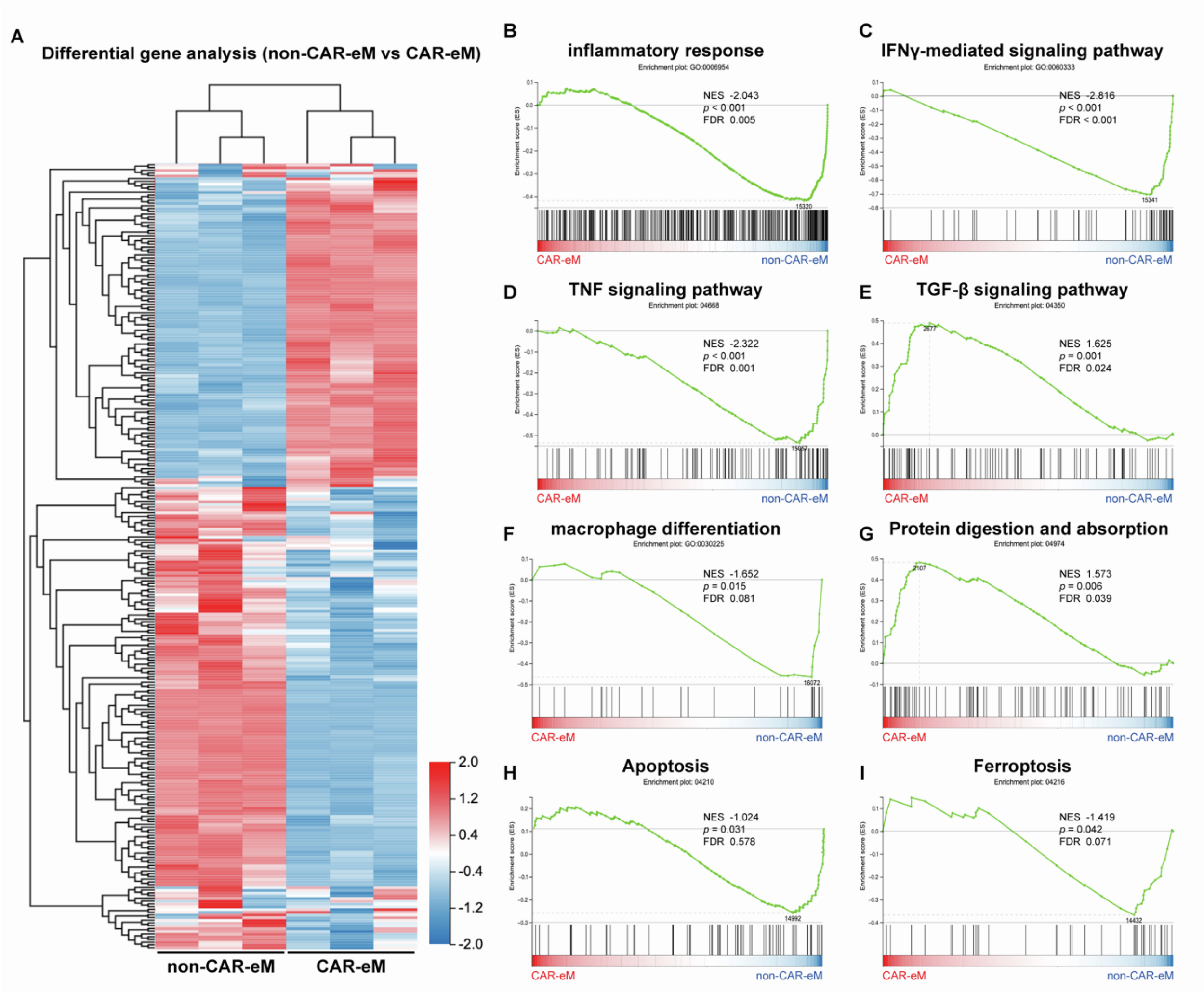

Figure S7. Supplementary molecular mechanisms of CAR-eM therapy, related to Figure 4.

(A) Heatmap of differential gene expression between the non-CAR-eM and CAR-eM groups. (B to I) GSEA showing enrichment of “inflammatory response”, “IFNγ-mediated signaling pathway”, “TNF signaling pathway”, “TGF-β signaling pathway”, “macrophage differentiation”, “Protein digestion and absorption”, “Apoptosis” and “Ferroptosis” in TDMs between the non-CAR-eM and CAR-eM groups.

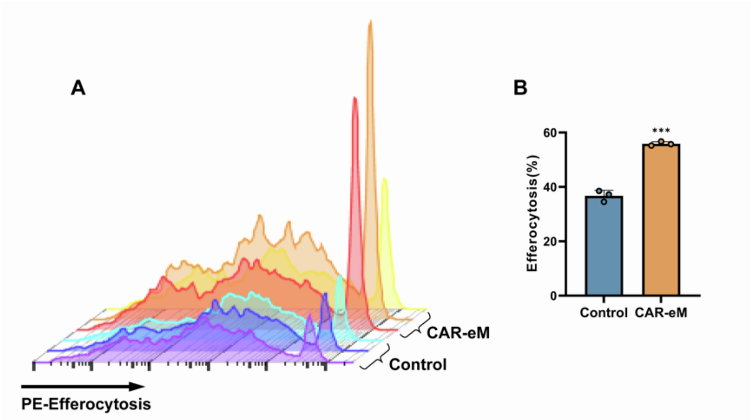

Figure S8. Efferocytosis capacity verification of CAR-eM, related to Figure 5.

(A and B) Flow cytometry analysis (A) of CAR-eMs treated with Cypher5E-labeled apo-NPCs and its calculated efferocytosis rate (B) to show the change in efferocytosis capacity after engineering. Data represent means ± SD, ***$p < 0.001$, by Student's *t* test. Related to Figure 6.

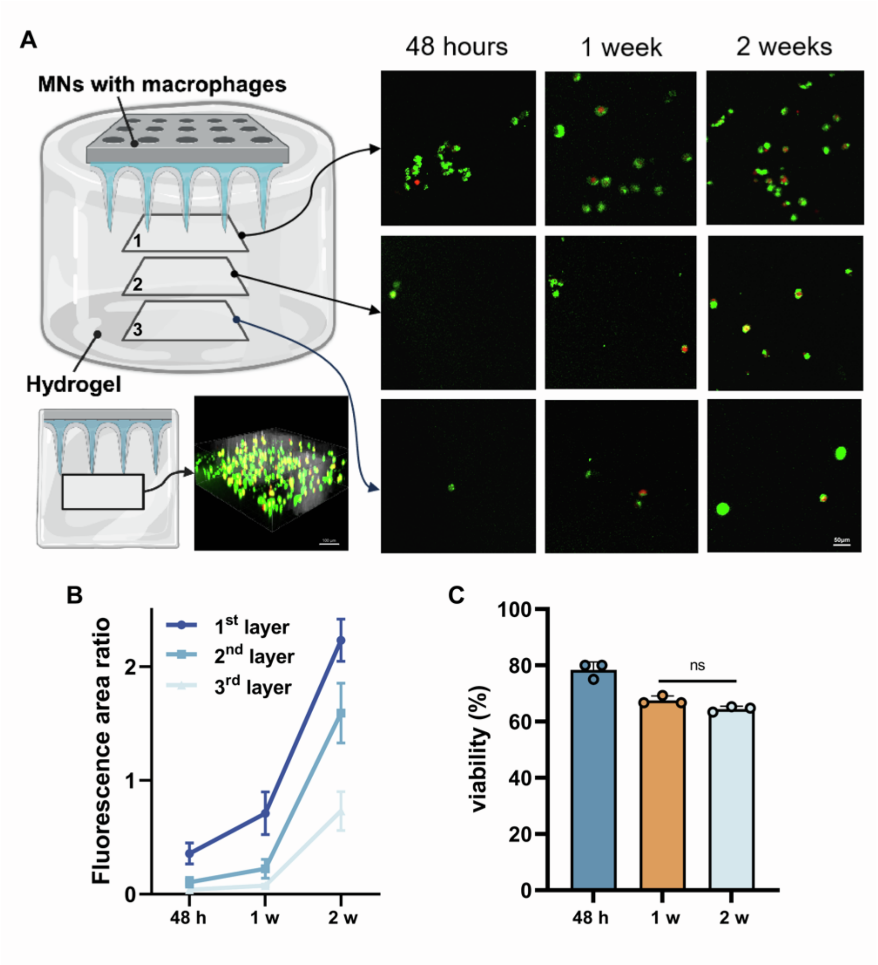

Figure S9. Release and viability of CAR-eMs from CAR-eM-MNs, related to Figure 5.

(A) Schematic graph and representative confocal microscopic images of three layers in 3D hydrogel loaded with CAR-eM-MNs, reflecting the release of CAR-eMs over different application time. (B and C) Fluorescence area ratio (B) and viability of CAR-eMs in hydrogel (C) calculated from analyses of results shown in (A). Scale bars are indicated separately in each group of images. At least 3 independent experiments were performed. Data represent means ± SD, ns. no significance, by Student's *t* test.

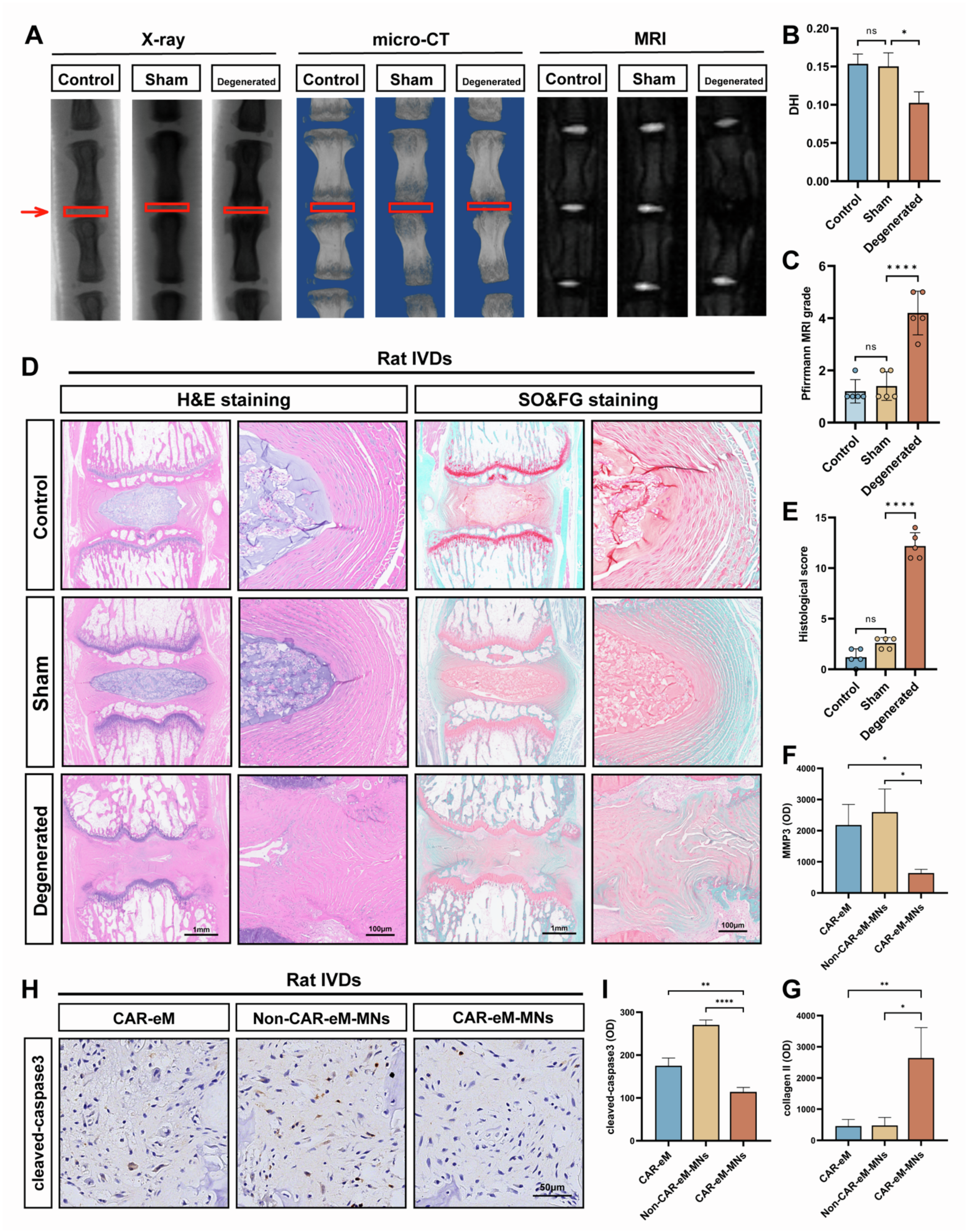

Figure S10. CAR-eM MNs applied in the in vivo treatment of IDD, related to Figure 6.

(A) Representative X-ray images, micro-CT images, and MRI of rat coccygeal IVDs from the control, sham and degenerated groups. (B and C) Disc height index (B) and Pfirrmann degenerative grades (C) of rat coccygeal IVDs in each group. (D and E) Representative H&E staining, SO&FG staining images (D) and histological scores (E) of rat coccygeal IVDs in each group. (F and G) Mean optical density of IHC staining of MMP3 (F) and collagen type II (G) calculated from analyses of results shown in Figure 7J and 7K. (H and I) Representative images (H) and the calculated mean optical density (I) of cleaved-caspase3 IHC staining in rat coccygeal IVDs in each group. Scale bars are indicated separately in each group of images. For animal experiments in this section, n = 5 biological replicates. Data represent means ± SD, *$p < 0.05$, **$p < 0.01$, ****$p < 0.0001$, ns. no significance, by two-way ANOVA.

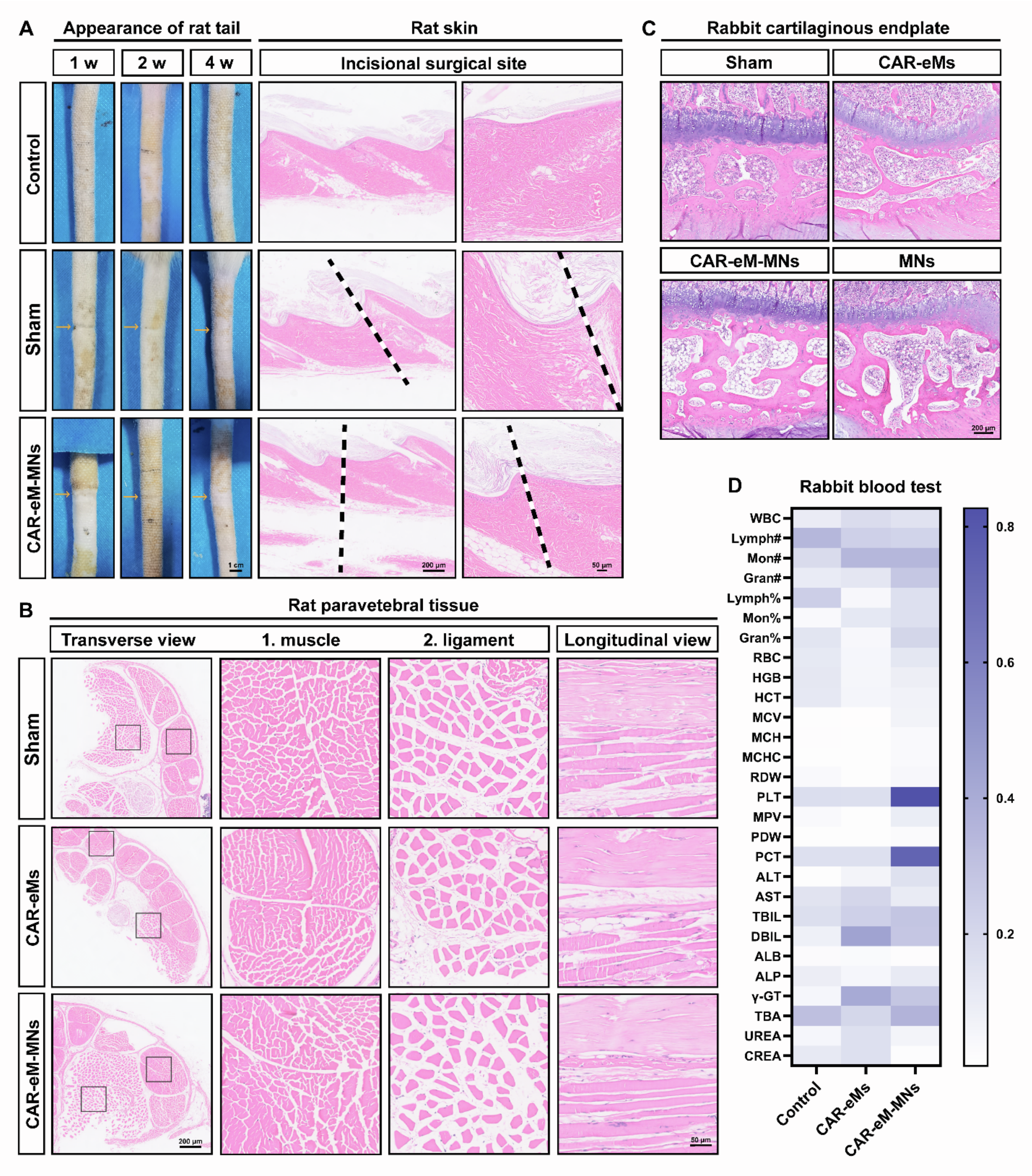

Figure S11. CAR-eM-MNs treatment didn't contribute to unexpected inflammatory response and liver and kidney function injury in vivo, related to Figure 6 and 7.

(A) Representative images of rat tails and the H&E staining of rat skin and subcutaneous tissues from different groups. The incisions are marked. (B) Representative images of the H&E staining of rat paravertebral tissues

including paravertebral muscles and paravertebral ligaments from different groups. (C) Representative images of the H&E staining of rabbit cartilaginous endplate from different groups. (D) Blood routine and biochemical tests on rabbit (n = 4 biological replicates). Scale bars are indicated separately in each group of images.

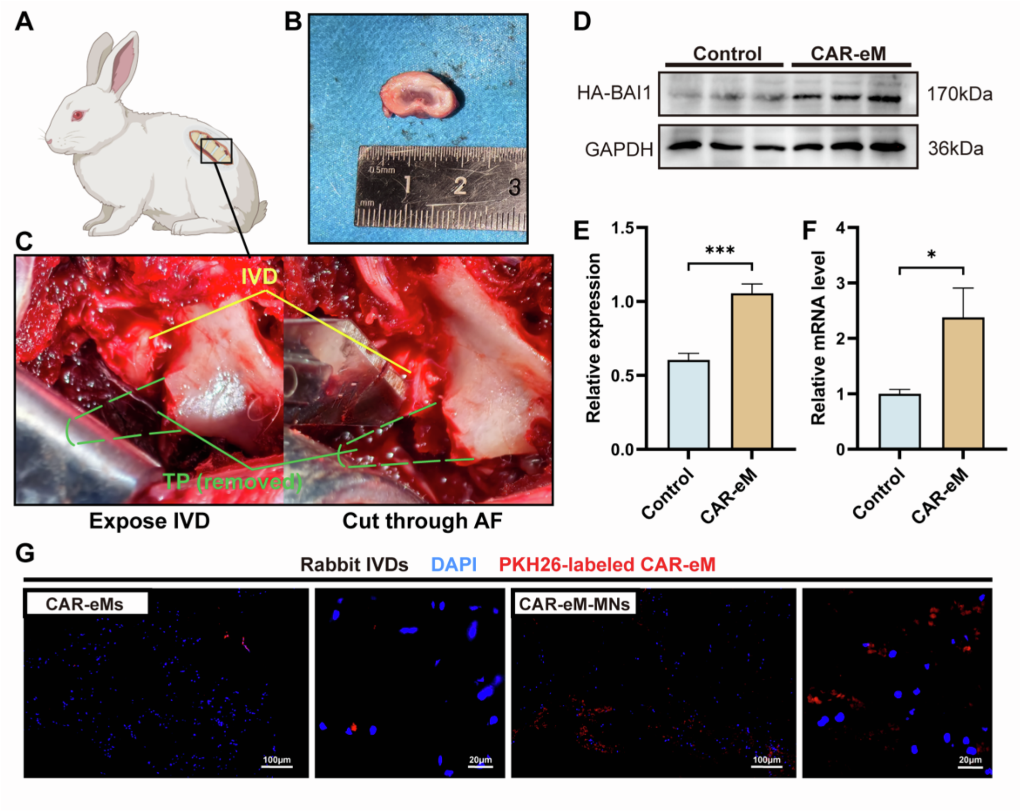

Figure S12. Therapeutic effect of CAR-eM-MNs in lumbar spine surgery, related to Figure 7.

(A) Schematic graph of retroperitoneal approach surgery for rabbits. Created with BioRender.com. (B) Images of rabbit lumbar IVD. (C) Surgical process images of exposing the third to sixth rabbit lumbar vertebrae (L3-L6) and the IVDs from corresponding segments. The positions of IVD and removed transverse process are marked. (D to F) Representative western blot (D and E) and RT-qPCR statistical analysis (F) showing BAI1 expression and its mRNA level in rabbit macrophages to verify the success of the engineered process. (G) Fluorescence images of PKH-26 labeled CAR-eMs delivered by MNs in lumbar IVDs show the long-term localization of macrophages. Scale bars are indicated separately in each image.

Table S1. Patient demographics, related to the experimental model and study participant details 'Human NP samples' in STAR Methods.

Case Number	Gender	Age (years)	Pfirrmann grade	Diagnosis	Health status	Type of consent
Case 1	Female	13	I	Idiopathic scolios	without other related diseases	Preoperative informed consent
Case 2	Male	36	II	Idiopathic scolios	without other related diseases	Preoperative informed consent
Case 3	Female	10	I	Idiopathic scolios	without other related diseases	Preoperative informed consent
Case 4	Female	15	I	Idiopathic scolios	without other related diseases	Preoperative informed consent
Case 5	Female	35	II	Idiopathic scolios	without other related diseases	Preoperative informed consent
Case 6	Male	17	I	Idiopathic scolios	without other related diseases	Preoperative informed consent
Case 7	Male	25	I	Idiopathic scolios	without other related diseases	Preoperative informed consent
Case 8	Male	13	I	Idiopathic scolios	without other related diseases	Preoperative informed consent
Case 9	Female	14	I	Idiopathic scolios	without other related diseases	Preoperative informed consent
Case 10	Female	60	IV	Lumbar disc herniation	without other related diseases	Preoperative informed consent
Case 11	Female	47	III	Lumbar disc herniation	without other related diseases	Preoperative informed consent
Case 12	Female	47	IV	Lumbar disc herniation	without other related diseases	Preoperative informed consent
Case 13	Female	25	III	Lumbar disc herniation	without other related diseases	Preoperative informed consent
Case 14	Male	34	II	Lumbar disc herniation	without other related diseases	Preoperative informed consent
Case 15	Male	53	IV	Lumbar spinal stenosis	without other related diseases	Preoperative informed consent
Case 16	Female	59	IV	Lumbar spinal stenosis	without other related diseases	Preoperative informed consent
Case 17	Female	68	IV	Lumbar spinal stenosis	without other related diseases	Preoperative informed consent
Case 18	Female	44	III	Lumbar spinal stenosis	without other related diseases	Preoperative informed consent
Case 19	Female	57	IV	Lumbar spinal stenosis	without other related diseases	Preoperative informed consent
Case 20	Male	36	III	Lumbar spinal stenosis	without other related diseases	Preoperative informed consent

Table S2. Oligonucleotide sequences used in this study, related to the method details 'RT-qPCR' and 'RNA interference and plasmid transfection' in STAR Methods.

Oligonucleotide	Sense (5'-3')	Antisense (3'-5')
**Primers for qPCR**		
Homo *IL-1β*	GGTTGAGTTTAAGCCAATCCA	TGCTGACCTAGGCTTGATGA
Homo *IL-6*	GCCCAGCTATGAACTCCTTCT	GAAGGCAGCAGGCAACAC
Homo *TGF-β*	GCAACAATTCCTGGCGATACC	CCGGTAGTGAACCCGTTGATG
Homo *IL-10*	TGCCTTCAGCAGAGTGAAGA	GCTTGGCAACCCAGGTAA
Homo *iNOS*	CGTGGAGACGGGAAAGAAGT	GACCCCAGGCAAGATTTGGA
Homo *arg-1*	TCTCAAAGGGACAGCCACGA	CGCTTGCTTTTCCCACAGAC
Homo *TNF-α*	CAGCCTCTTCTCCTTCCTGAT	GCCAGAGGGCTGATTAGAGA
Homo *CD86*	TCCCCCAGACCACATTCCTT	TCCATTGTGTTGGTTCCACATT
Homo *CD206*	TGCTACTGAACCCCCACAAC	ACCAGAGAGGAACCCATTCG
Homo *MMP3*	CAAAACATATTTCTTTGTAGAGGACAA	TTCAGCTATTTGCTTGGGAA
Homo *ADAMTS5*	TCCTGTTTACTCGGGAGGATT	GTCATGGGAGAGGCCAAGTA
Homo *Collagen II*	CCAGGACCAAAGGGACAGAA	ATCCCCTCTGGGTCCTTGTT
Homo *Aggrecan*	GCGAGCACTGTAACATAGACATT	GCCCTCCTCACATACCTCCT
Homo *BAI1*	TGCAGAACTGGACTTTGAGAAGA	CTCCTTGTCCTTCTCCGCTG
Rattus *BAI1*	TGTGCAGAACTTTGTCCAGATCA	CAAAGTCTTCCACCAGTCGAAAG
Homo *GAPDH*	CAAGAAGGTGAAGCAGG	TCAAAGGTGGAGGAGTGGGT
**siRNA sequence**		
Homo-siControl	UUCUCCGAACGUGUCACGU	ACGUGACACGUUCGGAGAA
Homo-siBAI1	CUGCACUGGUUGUGGCCAU	AUGGCCACAACCAGUGCAG

Table S3. Antibody information, related to the method details 'Western blotting analysis' and 'Flow cytometry' in STAR Methods.

Antibody	Company	Catalog#	Application/ Dilution
anti-GAPDH	Boster	BM3876	WB (1: 5000)
anti-β-actin	Boster	BM5422	WB (1: 10000)
anti-Flag tag epitope	Proteintech	66008-4-Ig	WB (1: 20000)
anti-HA tag	Proteintech	81290-1-RR	WB (1: 30000)
anti-Bcl-2	Proteintech	26593-1-AP	WB (1: 1000), IF (1: 200)
anti-Bax	Proteintech	50599-2-Ig	WB (1: 5000), IF (1: 200)
anti-Caspase3	Proteintech	19677-1-AP	WB (1: 500)
anti-MMP3	Boster	BM4074	WB (1: 2000), IHC (1: 100), IF (1: 200)
anti-ADAMTS5	Affinity	DF13268	WB (1: 500), IF (1: 200)
anti-Collagen II	Proteintech	28459-1-AP	IHC (1: 900)
anti-Collagen II	Affinity	AF0135	WB (1: 500), IF (1: 200)
anti-Aggrecan	Affinity	DF7561	WB (1: 1000), IF (1: 200)
anti-CD11b	Proteintech	66519-1-Ig	WB (1: 1000), IF (1: 500)
anti-CD68	Proteintech	66231-2-Ig	IF (1: 12000)
anti-CD86	Proteintech	26903-1-AP	WB (1: 500), IF (1: 200)
anti-CD206	Proteintech	18704-1-AP	WB (1: 500), IF (1: 200)
anti-BAI1	Abcam	Ab135907	WB (1: 100)
FITC anti-Human CD11b	Biolegend	301330	FC
BV421 anti-Human CD68	BD Pharmingen	564943	FC
PE-Cy7 anti-Human CD86	BD Pharmingen	561128	FC
APC anti-Human CD206	BD Pharmingen	550889	FC
